# The Genus Commiphora: An Overview of Its Traditional Uses, Phytochemistry, Pharmacology, and Quality Control

**DOI:** 10.3390/ph17111524

**Published:** 2024-11-12

**Authors:** Yujia Yang, Xiuting Sun, Chuhang Peng, Jianhe Wei, Xinquan Yang

**Affiliations:** 1 Institute of Medicinal Plant Development, Chinese Academy of Medical Sciences and Peking Union Medical College, Beijing 100193, China; 15947122498@163.com (Y.Y.); sunxt202310@163.com (X.S.); 18061731341@163.com (C.P.); wjianh@263.net (J.W.); 2 Hainan Provincial Key Laboratory of Resources Conservation and Development of Southern Medicine, Hainan Branch of the Institute of Medicinal Plant Development, Chinese Academy of Medical Sciences and Peking Union Medical College, Haikou 570311, China

**Keywords:** myrrh, Commiphora, phytochemistry, pharmacology, quality control

## Abstract

Myrrh is the resinous substance secreted by plants of the genus *Commiphora*. In traditional Chinese medicine, Ayurvedic medicine, and traditional Arabic medicine, myrrh is regarded as an important medicinal material, widely used in the treatment of trauma, arthritis, hyperlipidemia, and other diseases. This review explores the evolving scientific understanding of the genus *Commiphora*, covering facets of ethnopharmacology, phytochemistry, pharmacology, artificial cultivation, and quality control. In particular, the chemical constituents and pharmacological research are reviewed. More than 300 types of secondary metabolites have been identified through phytochemical studies of this genus. Guggulsterone is a bioactive steroid isolated mainly from *Commiphora mukul*. The two isomers, *Z-* and *E-*guggulsterone, have shown a wide range of in vitro and in vivo pharmacological effects, including anti-proliferation, antioxidant, anti-inflammatory, and antibacterial. However, the current scientific research on quality control of medicinal materials and identification of original plants is insufficient, which limits the reproducibility and accuracy of biological activity evaluation experiments. Therefore, the establishment of analytical protocols and standardization of extracts is an important step before biological evaluation. At the same time, in order to find more bioactive substances, it is necessary to strengthen the research on the stems, barks, and leaves of this genus. The sources used in this study include PubMed, CNKI, Web of Science, Google Scholar, and other databases, as well as multinational pharmacopoeias, ancient books of traditional medicine, herbal classics, and modern monographs.

## 1. Introduction

Myrrh, a natural oil gum resin, is harvested from certain tree species of the genus *Commiphora*, dominated by *Commiphora myrrha* (T. Nees) Engl. or *Commiphora molmol* [1]. In the Bible, the resin of *Commiphora* species is called myrrh. Later, after being baptized by ancient Greek mythology, myrrh evolved into myrrha. It is mainly produced in Somalia, Ethiopia, the southern Arabian Peninsula, India, and other regions in Africa [2].

The medicine of myrrh has a special fragrance and was first known as a spice in the Tang Dynasty [3]. Its taste is punchy and bitter, and its character is flat. It has the effect of dispersing stasis and calming pain, reducing swelling, and generating muscle. As a traditional Chinese medicinal material imported from China, it has a long medicinal history. The chemical composition of myrrh is diverse, including volatile oils, terpenes, steroids, lignans, and other compounds [4]. Among them, volatile oil and terpenoids are characteristic components of myrrh and have various pharmacological activities.

Modern pharmacological studies have confirmed that myrrh has anti-inflammatory, antioxidant, analgesic, anti-tumor, antibacterial, and anti-Alzheimer’s disease properties, regulates lipid metabolism, promotes wound healing, and other activities [5,6,7]. Myrrh, as a medicinal herb with a long history and widespread application, not only holds an important position in the field of medicine, but also carries profound symbolic significance in religion. As a commodity with great medicinal value and economic value [8], its quality control plays a vital role in ensuring circulation.

However, existing reviews often focus on the pharmacological actions of the genus *Commiphora* but pay little attention to the quality control of its medicinal materials. The reproducibility and accuracy of biological activity evaluation experiments are directly constrained by the level of scientific research in quality control of medicinal materials and the precise identification of source plants.

This article presents a comprehensive review of research conducted on the traditional uses, phytochemistry, modern pharmacology, and other aspects of the genus *Commiphora*. At the same time, recommendations are made for quality control measures that will limit further research on the resins of *Commiphora* species. The aim of this review is to provide a reference for optimal application of the genus *Commiphora* in basic research, new drug development, and clinical treatment.

## 2. Materials and Methods

To comprehensively understand the current research status of the resins of Commiphora species, we searched Google Scholar, Web of Science, PubMed, CNKI, SpringerLink, ScienceDirect, and Baidu Scholar using keywords such as “MOYAO”, “*Commiphora*”, “Myrrh”, “*Guggul*”, “*གུ་གུལ།*”, and “*Gu Gu Le*”. Additionally, information was obtained from classical Chinese herbal medicine books and the multinational pharmacopoeia. A total of more than 500 articles from 1960 to 2024 were retrieved. Kew Science database (https://www.kew.org/science, accessed on 16 August 2024), Flora Reipublicae Popularis Sinicae database (http://www.iplant.cn/foc, accessed on 16 August 2024), and World Flora Online database were used for a comprehensive and systematic grasp of the taxonomic and morphological features of the genus *Commiphora* (http://www.worldfloraonline.org/, accessed on 16 August 2024). ChemDraw 22.0 software was utilized to determine the chemical structures of the compounds isolated from myrrh.

## 3. Species Distribution

**Figure 1:**
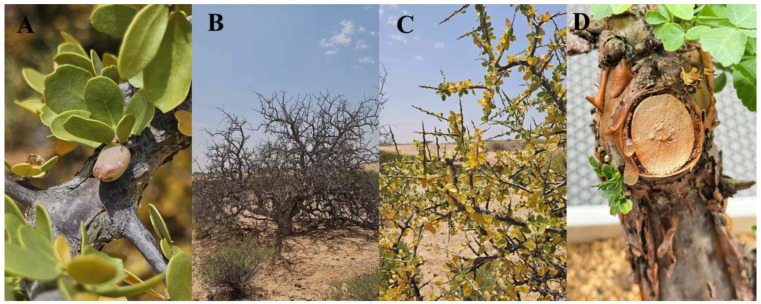
Botanical characterization of *Commiphora Myrrha* (**A**) leaves and fruit; (**B**) leafless, dormant tree; (**C**) green, leafy tree; (**D**) stem with thin, papery bark.

The Burseraceae family is comprised of approximately 700 species in 18 genera, with the genus *Commiphora* being one of them [9]. The genus *Commiphora* is a low shrub or tree, with heights rarely exceeding 3 m, and with many irregular spiky coarse branches. When the bark of these plants is damaged, they secrete an aromatic oleogel resin [10]. Our photo of the genus *Commiphora* is shown in Figure 1.

**Figure 2:**
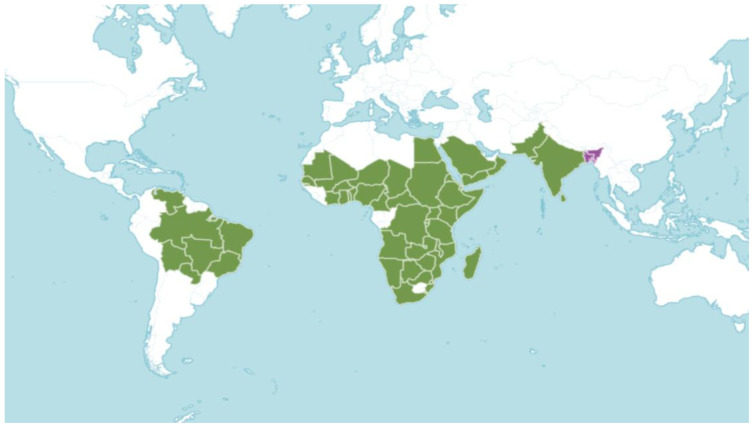
Green indicates the native distribution of the *Commiphora* species, and purple indicates the introduced native distribution of the *Commiphora* species.

The genus *Commiphora* includes about 150–200 taxa (species, subspecies, and varieties) that primarily occupy warm climates, especially within the tropics [11]. It occurs from southern Africa, eastward through tropical east Africa and the Horn of Africa, into the Arabian Peninsula, and with the northern limits of its distribution in the dry areas of Iran, Pakistan, and India. Refer to Figure 2 (published on the Internet; https://powo.science.kew.org/, accessed on 1 August 2024). One species, *C. leptophloeos,* occurs in the Americas, specifically in southeastern Brazil. It was originally described as a *Bursera,* but morphological and molecular studies have confirmed it to be a *Commiphora* [12].

Currently, the tree species that have been extensively studied mainly include *Commiphora myrrha* (T. Nees) Engl., *Commiphora myrrha* (Nees) English Var. molmol, *Commiphora optobalsamum* (L.) Engl., *Commiphora mukul* (Hook. ex Stocks) Engl., and *Commiphora erlangeriana* Engl. et al. [1]. The widely accepted distribution of *Commiphora* varieties and their traditional medicinal uses are summarized in Table 1.

Although the genus *Commiphora* has attracted more and more attention as a medicinal resource, its plant resources have not been properly protected. On the contrary, due to over-exploitation, these plants are gradually showing a dangerous tendency toward extinction. In the case of *C. wightii*, the plant’s oil gum resin plays an important role in international trade. However, the current harvesting methods have caused great damage to the trees and do not meet the conditions for sustainable harvesting [14]. For example, to increase resin production, ethephon is used in the cut where the glue is taken [15]. In the long run, this approach will interfere with the normal metabolic process of plants and ultimately accelerate their death.

Due to the destructive commercial development of the international oil gum resin trade, the population has directly declined and some states in India (Rajasthan) have been listed as critically endangered [16]. Meanwhile, *C. wightii*’s status on the IUCN Red List of Threatened Species has been changed from Data Deficient (DD) to Critically Endangered (CE) [17]. After India imposed an export ban, instead of adopting new sustainable mining methods, the problem shifted to other exporting countries, such as Pakistan [18].

The extraction of the resins of *Commiphora* species is closely related to the protection of plant resources, ensuring the sustainability of resin extraction is crucial for maintaining ecological balance and long-term utilization of plant resources. Growing demand combined with unscrupulous mining has threatened the survival of the species in the wild. As a wild plant, there is no selection of germplasm. At the same time, breeding programs have not been initiated due to the lack of systematic breeding and conservation programs. Excessive and unscientific harvesting has led to the depletion of the genus *Commiphora* in the wild. The genus *Commiphora* has a long dormant period, and the plants are leafless for a long time. Traditional methods of propagation, namely, seeds, cuttings, and air layering, have many limitations [19]. Therefore, the application of modern biotechnology tools needs to be standardized to maximize the use of this plant with important medicinal properties. Micro production by bud proliferation and somatic embryogenesis [20], as well as the production of secondary metabolites (guggulsterone) in callus culture and bioreactors, is currently being studied [21]. The existing methods have demonstrated the potential of micropropagation, but there are some limitations such as slow growth, heavy contamination of explants, low rooting rate, and low survival rate [22]. Similarly, somatic embryogenesis has limitations such as asynchronous development and low conversion rates. Difficulties in obtaining ovules in hard fruit dissection, low frequency of embryo reaction in zygotic embryos [23], yellowing and death of explants during culture, and genotype differences between varieties during in vitro culture are other limitations. As a slow-growing plant, with a yield only after 7–15 years, it is not suitable as a tree for social forestry. Due to the lack of cultivation, natural regeneration is almost negligible compared to natural consumption. Care must be taken to prevent its exploitation and to ensure its proper management and conservation.

## 4. Traditional Uses of Myrrh

### 4.1. Non-Medicinal Use

Myrrh, which appears in the Bible, is one of the famous gifts that the three Eastern philosophers brought to the newborn Christ (the other two being gold and frankincense), symbolizing the brevity of life, as it was then commonly used to embalm corpses [24]. Because of its special aroma, “myrrh” became an important “spice” used to make incense powder, balm, incense oil, etc. [25], and sometimes replaced “frankincense” for sacrifices. Myrrh oil has been used in traditional healing and religious rituals for thousands of years [26].

As early as 4000 years ago, myrrh was a commonly used medicinal herb, sacrificial offering, and material for smearing remains in ancient, civilized countries. It was used as perfumes and incense in the religious rituals of the Greeks, Romans, and Egyptians [27,28]. Myrrh is mainly produced in Somalia, Ethiopia, and the southern Arabian Peninsula. However, 90% of Somalia’s myrrh is used for export, with a low domestic use rate [29], and is mostly concentrated in marriage and daily household use: incense is used in traditional weddings [30].

Around 2500 B.C., the ancient Egyptians used myrrh for worship, cleansing, meditation, mummification, and skin care [31].

*Materia Medica from the [Southern] Seaboard Area*, written by Li Xun of the Tang Dynasty, has long been lost, and only a part of it has been preserved in the pharmacopoeia of later generations. According to the book, “Myrrh: *Annals of NanZhou*” by Xu Biao, “it originated in Persia and was also known as pine resin.” After being introduced to China, it was not only used as a traditional Chinese medicine but also became an important industrial raw material because it could be dissolved in alcohol and turpentine to make fake lacquer.

The oleoresin exudates of *C. myrrha* Engl. are widely used in the food industry for the production of industries for the production of beverages, chewing gums, candies, gelatins, nut products and confectionery, puddings, and canned vegetables due to a number of attractive properties such as good adhesive thickener, emulsifier, fixative, flavoring, and perfect stabilizing agent [32]. The Food and Drug Administration, USA, has approved the use of myrrh in foods (21 Code of Federal Registration-CFR172.510), while the Council of Europe has included myrrh in the list of plants and parts thereof that are acceptable for use in foods [33].

### 4.2. Medicinal Use

It is recorded in Chinese Pharmacopoeia 2020 (ChP2020) that myrrh has the effect of dispersing stasis and calming pain, reducing swelling, and generating muscle. It is used to treat chest arthralgia and heartache, gynecological benign tumors, dysmenorrhea, postpartum stasis, abdominal pain, rheumatic arthralgia, trauma, abscess, swelling, and wounds. The indications in accordance with modern pharmacological studies are described in detail in the following chapters.

Myrrh was first published in the Tang Dynasty’s *Theory of Medicinal Properties* (Yao Xing Lun), with “Myrrh” as its proper name, which was widely used in later generations of Chinese herbal medicine [34]. The *Theory of Medicinal Properties* said, “It is mainly used to treat injuries from beating, blood stasis in the heart and abdomen, bruises and falls, stasis and pain in tendons and bones, and pain caused by gold blades that is unbearable, and all of them are treated with wine”. The Song Dynasty *Illustrated Classic of Materia Medica* (Bencao Tujing) said [35], “Myrrh, born in Persia, Hainan and Guangzhou may have, the roots of the tree are like olives, the leaves are green and dense. For a long time, there is a paste liquid dripping in the ground, condensed into blocks, large or small, also like benzoin, picking time. It is also used in the treatment of internal injuries and pains of women, as well as in the treatment of postpartum anemic fainting and pain in umbilical region”. The Ming Dynasty *Compendium of Materia Medica* said [36], “Frankincense activating blood, myrrh scattering blood, they are both able to relieve pain and swelling, and promote wound healing, so the two drugs are often used in combination”. The Qing Dynasty Materia Medica basically continued the description of the efficacy and origin of myrrh in the previous Materia Medica. Frankincense is good at promoting blood and stretching the tendons, myrrh is partial to active blood and can disperse stagnation, they both can unblock blood stasis, and the two drugs are combined in a number of famous prescriptions. Table 2 summarizes several classic Chinese herbal preparations containing myrrh.

Chinese traditional medicine is a general term for the medicine of various ethnic groups, including traditional Chinese medicine, Tibetan medicine, Mongolian medicine, Uyghur medicine, Dai medicine, Miao medicine, Zhuang medicine, and other ethnic and religious medicine. Among them, Tibetan, Mongolian, and Uyghur medicine is an important part of ethnic medicine. Guggul is called “Gu Gu Le” in Tibetan and Mongolian medicine [37]. Guggul first appeared in the Dictionary of Tibetan Medicine. Gu Le is translated into Tibetan, Chinese name, “Hei Yun Xiang”, alias “Hari-Gu Gu Le”, is commonly used in Mongolian medicine, with the effect of removing stinging pain, reducing swelling, removing bruises, and so on [38]. It is recorded in the classic medical books of Mongolian and Tibetan medicine throughout the ages. According to scholars, the ancient *Gu Le* used in Mongolian medicine was called Hei Yun Xiang, which was the dried resin of *C. mukul*, the *Commiphora* plant in the Burseraceae family [39]. The Hari- Gu Gu Le equivalent refers to the natural myrrh in the Chinese Pharmacopoeia. The myrrh in Tibetan medicine is called གུ་གུལ། (Ge Ge), Mukul myrrh, and Kul myrrh, which are used for the treatment of anthrax, acute and chronic liver diseases, leprosy, stroke, plague, and other diseases [40]. In Uyghur medicine, *C. mukul* (known as Kai Li Mu) has the functions of reducing swelling, strengthening tendons and nourishing muscles, relieving cough and phlegm, and moistening the bowel [41,42].

The practice of Ayurvedic medicine in India can be traced back to the Vedic era of 5000 BC. It is noted for being the oldest recorded system of integrative medicine in the world. About 2300 years ago, Ayurveda, the first medical book in ancient India, described nearly 2000 herbal medicines [43], including Guggul Resin, which was used to treat various diseases, including obesity, osteoarthritis, arthritis, constipation, liver disease, inflammation, anemia, diabetes, and so on [44,45,46]. In addition to being processed and administered directly, Guggul resin is also prepared into essential oils and tinctures. When preparing a tincture of guggul resin, ethanol is often used as a solvent for extraction [47]. The tincture of myrrh has been recorded in European Pharmacopoeia (EP), United States Pharmacopoeia (USP), and Indian Pharmacopoeia (IP).

In Arab countries, the *Commiphora* tree is commonly referred to as myrrh. Widely used in traditional medicine systems to treat stomach aches, colds, fevers, and malaria, it also promotes wound healing, acts as an antiseptic, and is effective against skin infections [4]. The Arab world is rich in myrrh species, and different species are used in different ways for medicinal purposes. *C. erythrea*’s resin is used on livestock to prevent tick bites, as well as to relieve snake venom poisoning [48]. The boiled leaves of *C. gileadensis* are often used to treat abdominal pain [49,50]. The extracts of *C. gileadensis* resin are used to treat headaches, urinary retention, jaundice, constipation, stomach, and liver diseases, joint pain, and inflammatory disorders [51]. In addition, *C. myrrha* plays a role in the treatment of colds and chest pain, and it helps to strengthen head bone development in children [52].

## 5. Phytochemistry

To date, more than 300 secondary metabolites have been isolated from the genus *Commiphora*, mainly terpenes such as monoterpenes, sesquiterpenes, and triterpenes, as well as steroids, lignans, and sugars. Most of the bioactive compounds are extracted from the resins. The stems and leaves of the *Commiphora* genus also contain bioactive metabolites, but there are few studies on them.

Regarding the chemical composition of plants of this genus, the following features deserve our full attention. (1) Terpenes, especially sesquiterpenes and triterpenes, are the most abundant components in this genus. There are similarities and differences in the intrinsic components of different types of myrrh, such as *C. myrrha,* which contains more abundant sesquiterpene lactones and cyclic triterpene lactones, while *C. mukul* contains a large amount of silymane-type diterpenes. (2) *C. mukul* contains significantly higher levels of guggulsterone than other varieties. Studies on the chemical composition of myrrh and the application of combination with other drugs mainly focus on steroids as bioactive substances. (3) Substances such as polysaccharides in myrrh also have good biological activities that deserve further study (Supplementary Materials).

### 5.1. Terpenoids

The key substances for the effectiveness of myrrh are the terpenoids in myrrh, especially the volatile oil components.

By using the gas chromatography–mass spectrometry (GC-MS) technique, Dekebo studied and analyzed the volatile oil components of natural and colloidal myrrh, and found that the monoterpene components include limonene, basilene, etc. With the deepening of research [53], geranylene, α-pinene, β-pinene, camphene, and other components were also discovered [54]. Sesquiterpene is not only the main component of volatile oil but also the main component of pharmacological activity. The main structural types of parent nuclei are germacrene-type [55], endesmane-type [56], guaiane-type [57], cadinene-type, and elemene-type [58]. There are common furan rings, methylation, and acetylation in the structure.

**Figure 3:**
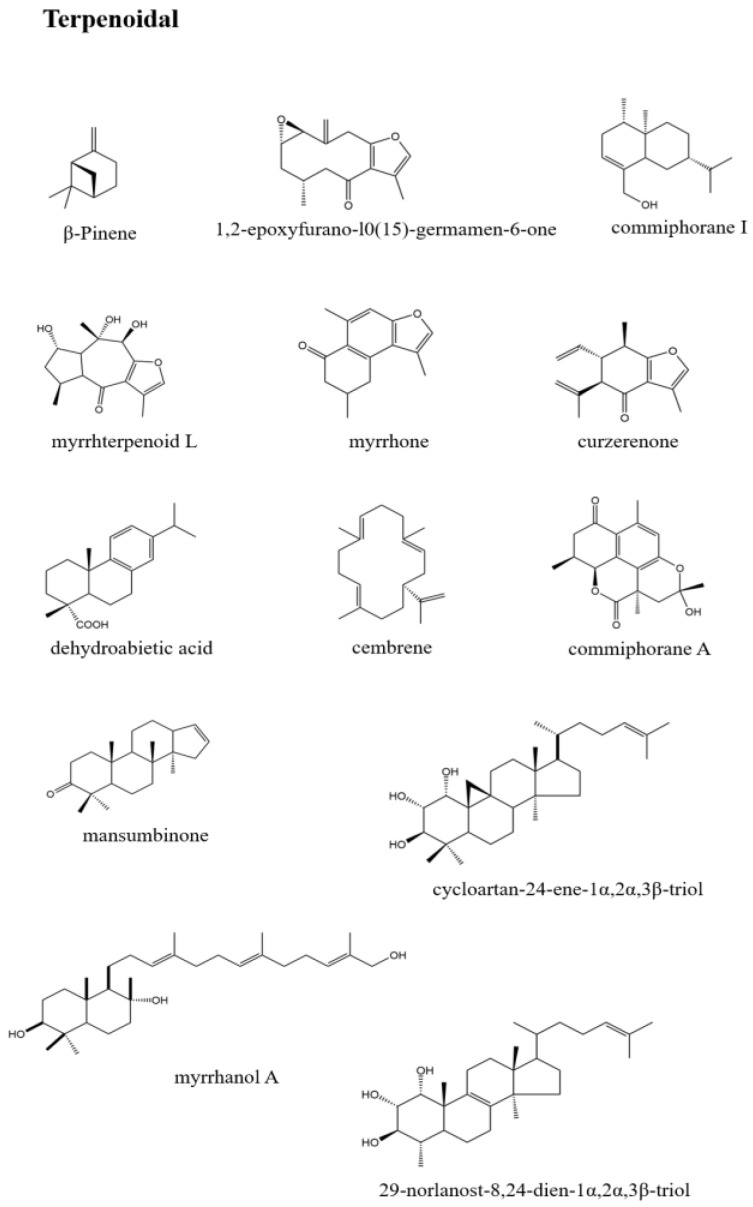
Representative terpenoid chemical structures.

Meanwhile, Wang Yong’s [59] research showed that β-caryophyllene, β-elemene, γ-elemene, and other low-oxidation sesquiterpenes accounted for the majority, among which β-elemene accounted for the highest proportion [60]. Most of these compounds are characterized and identified by GC-MS, influenced by variety, origin, and processing methods, the types and contents of components obtained by different experiments are different. The structure types of diterpenoids are relatively simple and can be divided into abietane/nor-abietane [61], cembrenoid type [62], podocarpen type [61], pimarane type [63], and 6/6/6/6 ring neoskeleton type [64]. The main types of the core structure of triterpenoids isolated from myrrh include oleanane [65], ursane [61], lanostane [66], cycloaltane, etc. Dammarane is the most common [67]. The terpenoids of various *Commiphora* genus are listed in Table 3. Some representative chemical structures are shown in Figure 3, while the remaining chemical structures can be found in the Supplementary Materials.

### 5.2. Lignans

**Figure 4:**
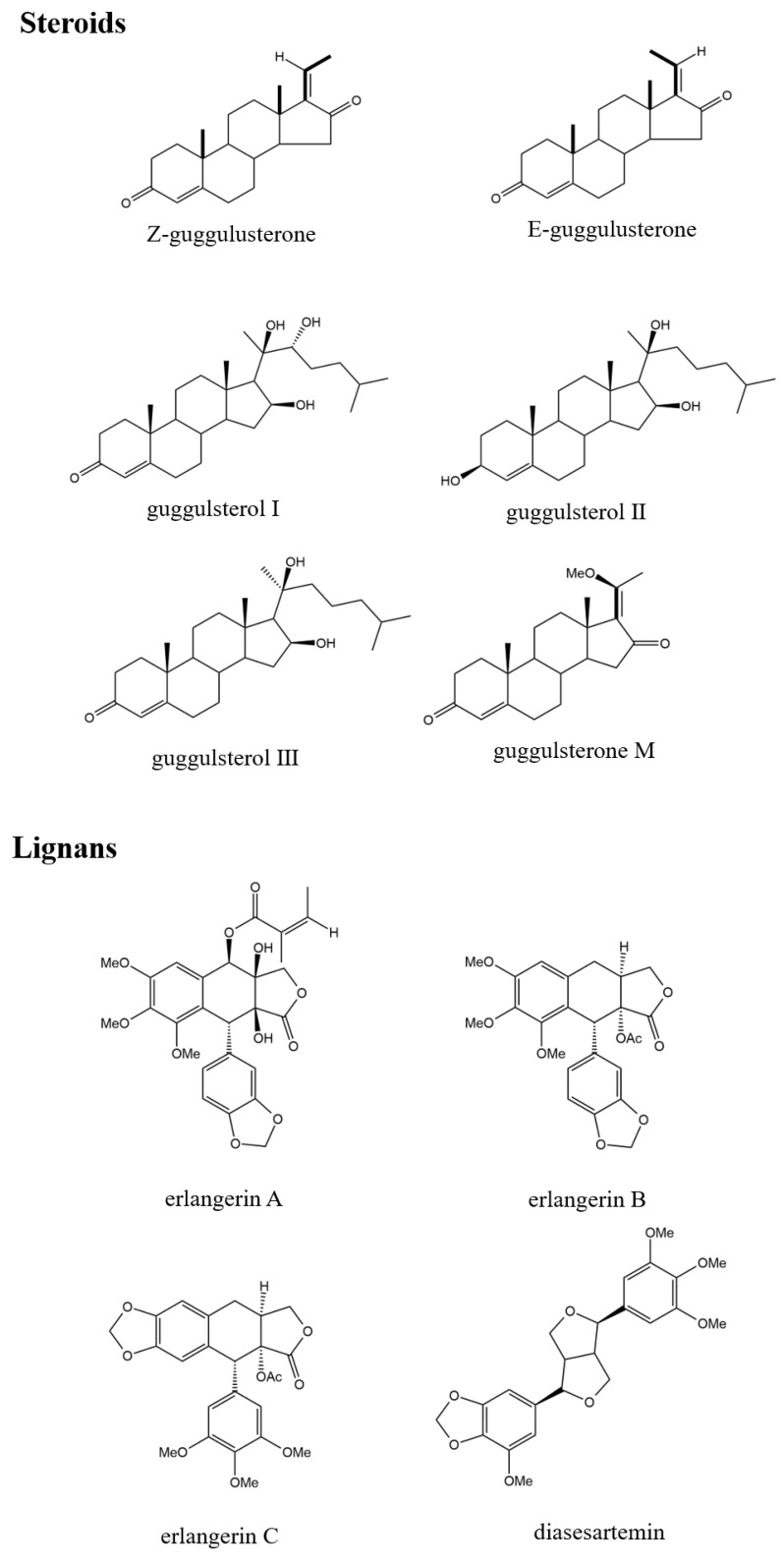
Representative chemical structure of lignans and steroids.

Aman Dekebo isolated four new lignans from the resin of *Commiphora* erlangeriana grown in Ethiopia and Somalia, two of which are polyphenolic types named erlangerin A and B, and the other two are podophyllotoxin types named erlangerin C and erlangerin D [69]. Podophyllotoxin and deoxypodontotoxin are secondary metabolites of many plants. Podophyllotoxin, as a lead compound in the development of novel anti-cancer drugs, is well known for its biological activity and importance [110]. The problem associated with its use is the scarcity of quantities isolated from natural sources. For this reason, the biotechnological production of this lignan has been studied. Deoxypodophyllotoxin is a promising anti-cancer agent. *C. mukul* (Burseraceae) is widely distributed in Pakistan and India [62]. Sultana isolated and structurally resolved a novel lignan (+)-commiphorin from the ethyl acetate extract of *C. mukul* resin from Pakistan [111]. The lignans of various *Commiphora* genus are listed in Table 4. Some representative chemical structures are shown in Figure 4.

### 5.3. Steroids

To date, more than 20 steroidal constituents have been isolated from myrrh, with *C. mukul* being the most abundant. Guggulu is a resin mixture secreted by the tree *C. mukul*. Patil et al. isolated two steroids from *C. mukul*, two isomers of Z- and E-guggulsterone [113]. In recent years, research on the pharmacological activity of guggulsterone has focused primarily on these two compounds. In subsequent studies, Patil isolated a new component, guggulsterols I–III, from the resin [114]. In the following years, scientists classified substances with similar structures and named them guggulsterols IV–VI [7,113]. C. Steroid components, namely, guggulsterone-M and guggulsterone-Y, were also isolated from the resin of *C. wightii* (the circulating species name for *C. mukul*) [109]. The steroids of various *Commiphora* genera are listed in Table 5. Some representative chemical structures are shown in Figure 4.

### 5.4. Miscellaneous

Myrrh also contains a variety of other chemicals such as amino acids, sugars, and flavonoids [116,117] The flavonoids of this genus are found in the flower, stem, and bark [118]. Metabolomics studies indicate that guggul contains a variety of amino acids and their derivatives [119]. The gum of myrrh is similar to gum arabic, which is hydrolyzed to yield arabinose, galactose, and xylose [118].

## 6. Pharmacology

**Figure 5:**
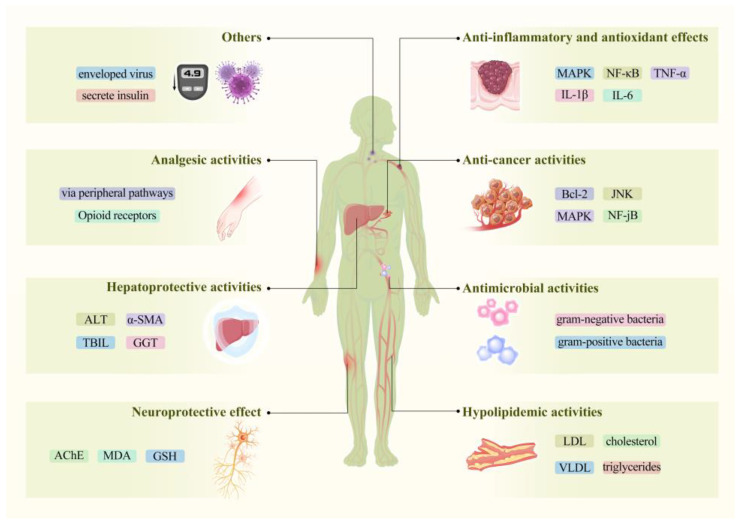
Distribution of pharmacological action and its key pathways and cytokines (Anti-inflammatory and Antioxidant: Suppresses pro-inflammatory cytokines (TNF-α, IL-1β, IL-6) and pathways (MAPK, NF-κB), anti-cancer: Induces apoptosis by downregulating Bcl-2 and modulating JNK, MAPK, and NF-κB pathways).

As mentioned above, various bioactive compounds have been identified in myrrh, indicating its potential as an ethnomedicine with significant research value. Studies have documented its range of biological benefits, including anti-inflammatory and antioxidant, anti-cancer, antimicrobial, hypolipidemic, neuroprotective, hepatoprotective, analgesic effects, and others. This section reviews and discusses the pharmacological effects of myrrh and its effective monomers Figure 5.

### 6.1. Anti-Inflammatory and Antioxidant Activities

Guggulsterone, a compound of steroidal parent structure, plays an important anti-inflammatory protective role in inflammatory diseases such as pancreatitis, rheumatoid arthritis, otitis media, enteritis, and neuritis.

A study on the effect of guggulsterone on cerulein-induced acute pancreatitis showed that pre-treatment with guggulsterone significantly reduced serum lipase levels in mice, inhibited the expression of TNF-α, IL-1 β, IL-6, and inhibited the infiltration of macrophages and neutrophils [120]. Nuclear protein activates mitogen-activated protein kinase (MAPK) and nuclear factor kappa-B (NF-κB) in the pancreas. However, intraperitoneal injections of guggulsterone suppressed the activation of extracellular signal-regulated protein kinase (ERK) and c-Jun N-terminal kinase (JNK) in the pancreas in cerulein-induced pancreatitis. Treatment with guggulsterone reduced elevated levels of these pro-inflammatory cytokines, but guggulsterone could not induce production of the anti-inflammatory factor IL-10. Song investigated the role of guggulsterone in inflammation of middle ear epithelial cells (HMEEC), and the results showed that uggulsterone can reduce the upregulation of TNF-α and COX-2 induced by lipopolysaccharide (LPS), which may be related to the inhibition of NF-κB activation [121]. The study by Gebhard showed that guggulsterone inhibits TF expression in vascular cells, which mediates inflammation in endothelial cells [122]. This inhibitory effect may be related to impaired activation of MAP kinases JNK and p38 (Mitogen-Activated Protein Kinase p38) in endothelial cells. Concerning the Cyclooxygenase (COX) inhibitory effect, E-guggulsterone is the most active, with 79% and 67% inhibition against COX-1, and with 83% and 54% inhibition against COX-2 at 100 ppm. Oxidative stress and inflammation are two major culprits in a variety of chronic diseases, including cancer. Upon receiving a signal of mild oxidative stress, cells are stimulated to express various antioxidant and phase II detoxification enzymes, collectively known as cytoprotective enzymes. Heme oxygenase-1 (HO-1), a representative cell-protective enzyme, has become an attractive target for the treatment of diseases characterized by high levels of chronic inflammation [123]. The effects of the steroid isomers E-guggulsterone and Z-guggulsterone on the expression of the cytoprotective HO-1 were investigated in human mammary epithelial cells (MCF10A). After incubation of MCF10A cells with E-guggulsterone or Z-guggulsterone at different concentrations (5, 10, or 25 μM) for 6 h, the expression of HO-1 was upregulated in a concentration-dependent manner, and the degree of HO-1 expression induced by E-guggulsterone was greater than that induced by Z-guggulsterone. HO-1 is a downstream target protein of Nuclear factor-erythroid 2-related factor 2 (Nrf2), which may play a protective role against various oxidative stress-induced damage to the Nrf2/HO-1 signaling axis. E-guggulsterone-induced HO-1 expression is mediated by increasing nuclear localization and antioxidant response elements (ARE) binding of Nrf2. E-guggulsterone induces Nrf2 nuclear accumulation and HO-1 expression through sulfhydryl modification of Phosphatase and Tensin Homolog (PTEN) and subsequent activation of Protein Kinase B (AKT) [124].

At the same time, myrrh also contains many other effective anti-inflammatory ingredients, such as myrrh alcohol, myrrh ketone, terpenes, etc. Numerous studies have confirmed that its methanol, ethanol, ethyl acetate extracts, etc., all have good anti-inflammatory activity.

Cembrane diterpenoids, polypodane triterpenoids, steroids, and lignans isolated from *C.wightii* have been tested for their NO production and COX inhibitory activities. Myrrhanol A and myrrhanone A prevented NO production with IC_50_ values of 21.1 and 42.3 mM, respectively [109]. Clinical studies have shown that *C.mukul* resin extracted at a dose of 500 mg TID for 1 month has a significant improvement in osteoarthritis, reducing pain, and stiffness, and improving function in patients [125]. In the evaluation of anti-inflammatory effects using the carrageenan-induced paw edema method, compared with the control group, administration of the methanol extract of *C. opobalsamum* stem significantly inhibited the increase in NO levels in the carrageenan response, inhibited the accumulation of PGE_2_ at the inflammatory site, and significantly reduced the level of TNF-α in the paw [126]. The mechanism of anti-inflammatory and antioxidant effects of methanol extract of *C. opobalsamum* is through inhibition of the expression of Malondialdehyde (MDA), NO, PGE_2_, and TNF-α in the inflammatory site. At the same time, Yang Bao extracted and separated the chemical components of myrrh and purified 15 compounds from the dichloromethane portion of the ethanol extract of myrrh. The main compounds were sesquiterpenes, and the anti-inflammatory activity of the 10 compounds with high isolation rates was evaluated in vitro. The anti-inflammatory activity was evaluated by detecting the inhibition of LPS-induced NO release in BV-2 cells by the compounds, with dexamethasone as a positive drug control group [127]. Meanwhile, 1(10),4-furanodien-6-one in the n-hexane extract of *C. erythraea* showed excellent anti-inflammatory activity. By exposing microglial BV-2 cells to lipopolysaccharide, we found that furanodiene-6-one pre-treatment restored cell viability and ROS to control levels, while halving NO generation. Pro-inflammatory IL-6, IL-23, IL-17, TGF-β, and IFN-γ were also significantly reduced after furanodiene-6-one treatment. Furanodiene-6-one exhibits anti-inflammatory properties in an in vivo model of microglial activation. In adult mice injected with LPS, we found that furanodiene-6-one has potent anti-inflammatory properties by inhibiting the expression of TNF-α and IL-1β in the liver and brain [128]. The ethanol extract of the resin of (CME) has a significant anti-inflammatory effect on the thickness (volume) of formalin-induced paw edema in rats. Mice were orally administered 125, 250, and 500 mg/kg of CME. Compared with the control group, the doses of 250 and 500 mg/kg of CME significantly reduced the volume of paw edema in rats at 3, 6, and 12 h after administration. The anti-inflammatory mechanism of CME may be related to the inhibition of the release of the inflammatory mediator PGs. The research results of Su et al. confirmed this explanation by comparing the anti-inflammatory and analgesic effects of 85% ethanol extract of *C. myrrha* with petroleum ether extract, ethyl acetate extract, n-butanol extract, and water extract. The experimental results showed that ethanol extract significantly inhibited the development of formalin-induced paw swelling. In the anti-inflammatory test, at a dose of 100 mg/kg, the pharmacological activity of petroleum ether extract was stronger than that of ethanol extract and other fractions, and it could significantly reduce the level of inflammatory factor PGE_2_ in the paw edema tissue at 4 h after formalin injection [129]. The above results suggest that the anti-inflammatory activity of myrrh may be involved in the anti-inflammatory effect by reducing PGs and peripherally mediated analgesic activity. The stem leaves extracts of *C. africana* (n-hexane, dichloromethane, acetonitrile, ethyl acetate, methanol, and n-butanol) showed good anti-inflammatory activity, which was achieved by inhibition of cyclooxygenase (COX-1 and COX-2). Polar extracts have the ability to scavenge free radicals and reduce iron (III). Acetonitrile and methanol extracts can significantly inhibit the production of prostaglandin [130].

The rich volatile oil contained in the resin of the *Commiphora* species, the anti-inflammatory activity of the volatile oil depends on the type and amount of chemical compounds present. The anti-inflammatory effects of aliphatic hydrocarbons, monoterpenes, and sesquiterpenes were increased in order. An evaluation of the volatile oils of *C. africana*, *C. habessinica*, *C. sphaerocarpa*, and *C. schimperi* revealed that *C. sphaerocarpa* resin is rich in the sesquiterpenoid α-copaene, β-caryophyllene, and β-caryophyllene oxide, and its anti-inflammatory activity is significantly higher than that of *C. africana* volatile oil, which is rich in monoterpenoids. *C. Spaerocarpa* resin exerts anti-inflammatory effects by inhibiting the NF-κB and MAPK pathways and activating the Nrf2/HO-1 pathway. The other two *Commiphora* species essential oils have no anti-inflammatory effects. Meanwhile, myrrh is often used in combination with incense [53].

HT083 refers to *P. lactiflora* root mixed with *C. myrrha* gum at 3:1 (*w*/*w*). Monodium iodoacetate (MIA) applied to the knee joint of male Sprague Dawley rats caused the same symptoms as Osteoarthritis (OA) in humans. HT083 can counteract elevated levels of IL-1β, a key pro-inflammatory cytokine in the progression of OA. In the LPS-stimulated RAW 264.7 model, HT083 significantly inhibited LPS-induced overproduction of NO and inflammatory cytokines such as IL-1β, IL-6, iNOS, and COX-2, thereby demonstrating good anti-inflammatory activity [131]. *C. myrrha* resin water extract (MWE) and the combined (*C.myrrha* resin. and *B. carterii* resin) extract (CWE) have inhibitory effects on formalin-induced paw edema at concentrations of 3.9 g/kg and 5.2 g/kg, respectively. The anti-inflammatory mechanism of CWE may be related to the inhibition of PGs and nitrite synthesis. CWE may be more therapeutically useful in reducing inflammatory pain than individual herbal extracts [132].

As summarized above, the resins of *Commiphora* species are the most studied and display evident anti-inflammatory properties both in vitro and in vivo. Steroids and triterpenoids are the main anti-inflammatory substances of the genus *Commiphora*. The mechanism of action related to multiple inflammation-related proteins and signal pathways have been discussed, and COX, NO formation, ROS, TNF-α, PGE_2_, NF-κB, and MAPK have been verified as potential anti-inflammatory targets.

### 6.2. Anti-Cancer Activities

Guggulsterone has not only shown superior pharmacological activity in anti-inflammatory activities but has also shown potential in the field of anti-cancer. The chemical structure of its steroid parent nucleus makes it have a significant effect on sex hormone-related cancer cells.

Using PC-3 human prostate cancer cells as a model, guggulsterone-mediated inhibition of PC-3 cell proliferation induces cell apoptosis but has no effect on normal prostate epithelial cell lines (PrEC). Guggulsterone-induced apoptosis was associated with the induction of multidomain proapoptotic Bcl-2 (B-cell Lymphoma-2) family members Bax (Bcl-2 Associated X Protein) and Bak (Bcl-2 Homologous Antagonist/Killer) [133]. The LNCaP and PC-3/DU145 cell lines, as typical representatives of androgen-responsive and androgen-independent human prostate cancer cells, can be observed as guggulsterone-induced cell apoptosis in both cell types. Guggulsterone-induced cell death in human prostate cancer cells is caused by activation of reactive oxygen species (ROI)-dependent JNK, but ROI is not produced in normal prostate epithelial cell lines (PrECs), which are also resistant to guggulsterone-mediated JNK activation [134]. Z-guggulsterone can block angiogenesis both in vivo and in vitro, and can be used to treat prostate cancer. Z-guggulsterone inhibits angiogenesis by blocking the VEGF-VEGF-R2-Akt signaling axis. After acting on human umbilical vein endothelial cells (HUVECs), Z-guggulsterone reduced the migration of HUVECs and DU145 human prostate cancer cells in a concentration and time-dependent manner [135]. GL is a chemical quantified based on Z-guggulsterone content as the standard. Taking human estrogen receptor-positive (MCF-7), triple-negative (MDA-MB-231) breast cancer cells and a normal human mammary epithelial cell line (HMEC) as experimental subjects, GL significantly inhibited the growth of MCF-7 and MDA-MB-231 cells. The apoptosis induced by GL was related to the downregulation of the β-Catenin signaling pathway. GL treatment resulted in a significant decrease in the β-Catenin/T-cell factor 4 (TCF-4) complex in two types of cancer cells, which enhanced GL-induced apoptotic cell death. Meanwhile, HMEC showed no significant response to the growth inhibition and induction of apoptosis of GL [136].

In head and neck squamous cell carcinoma (HNSCC) research, guggulsterone has the potential to inhibit activation of the NF-κB and signal transducer and activator of transcription (STAT) 3 pathways induced by smokeless tobacco (ST) and nicotine. Treatment of HNSCC cells with guggulsterone abrogated ST- and nicotine-induced nuclear activation of NF-κB and pSTAT3 proteins, as well as the downstream targets COX-2 and vascular endothelial growth factor [137]. In addition, guggulsterone treatment reduced interleukin-6 secretion by HNSCC cells. In the SENCAR mouse skin tumorigenesis model, it was found that local application of guggulsterone has anti-tumor-promoting effects, which are mediated by the ability of guggulsterone to modulate the MAPK and NF-κB pathways [138]. Z-guggulsterone treatment increased the expression levels of the PD-L1 (Programmed Death-Ligand 1) surface and mRNA, and gene transcription in non-small cell lung cancer (NSCLC) cells. Mechanism experiments have shown that in NSCLC cells treated with Z-guggulsterone, the upregulation of PD-L1 is partially mediated by farnesoid X receptor (FXR) inhibition, and partially by activating the Akt and Erk1/2 signaling pathways. In vivo, Z-guggulsterone treatment dose-dependently increased the expression level of PD-L1 in a mouse Lewis Lung Carcinoma (LLC) tumor model, with the potential to combine PD-1/PD-L1 antibodies for the treatment of NSCLC [139].

Multidrug resistance is one of the major causes of failure in tumor chemotherapy [140]. When tumor cells develop resistance to one chemotherapy agent, they often develop cross-resistance to others, known as multidrug resistance. The co-administration of guggulsterone can significantly increase the chemosensitivity of multidrug-resistant human breast cancer MCF-7/DOX cells to doxorubicin (DOX) in vitro. Establishing MCF-7/DOX and MCF-7 xenograft mouse models, the use of doxorubicin alone did not significantly inhibit the tumor growth of MCF7/DOX xenografts, indicating that they retained doxorubicin resistance. However, the use of doxorubicin alone can significantly inhibit the tumor growth of MCF-7 xenografts, indicating that it maintains sensitivity to doxorubicin. When doxorubicin and guggulsterone are co-administered, their anti-tumor activity is enhanced in MCF-7/DOX xenografts. Further research suggests that the inhibitory effect of guggulsterone on Bcl-2 and P-glycoprotein expression may be the reason for the increased chemosensitivity of MCF-7/DOX cells to doxorubicin in vivo. No significant toxic signs related to guggulsterone were found in weight, hematological indicators, liver, heart, and gastrointestinal histopathological examinations. Guggulsterone may reverse doxorubicin resistance in vivo without serious side effects [141].

Other substances in myrrh, such as terpenes and flavonoids, also have good anti-cancer activity. Shi Lingchun found that the myrrh sesquiterpene contains α, β, and γ elemene, and modern pharmacological and clinical studies have shown that elemene has a good anti-tumor effect. Beta-olive has been used as an anti-cancer drug to treat various cancers such as glioma, and its anti-proliferative effect on glioma cells is achieved by activating p38 MAPK [142].

Shen found that a series of cycloartane-type triterpenoids were isolated from *C. opobalsamum* and evaluated for their anti-prostate tumor activity against PC3 and DU45 cells, with IC_50_ values of 10.1~37.2 μmol/L [85]. Zhu found that rel-1S, 2S-epoxy-4R-furanogermacr-10 (15) en-6-one in myrrha has weak cytotoxicity to MCF-7 breast cancer cell line; IC_50_ value is 40 μmol/L [82]. Ali S. isolated two furano-sesquiterpenoids, 2-methoxyfuranodiene (CM1) and 2-acetoxyfuranodiene (CM2), from the chloroform fractions of the ethanolic extract of *C. myrrh*. The cytotoxicity of the compounds was evaluated using human liver carcinoma and breast cancer cell lines (HepG2 and MCF-7, respectively), and HUVECs. Cell viability assays showed that both compounds were highly cytotoxic in HepG2 and MCF-7 cells with IC _50_ values of 3.6 and 4.4 μM, respectively. At the same time, both compounds induced apoptosis and cell cycle arrest in HepG2 cells. The current research results indicate that furan sesquiterpenes exhibit significant inhibitory effects on cancer cell proliferation, which may be related to their mechanisms of inducing cell apoptosis and inhibiting angiogenesis [75]. Su found through experimental studies that *C. myrrha* showed significant anti-tumor effects on C6 glioma cells, A2780 cells, A2708 cells, Shikawa cells, and SK-OV-3 cells [143]. To investigate the possible chemoprophylaxis of ethanol extract of *C. molmol* resin in rats with early-stage hepatocarcinogenesis induced by diethylnitrosamine (DEN)/phenobarbital (PB). Treatment of the DEN/PB-induced rats with *C. molmol* resin extract can significantly reduce the level of circulating IL-6, suggesting that its anti-inflammatory effect can alleviate DEN/PB-induced hepatocarcinogenesis. In addition, *C. molmol* can also significantly improve the antioxidant defense ability of DEN/PB-induced rat liver by regulating the important Nrf2/ARE/HO-1 signaling pathway, thereby upregulating the expression of Nrf2 and heme oxygenase-1 [144].

Stems and leaves of *Commiphora* species were extracted with chloroform: methanol (1:1). The most active *Commiphora* species against the HT-29 cells (SRB anti-cancer assay) were *C. glandulosa* (leaf and stem) and *C. marlothii* (leaf). The MCF-7 cells (SRB anti-cancer assay) exhibited the highest sensitivity to indigenous *Commiphora* species, with *C. edulis* (leaf and stem), *C. glandulosa* (leaf and stem), *C. marlothii* (leaf), *C. pyracanthoides* (leaf and stem), *C. schimperi* (stem), and *C. viminea* (stem) all possessing a percentage inhibition greater than 80% at 100 μg/mL. *C. glandulosa* (leaf and stem) and *C. pyracanthoides* (leaf and stem) were the two most active species against the SF-268 cells (SRB anti-cancer assay), with IC_50_ values ranging between 68.55 ± 2.01 and 71.45 ± 1.24 μg/mL. The anti-cancer activity of the stem and leaf extracts may be flavonoids [145].

### 6.3. Antimicrobial Activities

Dorala showed antibacterial activity against Escherichia coli, Staphylococcus aureus, Pseudomonas aeruginosa, and Candida albicans by obtaining furadiene-6-one and methoxyfuran guaiacol-9-ene-8-one from the drug. The minimum inhibitory concentration for *Escherichia coli*, Staphylococcus aureus, and Pseudomonas aeruginosa is 0.18~2.80 mg/mL [146]. Ye demonstrated that the aqueous solution of myrrh (1:2) has an inhibitory effect on a variety of cutaneous fungi, which may be related to eugenol contained in volatile oil [147]. Mohamed Adam found that the methanol extract of *C. molmol* essential oil methanol extract had an inhibitory effect on both Gram-negative and Gram-positive bacteria [148].

Ayman Alhazmi demonstrated that *C. gileadensis* extract possesses antibacterial and anti-inflammatory properties that induce wound healing. *C. gileadensis*-methanolic extract has flavonoids, terpenoids, phenol, tannins, alkaloids, steroids, amino acids, glycosides, and saponins. Terpenoids have been reported to have an antimicrobial activity that induces re-epithelization and wound contraction. In addition, flavonoids and saponins have been proposed to have wound-healing activity. Moreover, flavonoids and glycosides possess an antioxidant activity that prevents lipid peroxidation by induction of angiogenesis. They also have anti-inflammatory and antibacterial activities that reduce cell necrosis and fibrosis. Finally, tannins were reported to be an inducer of re-epithelization. This property may induce wound healing [149].

### 6.4. Hypolipidemic Activities

Gugulipid, a standardized resin extract of *C. mukul*, has been sold in the market for the treatment of hyperlipidemia. The main active ingredients are the sterone-like components E and Z-guggulsterone, while other extract components, whether independently or synergistically, do not have lipid-lowering activity. The reason for this is that sterone-like components can activate lipolytic enzymes, inhibit the biosynthesis of hepatic cholesterol, and lower the levels of lipids and cholesterol.

Singh found that administration of guggulsterone can reduce serum cholesterol levels by 27% and serum triglyceride levels by 31% in rats, which is related to the enhanced liver uptake of LDL by guggulsterone [150]. Similarly, guggulsterone treatment improved fasting blood glucose, glucose tolerance, and plasma insulin levels, and reduced LDL, VLDL, cholesterol, triglycerides, and other harmful lipid levels in mice fed a high-fat diet, which may be related to the regulation of peroxisome proliferator-activated receptor-γ (PPARγ) [92] expression and activity by guggulsterone [151]. At the same time, some terpenoid metabolites in myrrh have lipid-regulating activities. They activate PPARα and carnitine palmityl transferase I (CPT1). Gautam A found that in in vivo and in vitro models of atherosclerosis, guggulsterone can reduce the level of rimethylamine (TMA)/flavin monooxygenases/trimethylamine-N-oxide (TMAO), lipid profile, atherogenic risk predictor (ARP) index, and biomarkers of liver and kidney injury in rats and play a role in lowering blood lipids [152]. Guggulsterone antagonizes the chenodeoxycholic acid-activated nuclear (FXR), which regulates cholesterol metabolism in the liver. Since mammalian cholesterol homeostasis is regulated by FXR in the liver for metabolism and by phospholipaseA2 (PLA2) in the intestine for absorption, modulation of PLA2 and FXR by bile acids and selected guggul components suggests novel opportunities for hypolipidemic and hypocholesterolemic therapies [153].

Ye Jianhong found that the oleoresin part of myrrh can reduce blood cholesterol and prevent the formation of atherosclerotic plaque in the intima of arteries, and experiments showed that the oleoresin part of the liquid decoction of myrrh (1:2) also has the effect of lowering blood lipids, and myrrh has the effect of preventing coronary heart disease [147].

### 6.5. Neuroprotective Activities

Gugulipid, an ethyl acetate extract of the resin of plant *C. wightii* is an established hypolipidemic agent in clinical practice. MDA is an important marker for lipid peroxidation and GSH activity is an indicator of free radical generation. The inhibition of acetylcholinesterase (AChE), a metabolizing enzyme of acetylcholine, in the brain is important for increasing cholinergic neurotransmission. Therefore, the use of cholinesterase inhibitors is the most effective pharmacological approach for the symptomatic treatment of anti-Alzheimer’s Disease (AD) [154]. Gugulipid treatment caused a significant decrease in AChE activity, a low level of MDA, and a high concentration of GSH in the brain following streptozotocin (STZ) as compared to vehicle administration in STZ-treated mice. Gugulipid has a significant protective effect against the streptozotocin-induced memory deficits model of dementia, which can be attributed to the antioxidant and anti-AChE activity of gugulipid. These observations suggest gugulipid as a potential anti-dementia drug (CDRI, Lucknow has obtained US patent No. 6896901 for the use of gugulipid as a cognitive enhancer).

Xu isolated sesquiterpenes (commiterpenes A–C) from myrrh resin, two of which showed neuroprotective effects against 1-methyl-4-phenylpyridinium (MPP+)-induced SH-SY5Y neuronal cell death. Specifically, guanine is used as a positive regulator of the lactose operon in human neuroblastoma cells and has a death-inducing effect on MPP+. The survival ability of neuroblastoma exposed to MPP+ was tested by MTT assay, and there was a significant difference (*p* < 0.01) [58]. The sesquiterpene compounds extracted from myrrh resin by Yu showed therapeutic effects in the pathological model of AD in the nematode worm (Caenorhabditis elegans) [89].

### 6.6. Hepatoprotective Activities

Bulleted lists look like this: Kim found that guggulsterone can inhibit the growth of immortalized human hepatic stellate cells LX-2, reduce the expression of collagen α1 synthesis and α-smooth muscle actin (α-SMA) in LX-2 cells, and play an anti-fibrosis role, which is mediated by the activation of c-Jun N-terminal kinase and the expression of mitochondrial apoptosis signal [155].

Alahmari demonstrated the antioxidant effect of natural *C. myrrha* on hepatotoxicity and oxidative stress induced by ethanol in adult male rats. The study found that treatment with *C. myrrha* after the oral consumption of ethanol caused a reduction in serum liver function parameters (alanine transferases, aspartate transaminase, and total bilirubin), hepatic tumor markers (α-L-flucosidase and arginase), and hepatic lipid peroxidation indicator (thiobarbituric acid reactive substances), as well as a slight restoration (not significant) in the levels of superoxide dismutase, catalase, reduced glutathione, and total antioxidant capacity. In addition, it alleviates histopathologic changes in the liver, as revealed by decreased areas of inflammatory infiltrate, milder necrosis, and noticeably reduced periportal fibrosis and hemorrhage [156].

Carbon tetrachloride (CCl_4_) has been widely used in animal models to investigate chemical toxin-induced liver damage. The increased level of the SOD, catalase, and GPx observed point to the hepatic damage in the rats administered with CCl_4_. However, the groups treated orally with methanolic extract of *C. berryi* bark showed a significant decrease in the level of these enzymes, which indicates protective effects on the liver of *C. berryi* [157]. Pre-treatment with the resin of *C. opobalsamum* could shorten the barbiturate sleeping time and replenish the non-protein sulfhydryl of the liver caused by CCl_4_-induced live damage [158].

### 6.7. Analgesic Activities

Before the discovery of morphine, myrrh was a common analgesic. Dorala found that the sesquiterpene components of myrrh, furunesin-1, 3-diene and curcumene, can act on opioid receptors in the central nervous system and have analgesic activity, and their effects can be blocked by the morphine-antagonist naloxone. It is believed that the potent analgesic effect of myrrh extract may act on opioid receptors in the brain like morphine, but without the side effects of morphine dependence [159].

The acetic acid-induced writhes and hot plate test methods have been postulated for useful techniques of evaluating the peripherally and centrally acting analgesic drugs, respectively. The results indicated that 85% ethanol extract of *C. myrrha* and petroleum ether extract significantly inhibited the number of writhes in comparison with the control group (*p* < 0.01; *p* < 0.05, and *p* < 0.001). However, all test samples showed no significant effects in the hot-plate model [129]. Taken together, the ability of the myrrh extracts to suppress pain perception in the acetic acid test might be mediated via peripheral pathways of pain perception, not a central one. The results of the dysmenorrhea test indicated that myrrh water extract significantly inhibited the number of writhing in comparison with the control group (*p* < 0.01). The active components were identified for sesquiterpene, diterpene, and triterpenic acids [132].

The analgesic activity of the *C. opobalsamum* stem was determined by the acetic acid method, hot plate method, and formalin licking claw method. The results showed that the methalone extract of *C. opobalsamum* stem also showed good analgesic activity [160].

### 6.8. Others

*E-* and *Z-*guggulsterone were isolated from the methanol extract of *C. gileadensis* leaves, and this activity was evaluated by plaque reduction assay and 3-(4, 5-dimethylthiazole-2-yl)-2, 5-diphenyl tetrazolide bromide assay against both enveloped and nonenveloped viruses, respectively. The methanol extract of *C. gileadensis* leaves has antiviral activity only against the enveloped virus, and this molecule may interact with the specific receptor of the enveloped virus to show a viricidal effect [51]. Valentina Noemi Madia’s research indicates that *C. myrrha* resin extract has anti-influenza A Puerto Rico 8/34/H1N1 virus activity, but also has a killing effect on cells infected with the virus. When combined with VE ester, it can alleviate the killing effect on its own cells [161].

Rasha A. Mansouri found that the aqueous extract of *C. myrrha* resin had a therapeutic effect on the pancreas of streptozotocin-induced (STZ) diabetes in the female Sprague Dawley rats. After induction, microscopic examination of the rats showed that the pancreas was degenerative and atrophic, and the exocrine ducts were dilated. After treatment, Langerhans islets were close to normal, without any histopathological changes. Myrrh extract is considered an oral hypoglycemic drug [162]. Shokoohi conducted a double-blind, placebo-controlled study and found that the combination of *C. myrrha* and other herbal compounds reduced fasting blood glucose and improved blood lipids in women with diabetes [163]. On this basis, Al-Romaiyan investigated the direct effect of *C. myrrha* on insulin secretion to elucidate the hypoglycemic mechanism of *C. myrrha* in vivo. Research has shown that *C. myrrha* resin directly stimulates β-cell lines and isolated primary pancreatic islets to secrete insulin [164].

## 7. Quality Control

Quality control is an important link to ensure the curative effect of Chinese medicinal materials, and it is the standard to supervise the quality of Chinese medicinal materials. The standards of myrrh in Chinese Pharmacopoeia (ChP 2020) [165], United States Pharmacopoeia (USP2024-NF42) [166], European Pharmacopoeia (EP11.0) [167], British Pharmacopoeia (BP2024) [168], and Indian Pharmacopoeia (IP2020) [169] are summarized in Table 6, among which the common provisions include species origin, character identification, qualitative analysis, impurity limitation, etc.

### 7.1. Species Origin

The name myrrh has many meanings. The Chinese Pharmacopoeia (ChP) refers to myrrh, which includes two types: natural myrrh and colloidal myrrh. Its herbal source is the dried resin of the plant *C. myrrha* or *C. molmol* of the Burseraceae family. The European Pharmacopoeia (EP) and the British Pharmacopoeia (BP) refer to myrrh, the plant source of which is the resin produced by the Burseraceae plant *C. myrrha* and other plants of the Burseraceae family, including the tree species *C. mukul*, and do not specify the source of the tree species. The Indian Pharmacopeia (IP) refers to the resin produced by *C. wightii* (a circulating species name for *C. mukul*) as guggul resin rather than myrrh, and does not specify the origin of other tree species. The United States Pharmacopeia (USP) defines the resins produced by *C. myrrha* and other members of the Burseraceae family as myrrh, and the resins produced by *C. wightii* as guggul.

There are about 190 species of Burseraceae *Commiphora* in the world, most of which are distributed in eastern and southern Africa and Madagascar, while a few are distributed in western Africa, Iran, Pakistan, the Indian Peninsula, Sri Lanka, and Brazil. Many plants of the genus *Commiphora* can produce resins, which are not only a means of self-protection for the plants, but also attract attention for their unique medicinal value. However, there have always been problems in identifying their origin. Taking the plants *C. mukul* and *C. wightii* as examples, they are the same tree species, *Commiphora wightii* (Arnott) Bhandari syn. “*Commiphora mukul* (Hook.ex Stocks) Engl, but because of their different names in circulation, the resins they produce are often considered to be two different myrrh. This misunderstanding not only affects the proper use of the medicine, but also prevents further research and development of its medicinal value. In addition, there are differences in the regulations for the genus myrrh between national pharmacopoeias. This difference leads to the unclear classification of varieties in the circulation of medicinal materials, which makes the international trade and exchange of medicinal materials complicated and difficult.

### 7.2. Identification of Characters

The characteristics of natural and colloidal myrrh are described in detail in the ChP. Natural myrrh is blocky, with a yellowish-brown or reddish-brown surface, a brown-black almost translucent part, and yellow dust. The quality is hard and brittle, the broken surface is uneven, and the luster is dull. It has a specific aroma with a bitter and slightly pungent taste. Colloidal myrrh is darker in color, firm or loose, and sticky.

The USP describes the appearance of myrrh as brownish-yellow to reddish-brown, with gray or yellow dust; the interior is rich in brown or reddish-brown, sometimes with white spots or lines; thin fragments that are semi-transparent or almost transparent; and has a specific aroma, bitter taste, slightly spicy. Guggul is described as a block of varying sizes, light-to-dark brown, and slightly sticky to the touch, with a unique aromatic aroma and bitterness.

IP description Guggul resin is a light-to-dark brown resin, round or irregularly clumped, sticky, and aromatic. EP and BP are less descriptive than other pharmacopoeias, describing it only as slightly bitter.

In summary, although different pharmacopoeias have different descriptions of the characteristics of myrrh and its related varieties, they all highlight its unique color, texture, and odor, providing an important reference for the identification and use of medicinal materials.

### 7.3. Qualitative Analysis

The identification of myrrh in the pharmacopoeias of different countries is mainly based on thin-layer identification and chemical methods. Although EP and BP do not have specific regulations on the species origin of *C. mukul*, their methods of identification are different from those of other tree species. Other myrrh species mainly rely on morphological or microscopic identification, while *C. mukul* myrrh specifically uses the thin-layer identification method. In Guggul’s thin-layer identification, both USP and IP chose (Z and E)-myrrhosterone as the standard, which may be because *C. mukul* myrrh has a higher content of guggulsterone compared to other myrrh resins. In addition, USP has added high-performance liquid chromatography (HPLC) for identification.

### 7.4. Determination of Content

The main chemical substances that exert medicinal effects in myrrh are secondary metabolites such as monoterpenes, sesquiterpenes, and triterpenes, most of which are soluble in organic solvents. In order to ensure the stable efficacy of myrrh and meet the treatment needs, pharmacopoeias of various countries have made clear regulations on its quality indicators. The ChP clearly stipulates the volatile oil content of natural and colloidal myrrh: the former should not be less than 4% (mL/g) and the latter should not be less than 2% (mL/g).

In the EP and BP, it is stipulated that the alcohol-insoluble substance content of myrrh should not exceed 75%, in order to control the content of impurities in the medicinal material. The USP has more detailed quality requirements for myrrh, requiring that the content of alcohol-soluble extracts should be between 40 and 70%, the content of water-soluble extracts should not be less than 50%, and the content of volatile oil should not be less than 6.0%. For Guggul, its alcohol-soluble extract content should not be less than 33%.

The IP provides clear regulations for the content of ethyl acetate soluble extract and ethanol soluble extract in guggul resin, with the former not less than 25.0% and the latter not less than 35.0%. In addition, in terms of impurity control, pharmacopoeias of various countries also have certain requirements for total ash content, acid-insoluble substances, etc., to further ensure the purity and quality of medicinal materials.

## 8. Conclusions and Future Perspectives

This paper reviews research on Commiphora resins in the Burseraceae family, focusing on traditional uses, phytochemistry, pharmacology, and quality control to support further studies on myrrh. Myrrh demonstrates a broad range of biological activities, with over 300 compounds—such as terpenes, steroids, and polysaccharides—identified to date. However, the specific mechanisms of many compounds, especially when myrrh is used alongside other Chinese medicines, require further investigation.

Despite myrrh’s extensive history in incense, pharmaceuticals, and other industries, current quality standards in pharmacopoeias are limited mainly to impurity, extract content, and ash limits, which lack specificity and fail to fully represent the diverse qualities from different sources. Myrrh’s composition varies depending on plant origin and other factors; for instance, *C. myrrha* is rich in sesquiterpene lactones, whereas *C. mukul* contains higher steroidal compounds.

Moreover, the chemical composition of myrrh varies with the stage of collection, plant origin, as well as geographical and climatic conditions. Therefore, based on the systematic sorting of the chemical components of myrrh, it is essential to explore additional chemical and biological activity markers closely associated with its quality to establish scientific and robust quality control methods. This approach will lay a stronger foundation for the development and application of myrrh.

Finally, the harvesting of resins of *Commiphora* species is closely linked to the conservation of plant resources. Ensuring sustainable resin extraction is crucial for maintaining ecological balance and the long-term viability of plant resources. As an important cash crop, the artificial cultivation of the genus *Commiphora* can alleviate the survival crisis of wild populations, reduce excessive damage to natural resources, establish uniform quality standards, enhance market supply stability and traceability of medicinal materials, and advance standardization efforts. Furthermore, optimizing the cultivation environment can enhance the accumulation of secondary metabolites, thereby further improving the quality and efficacy of medicinal materials.

## Figures and Tables

**Table 1:** Distribution of *Commiphora* species.

Species	Area	Application
*Commiphora abyssinica*	China, East Africa, Ethiopia	Stem skin: treat scorpion sting. Preparation of oleo-gum-resin: dissipate blood stasis and pain, detumescence and muscle, convergence, drive wind, sweating, strong, diuretic, dispel phlegm, meridian. Used for hemostasis swelling pain, chest pain, ulcer, and soreness.
*Commiphora Africana* (A.Rich) Engler	Gambia, Ethiopia, Sub-saharan Africa	Tree exudate preparation of oleo-gum-resin: for spices, convergence, wind, diaphoresis, diuresis, expectorant, meridian. Roots: Used in Tanzania for mastitis, hernia
*Commiphora agallocha* Engler	India	Resin: Used as a substitute for myrrh
*Commiphora berryi* (Arn) Engl	India, Jordan, Egypt	Bark extracts: wound healing and inflammation
*Commiphora caudate* (Wight & Arn.) Engl	India, Sri Lanka	Resin: arthritis, hyperlipidemia, pain, healing of wounds, coronary artery, and gynecological diseases, and also widely used to treat painful inflammatory conditions.
*Commiphora boiviana* Engler	Somalia	Stem skin, root: oral decoction for lactation, aphrodisiesis, insect repellent, dysentery.
*Commiphora campestris* Engler	Somalia	Stem bark: Used orally to treat hemorrhoids
*Commiphora* cf. *africana* (A.Rich.) Engler	Tanzania	Stem skin, root: oral decoction for fever, cold, stomach disease, colic, swelling, malaria, leprosy, plum poison, poisonous snake bite.
*Commiphora* cf. *boiviana* Engler	Ethiopia	Root and stem skin: oral decoction for prolactin, aphrodisiac, dysentery, gonorrhea.
*Commiphora dalzielii* Hutch	Northern Nigeria	Stem bark: used for anti-inflammatory, analgesic, senile diseases.
*Commiphora erlangeriana*	Somalia, Ethiopia	Resin: Toxic to humans and animals, historically used as curare in Africa.
*Commiphora erythraea*	India, Somalia	Resin: used to protect livestock from ticks and to treat diseases related to inflammation.
*Commiphora gileadensis*	Djibouti, Ethiopia, Kenya, Somalia, Sudan	Tincture of ground balsam bark: used to treat skin diseases The leaves and flowers of the plant: used as analgesic, laxative, and diuretic agents
*Commiphora guidotti*	Somalia	Gum: Oral for stomach disease, diarrhea, maternal displacement of placenta, newborn Robust.
*Commiphora guillauminiperr*	Sudan, Kenya	The plant of Kenyan myrrh.
*Commiphora holtziana*	Eastern Africa	Resin: heal wounds, oral medicines, and perfumery substances, against the cattle tick
*Commiphora habessinica* (O.Berg) Engl.	Uganda	Medicinal plants for veterinary use. Exudate: for scabies.
*Commiphora incisa* Chiov.	India, East Africa, Ethiopia	The plant of Ethiopian medicine myrrh
*Commiphora kataf* (Forssk), Engler	Saudi Arabia	Medicinal plants of the Arabian region. Resin: Used as a substitute for myrrh.
*Commiphora kua* (R.Brown ex Royle) Vollesen	Yemen	Medicinal plants of Yemen. Leaf: Used for cough, bronchitis, disinfection, oral sterilization.
*Commiphora leptophloeos* (Mart.) J.B. Gillett	Brazil	Brazilian medicinal plants. Stem skin: Decoction orally used to treat cough, bronchitis, influenza.
*Commiphora madagascariensis* Jacq.	Madagascar	Root and fruit: Oral infusion for fever, toothache, abdominal pain, menorrhagia.
*Commiphora marlothii* Engler	South Africa	Plant: Burning inhaling smoke to treat epilepsy.
*Commiphora merkeri*	East Africa	Stem bark extracts: anti-cancer, analgesic, antifungal, acaricidal, mosquito larvicidal activities
*Commiphora molmol*	Somalia, Arab region, Ethiopia	The resin produced is called colloidal myrrh
*Commiphora mossambicensis* Oliver	Zambia	Medicinal plants of Zambia. Root: Used to treat infectious diseases and wounds.
*Commiphora mukul* (Hook.ex Stocks) Engler	Somalia, India	Resin is used as a spice and myrrh substitute
*Commiphora multifoliolata* Gilet	Somalia	Medicinal plants of Somalia. Fresh stem bark, gum: Infusion orally used to treat cholera.
*Commiphora opobalsamum* Engler	Egypt, Saudi Arabia	Bark: Exudates from cuts to make myrrh.
*Commiphora parvifolia* Engler	Yemen	Medicinal plants of Yemen. Bark: used for embalming, diarrhea, dysentery, menstruation, uterine stimulation.
*Commphora pendiculata*	Nigeria	Medicinal plants of Nigeria. Stem bark: used as an incense-burning agent.
*Commiphora pilosa* Engler	Tanzania	Medicinal plants of Tanzania. Stem skin, root: boiled after oral treatment of epilepsy.
*Commiphora pterleifolia* Engler	Tanzania	Medicinal plants of Tanzania. Root: The decoction is used orally for the treatment of headache, internal swelling of women, cervical cancer, oral candidiasis, skin fungal infection.
*Commiphora pyracanthoides* Engler	East Africa	East African myrrh source
*Commiphora resiniflua* Martelli	Ethiopia	Medicinal plants of Ethiopia. Resin: Used for strengthening liver function, repelling worms (tapeworms) and skin damage
*Commiphora rostrota*	Arab region	Plant of origin for the Arabian and Ethiopian medicine myrrh. Stem skin: liniment oral treatment for sore throat, cough, sore throat, eye disease.
*Commiphora wighti* (Arn.) Bhandari	India, Pakistan	Traditional medicinal plants of Pakistan. Resin: topically wash the parasitic rule of law head.
*Commiphora zimmermanii* Engler	Tanzania	Medicinal plants of Tanzania. Branch, leaf, stem skin: decoction or infusion oral for fever, toothache, stomach pain, abdominal pain, constipation, menorrhagia, postpartum bleeding, snake bite.

Table cited in full in A Quick-Consultative Dictionary of World Medicinal Plants [13].

**Table 2:** Classic Chinese herbal preparations.

Name	Ingredients	Actions	Indications
Qili Powder	Draconis Sanguis; Olibanum (processed); Myrrha (processed); Carthami flcis; Catechu; Borneolum Syntheticum; Moschus artifactus; Cinnabaris.	To resolve stasis, disperse swelling, relieve pain, and stop bleeding.	Traumatic injuries, blood stasis pain, and transient bleeding.
Xihuang Pills	Bovis Calculus or Bovis Calculus Sativus; Moschus or Moschus Artifactus; Olibanum (processed with vinegar); Myrrha (processed with vinegar).	To clear heat, remove toxins, disperse swelling, and dissipate cold.	Abscesses, cellulitis, deep-rooted boil toxin, scrofula, deep multiple abscesses, and tumor swelling due to exuberant heat toxins.
Niuhuang Huadu Tablets	Arisaematis Rhizoma Preparatum; Forsythiae Fructus; Lonicerae Japonicae Flos; Angelicae Dahuricae Radix; Glycyrrhizae Radix et Rhizoma; Olibanum; Myrrha; Bovis Calculus Artifactus.	To remove toxins, alleviate swelling, dissipate cold, and relieve pain.	Swelling, reddening, and pain in skin infections or acute mastitis.
Xiaohuoluo Wan	Arisaema cum Bile; Aconiti Radix Cocta; Aconiti Kusnezoffii Radix Cocta; Pheretima; Olibanum (processed); Myrrha (processed).	To dispel wind, dissipate cold, resolve stasis, eliminate dampness, activate blood, and relieve pain.	Disorders due to wind–cold–dampness obstruction and phlegm stasis obstructing the collaterals, manifested as pain in the joints and limbs, either cold pain, stabbing pain, or pain worsening at night, with inhibited bending and stretching, numbness, and convulsions of the joints.
Gutongling Liquid	Aconiti Brachypodi Radix; ZingiberisRhizoma; Dracaenae Resinalg; Olibanum; Myrrha; Borneolum Syntheticum.	To warm the meridians, dissipate cold, dispel wind, activate blood, unblock the collaterals, and relieve pain.	Lumbar and cervical vertebrae osteoproliferation, osteoarthritis, shoulder periarthritis, and rheumatic arthritis.

**Table 3:** Terpenoids.

Compounds	Species	Type	Ref
α-Pinene	*C.quadricincta*, *C. sphaerocarpa C. holtziana C. kataf*	monoterpenoids	[68,69]
camphene	*C.africana*, *C. campesiris*, *C. ogadensis*	monoterpenoids	[70]
β-Pinene	*C.africana*, *C. campesiris*, *C. ogadensis*	monoterpenoids	[70]
7-Methyl-3-methylene-1,6-octadiene	*C. sphaerocarpa*, *C. africana*, *C. ogadensis*	monoterpenoids	[70]
limonene	*C. africana*, *C. campesiris*, *C. ogadensis*, *C. terebinthina*, *C. cyclophylla*	monoterpenoids	[71,72]
3,7-dimethylocta-1,3,7-triene	*C. wildii*	monoterpenoids	[73,74]
borneol	*C. ornifolia*, *C. parvifolia.*	monoterpenoids	[71]
β-elemene	*C. myrrha*, *C. sphaerocarpa*, *C. holtziana*, *C. kataf*	monoterpenoids	[69]
2-methoxyfuranodiene	*C. myrrha*, *C. molmol*, *C. erythraea*	Germacrane	[75,76,77,78,79]
2-acetoxyfuranodiene	*C. myrrha*, *C. molmol*, *C. erythraea*	Germacrane	[75,79,80]
furanodiene	*C. myrrha*, *C. guidotti*, ResinaCommiphora	Germacrane	[69,70,78,79,81]
4,5-dihydrofuranodiene-6-one	*C. molmol*	Germacrane	[80]
1,2-epoxyfurano-l0(15)-germamen-6-one	*C. myrrha*, *C. holtziana*, *C. opobalsamum*, Resina Commiphora	Germacrane	[57,76,78,81,82]
(1E)-8,12epoxygermacra-1,7,10,11-tetraen-6-one	*C. sphaerocarpa*	Germacrane	[69]
(1E)-3-methoxy-8,12-epoxygermacra-1,7,10,11-tetraen-6 one	*C. opobalsamum*, *C. erythraea*, *C. sphaerocarpa*, *C. holtziana*	Germacrane	[55,57,69,76]
2-methoxy-5-acetoxy-fruranogermacr-1(10)-en-6-one	*C. myrrha*, *C. opobalsamum*	Germacrane	[63,76,83,84,85]
[1(10)E,2R^*^,4R^*^]-2-methoxy-8,12-epoxygermacra-1(10),7,11-trien-6-one	*C. myrrha*, *C. opobalsamum*, *C. sphaerocarpa*, *C. erythraea*, *C. holtziana*, Resina Commiphora	Germacrane	[55,69,76,78,82,83,85]
epicurzerenone	*C. myrrha*	Germacrane	[77]
furanodieneone	*C. myrrha*, *C. molmol*, *C. guidotti*, *C. erythraea*, *C. sphaerocarpa*, Resina Commiphora	Germacrane	[55,78,79,81,82]
2-acetyloxyglechomanolide	Resina Commiphora	Germacrane	[78]
8-epi-2-acetyloxyglechomanolide	Resina Commiphora	Germacrane	[78]
rel-2R-methyl-5S-acetoxy-4R-furanogermacr-1(10)Z-en-6 one	*C. myrrha*, Resina Commiphora	Germacrane	[78,82]
2-hydroxy-11,12-dihydrofuranodiene	*C. molmol*	Germacrane	[79]
2-hydroxy-furanodiene	*C. molmol*	Germacrane	[79]
rel-(1S,2S,3R,4S)-1,2-epoxy-3-methoxyfuranogermacr-10 (15)-en-6-one	*C. opobalsamum*	Germacrane	[83]
2α-methoxy-8α-hydroxy-6-oxogermacra-1(10),7(11)-dien 12,8-olide	*C. opobalsamum*	Germacrane	[56,83]
2α-methoxy-6-oxogermacra-1(10),7(11)-dien-8,12-olide	*C. opobalsamum*	Germacrane	[57]
myrrhterpenoid B	*C. myrrha*	Germacrane	[84]
myrrhterpenoid C	*C. myrrha*	Germacrane	[84]
myrrhterpenoid D	*C. myrrha*	Germacrane	[84]
myrrhterpenoid E	*C. myrrha*	Germacrane	[84]
myrrhterpenoid F	*C. myrrha*	Germacrane	[84]
germacrone	*C. myrrha*, *C. holtziana*	Germacrane	[86]
1β,8β-epoxy-2α-methoxy-6-oxogermacra-9(10),7(11)-dien 8,12-olide	*C. opobalsamum*	Germacrane	[56]
1β,8β-epoxy-2α-methoxy-12α-hydroxy-6-oxogermacra-9 (10),7(11)-dien-8,12-olide	*C. opobalsamum*	Germacrane	[56]
1β,8β-epoxy-2α-methoxy-12β-hydroxy-6-oxogermacra-9 (10),7(11)-dien-8,12-olide	*C. opobalsamum*	Germacrane	[56]
eudesm-4(15)-ene-1β,6α-diol	*C. myrrha*, *C. opobalsamum*	Eudesmane	[56,77]
isohydroxylindestrenolide	*C. myrrha*, ResinaCommiphora	Eudesmane	[78,87]
hydroxylindestrenolide	*C. myrrha*, ResinaCommiphora	Eudesmane	[79,87]
5-αH,8-βH-eudesma-1,3,7(11)-trien-8,12-olide	*C. molmol*	Eudesmane	[79]
furanoeudesma-1,3-diene	*C. myrrha*, *C. molmol*	Eudesmane	[1,79,81]
furanoeudesma-1,4-diene-6-one	*C. molmol*	Eudesmane	[1]
myrrhterpenoid A	*C. myrrha*	Eudesmane	[84]
chlorantene C	*C. myrrha*	Eudesmane	[84]
chlomultin B	*C. myrrha*	Eudesmane	[84]
eudesmane-1β,5α,11-triol	*C. opobalsamum*	Eudesmane	[85]
β-selinene	*C. holtziana*	Eudesmane	[86]
11-hydroxy-4α-methoxy-selinane	*C. opobalsamum*	Eudesmane	[56]
1β,4β-epoxy-eudesmane-11-ol	*C. opobalsamum*	Eudesmane	[56]
9-nor-9,10-seco-isolindestrenolide	*C. myrrha*	Eudesmane	[87]
9,10-seco-isohydroxylindestrenolide	*C. myrrha*	Eudesmane	[87]
lindestrenolide	*C. myrrha*	Eudesmane	[87]
atractylenolide	*C. myrrha*	Eudesmane	[87]
4β-hydroxy-8,12-epoxyeudesma-7,11-diene-1,6-dione	*C. myrrha*	Eudesmane	[87]
lindestrene	*C. myrrha*, *C. molmol*	Eudesmane	[80,81]
commiphoraneI	Resina Commiphora	Eudesmane	[88]
commiphorane E1	Resina Commiphora	Eudesmane	[61]
commiphorane E2	Resina Commiphora	Eudesmane	[61]
commiphorane E3	Resina Commiphora	Eudesmane	[61]
curcolonol	Resina Commiphora	Eudesmane	[61]
myrrhterpenoid M	*C. myrrha*	Eudesmane	[89]
myrrhterpenoid N	*C. myrrha*	Eudesmane	[89]
myrrhanolide A	*C. myrrha*	Eudesmane	[90]
2-methoxyfuranoguaia-9-ene-8-one	*C. molmol*	Guaiane	[80]
(1R,2R,4S)-1,2-epoxyfuranogermacr-10(15)-en-6-one	Resina Commiphora	Guaiane	[78]
alismol	*C. myrrha*, *C. opobalsamum*	Guaiane	[83]
6α,7α-epoxy-1β-guai-10(14)-en-4α-ol	*C. opobalsamum*	Guaiane	[57]
5β-10α-hydroxy-2α-methoxy-6-oxoguaia-7(11),8-dien-8,12-olide	*C. opobalsamum*	Guaiane	[57]
(1R,4S,5R)-guaia-6,10(14)-diene	*C. opobalsamum*	Guaiane	[57]
myrrhterpenoid O	Resina Commiphora	Guaiane	[78]
myrrhterpenoid G	*C. myrrha*	Guaiane	[84]
myrrhterpenoid H	*C. myrrha*	Guaiane	[84]
myrrhterpenoid I	*C. myrrha*	Guaiane	[84]
myrrhterpenoid K	*C. myrrha*	Guaiane	[89]
myrrhterpenoid L	*C. myrrha*	Guaiane	[89]
guaia-6α,7α-epoxy-4α,10α-diol	*C. opobalsamum*	Guaiane	[85]
guaia-4β,7β,10α-trihydroxy-5-ene	*C. opobalsamum*	Guaiane	[56]
myrrhanoperoxide	*C. myrrha*	Guaiane	[87]
rel-(+)-(1S,4R,7S)-11-acetyl-guai-10(14)-en-4,11-ol	*C. myrrha*	Guaiane	[87]
rel-(+)-(4R,5R,7S)-11-acetyl-guai-1(10)-en-4,11-ol	*C. myrrha*	Guaiane	[87]
commiphorane J	Resina Commiphora	Guaiane	[88]
guai-1(10),5,7(11),8-tetradien-12,8-olide	*C. myrrha*	Guaiane	[91]
commiphoranoid A	Resina Commiphora	Guaiane	[92]
commiphoranoid B	Resina Commiphora	Guaiane	[92]
commiphoranoid C	Resina Commiphora	Guaiane	[92]
dihydropyrocurzerenone	*C. myrrha*, *C. sphaerocarpa*, *C. opobalsamum*	Cadinane	[69,85]
τ-cadinol	*C. myrrha*, *C. molmol*, *C. guidottii*, *C. kua*	Cadinane	[60,77,93,94]
3α-hydroxy-τ-cadinol	*C. myrrha*, *C. guidottii*	Cadinane	[77]
myrrhone	*C. myrrha*, *C. opobalsamum*, *C. erythraea*, Resina Commiphora	Cadinane	[55,58,77,78,83,85,95]
9-methoxymyrrhone	*C. opobalsamum*	Cadinane	[83]
agarsenone	*C. opobalsamum*, *C. erythraea*	Cadinane	[83]
myrrhanolide B	*C. myrrha*, *C. opobalsamum*, Resina Commiphora	Cadinane	[83,90,95]
furanocadina-1(10),6,8-triene-4-ol	*C. opobalsamum*	Cadinane	[57]
myrrhterpenoid J	*C. myrrha*	Cadinane	[84]
commipholinone	*C. myrrha*, *C. opobalsamum*, Resina Commiphora	Cadinane	[56,58,95]
commiterpene D	*C. myrrha*	Cadinane	[87]
(11β)-8,11-dihydroxy-cadina-6,8,10-trien-12-oicacid-γ lactone	Resina Commiphora	Cadinane	[88]
commiphorane H	Resina Commiphora	Cadinane	[88]
(+)-myrrhalactone A	*C. myrrha*	Cadinane	[96]
(–)-myrrhalactone A	*C. myrrha*	Cadinane	[96]
(±)-commyrrin A	*C. myrrha*	Cadinane	[91]
(±)-commyrrin B	*C. myrrha*	Cadinane	[91]
commiphoin A	*C. myrrha*	Cadinane	[56]
commiphoin B	*C. myrrha*	Cadinane	[56]
commiphoin C	*C. myrrha*	Cadinane	[56]
commiterpene A	*C. myrrha*	Cadinane	[56]
commiphorene A	Resina Commiphora	Cadinane	[95]
commiphorene B	Resina Commiphora	Cadinane	[95]
myrrhanolide C	*C. myrrha*, Resina Commiphora	Cadinane	[90,95]
myrracalamene A	*C. myrrha*	Cadinane	[97]
myrracalamene B	*C. myrrha*	Cadinane	[97]
myrracalamene C	*C. myrrha*	Cadinane	[97]
myrracadinol B	*C. myrrha*	Cadinane	[97]
myrracadinol C	*C. myrrha*	Cadinane	[97]
myrracadinol A	*C. myrrha*	Cadinane	[97]
8-hydroxy-12-norcardina-4,6,8,10-tetraen-11-one	Resina Commiphora	Cadinane	[98]
commiterpene A	*C. myrrha*	Cadinane	[99]
commiterpene B	*C. myrrha*	Cadinane	[99]
commiterpene C	*C. myrrha*	Cadinane	[99]
curzerenone	*C. myrrha*, *C. sphaerocarpa*, *C. erythraea*, *C.* *Opobalsamum*, Resina Commiphora	Elemane	[55,69,78,82,85]
2-methoxyisogermafurenolide	Resina Commiphora	Elemane	[78]
8-epi-2-methoxyisogermafurenolide	*C. myrrha*, Resina Commiphora	Elemane	[78,87]
2-methoxy isofuranogermacrene	*C. myrrha*, *C. molmol*, *C. erythraea*, Resina Commiphora	Elemane	[78,79]
β-elemene	*C. myrrha*, *C. molmol*, Resina Commiphora	Elemane	[78,86,94]
elemyl acetate	Resina Commiphora	Elemane	[78]
8-hydroxyisogermafurenolide	Resina Commiphora	Elemane	[78]
γ-elemanel actone	*C. molmol*	Elemane	[79]
isofuranogermacrene	*C. myrrha*	Elemane	[81,100]
elemol	*C. holtziana*	Elemane	[86]
δ-elemene	*C. holtziana*	Elemane	[86]
γ-elemene	*C. myrrha*, *C. holtziana*	Elemane	[86]
isogermafurenolide	*C. myrrha*	Elemane	[87]
hydroxyisogermafurenolide	*C. myrrha*	Elemane	[87]
methoxyisogermafurenolide	*C. myrrha*	Elemane	[87]
α-bisabolene	*C. guidotti*	Others	[70]
β-bisabolene	*C. guidotti*	Others	[70,74]
α-santalene	*C. guidotti*	Others	[70]
α-cubebene	*C. myrrha*	Others	[86]
β-bourhonene	*C. holtziana*	Others	[86]
commipholactam A	Resina Commiphora	Others	[88]
commiphorane C	Resina Commiphora	Others	[64]
commiphorane D	Resina Commiphora	Others	[64]
2-methyl-5-(5′-hydroxy-1′,5′-dimethyl-3′-hexenyl)phenol	*C. kua*	Others	[101]
6-hydroxy-2-methyl-5-(5′-hydroxy-1′(R),5′-dimethylhex 3′-enyl)-phenol	*C. kua*	Others	[65]
2-methyl-5-[4′(S)-hydroxy-1′(R),5′-dimethylhex-5′ enyl]-phenol	*C. kua*	Others	[65]
7-oxo-13α-hydroxyabiet-8(14)-en-18-oic acid	Resina Commiphora	Abietane	[61]
7-oxo-13β-hydroxyabiet-8(14)-en-18-oic acid	Resina Commiphora	Abietane	[61]
7-oxo-13α-methoxyabiet-8(14)-en-18-oic acid	Resina Commiphora	Abietane	[61]
7-oxo-13β-methoxyabiet-8(14)-en-18-oic acid	Resina Commiphora	Abietane	[61]
Dehydroabietic acid	*C. myrrha*, Resina Commiphora	Abietane	[61,63]
7-oxocallitrisic acid	*C. myrrha*, Resina Commiphora	Abietane	[61,98]
abieta-8,11,13,15-tetraen-18-oic acid	Resina Commiphora	Abietane	[61]
19-norabieta-5,8,11,13-tetraen-7-one	Resina Commiphora	Abietane	[61]
abietic acid	*C. myrrha*	Abietane	[63]
commiphoranesK_1_	Resina Commiphora	Abietane	[88]
commiphoranesK_2_	Resina Commiphora	Abietane	[88]
nepetaefolinF	Resina Commiphora	Abietane	[98]
3β-hydroxy-dehydroabietic acid	Resina Commiphora	Abietane	[88]
(1E,5E,9E)-1,5,9-trimethyl-12-(1-methylethenyl)cyclotetradeca-1,5,9-triene	*C. mukul*	Cembrane	[62]
(2E,6E,10E)-3,7,11-trimethyl-14-(1-methylethenyl)cyclotetradeca-2,6,10-trien-1-ol	*C. mukul*	Cembrane	[62]
(1E,3E,6E,10E)-3,7,11-trimethyl-14-(1-methylethyl)cyclotetradeca-1,3,6,10-tetraene	*C. mukul*	Cembrane	[62]
(2E,6E,10E)-3,7,11-trimethyl-14-(1-methylethyl)cyclotetradeca-2,6,10-trien-1-ol	*C. mukul*	Cembrane	[62]
(1E,4E,8E)-4,8,14-trimethyl-11-(1-methylethyl)-14-methoxycyclotetradeca-1,4,8-triene	*C. mukul*	Cembrane	[62]
(2E,12E)-2,7,13-trimethyl-9-(1-methylethyl)-15-oxabicyclo [12.1.0]pentadeca-2,12-dien-7-ol	*C. mukul*	Cembrane	[62]
cembrene	*C. mukul*	Cembrane	[102]
cembrene A	*C. mukul*	Cembrane	[102]
cembrenol	*C. mukul*	Cembrane	[102]
mukulol	*C. mukul*	Cembrane	[102,103]
isocembrol	*C. mukul*	Cembrane	[103]
4-epiisocembrol	*C. mukul*	Cembrane	[103]
commiphorane A	Resina Commiphora	6/6/6/6	[64]
commiphorane B	Resina Commiphora	6/6/6/6	[64]
pimaricacid	Resina Commiphora	Pimarane	[61]
pimarol	Resina Commiphora	Pimarane	[61]
sandaracopimaric acid	*C. myrrha*	Pimarane	[63]
commiphorane F	Resina Commiphora	Podocarpinene	[61]
8(14)-podocarpen-13-on-18-oic acid	Resina Commiphora	Podocarpinene	[61]
(4Z,6E)-4,7,12,15,15-pentamethylbicyclo [9.3.1]pentadeca-4,6-dien-12-ol.	*C. mukul*	-	[62]
verticillol	*C. mukul*	-	[102]
commiphoraneG1	Resina Commiphora	Dammarane	[61]
(20S)-3β-acetoxy-12β,16β-trihydroxydammar-24-ene	*C. confusa*	Dammarane	[104]
(20S)-12β,16β-trihydroxydammar-24-ene-3β-O-glucopyranoside	*C. confusa*	Dammarane	[104]
(20R)-3β-ace-toxy-16β-dihydroxydammar-24-ene	*C. confusa*	Dammarane	[104]
3β-hydroxydammar-24-ene	*C. confusa*	Dammarane	[104]
3β-acetoxydammar-24-ene	*C. confusa*	Dammarane	[104]
(20R)3β-acetoxy-16β-hydroxydammar-24-ene	*C. confusa*	Dammarane	[104]
(20R)-3β,16β-trihydroxydammar-24-ene	*C. confusa*	Dammarane	[104]
(20S)-3β-acetoxy-12β,16β,25-tetrahydroxydammar-23-ene	*C. confusa*	Dammarane	[104]
(20S)-3β,12β,16β,25-pentahdroxydammar-23-ene	*C. confusa*	Dammarane	[104]
3β,16β,20(S),25-tetrahydroxydammar-23-ene	*C. kua*	Dammarane	[65]
3β-acetoxy-16β,20(S),25-trihydroxydammar-23-ene	*C. kua*	Dammarane	[65]
3β,16β,20(R)-trihydroxydammar-24-ene	*C. kua*	Dammarane	[65]
3β-acetoxy-16β,20(R)-dihydroxydammar-24-ene	*C. kua*	Dammarane	[65]
(3R,20S)-3,20-dihydroxydammar-24-ene	*C. confusa*	Dammarane	[67]
α-amyrin	*C. confusa*	Dammarane	[67]
(3R,20S)-3-acetoxy-20-hydroxydammar-24-ene	*C. confusa*	Dammarane	[67]
cabraleadiol3-acetate	*C. confusa*	Dammarane	[67]
rel-20S-hydroxy-dammar-24-en-3,16-dione	Resina Commiphora	Dammarane	[78]
rel-(16S,20S)-dihydroxydammar-24-en-3-one	Resina Commiphora	Dammarane	[78]
(16S,20R)-dihydroxydammar-24-en-3-one	*C. kua*	Dammarane	[60]
15α-hydroxymansumbinone	*C. kua*	Dammarane	[60]
28-acetoxy-15α-hydroxymansumbinone	*C. kua*	Dammarane	[60]
mansumbinone	*C. molmol*, *C. kua*, Resina Commiphora	Dammarane	[60,78,94]
mansumbinol	*C. molmol*	Dammarane	[60,78]
Mansumbinol epoxide	Resina Commiphora	Dammarane	[78]
mansumbin-13(17)-en-3,16-dione	Resina Commiphora	Dammarane	[78]
3,4-seco-mansumbinoic acid	*C. molmol*, Resina Commiphora	Dammarane	[60,78]
3-oxo-commiphoraneG_2_	Resina Commiphora	Dammarane	[98]
commiphoraneG_2_	Resina Commiphora	Dammarane	[61]
epimansumbinol	*C. mukul*	Dammarane	[103]
myrrhasin	*C. myrrha*	Dammarane	[90]
cycloartan-24-ene-1α,2α,3β-triol	*C. myrrha*, *C. opobalsamum*, Resina Commiphora	Cycloartane	[78,85,86,105,106]
cycloartan-24-ene-1α,2α,3α-triol	*C. opobalsamum*	Cycloartane	[106]
3β-acetoxycycloartan-24-ene-1α,2α-diol	*C. opobalsamum*	Cycloartane	[106]
1α-acetoxycycloartan-24-ene-2α,3β-diol	*C. opobalsamum*	Cycloartane	[56,106]
3β-isovaleroyloxycycloartan-24-ene-1α,2α-diol	*C. opobalsamum*	Cycloartane	[56,106]
cycloartan-24-ene-1α,3β-diol	*C. opobalsamum*	Cycloartane	[106]
cycloartan-24-ene-1S,3R-diol	*C. opobalsamum*	Cycloartane	[56]
cycloartan-24-ene-1α,2α,3β-triol	*C. opobalsamum*	Cycloartane	[106]
cycloartane-1α,2α,3β,25-tetraol	*C. myrrha*	Cycloartane	[63]
cycloartan-23E-ene-1α,2α,3β,25-tetrol	*C. opobalsamum*	Cycloartane	[106]
24R,25-epoxycycloartane-1α,2α,3β-triol	*C. opobalsamum*	Cycloartane	[106]
24S,25-epoxycycloartane-1α,2α,3β-triol	*C. opobalsamum*	Cycloartane	[106]
cycloartan-23-ene-1S′,3R′,25-triol	*C. opobalsamum*	Cycloartane	[56]
cycloartane-24-en-1α,2α,3β-triol-1,2-acetonide	*C. opobalsamum*	Cycloartane	[56]
1α-acetoxy-9,19-cyclolanost-24-en-3β-ol	*C. kua*, *C. myrrha*, *C. incisa*	Cycloartane	[66]
myrrhanol B	*C. mukul*	Polypodane	[103]
myrrhanone B	*C. mukul*	Polypodane	[103]
myrrhanone Aacetate	*C. mukul*	Polypodane	[103]
(13E,17E,21E)-polypodo-13,17,21-triene-3,8-dio	*C. mukul*, *C. wightii*	Polypodane	[62,107]
myrrhanol A	*C. mukul*, *C. wightii*	Polypodane	[103,108,109]
myrrhanone A	*C. mukul*, *C. wightii*	Polypodane	[103,108,109]
(13E,17E,21E)-8-hydroxypolypodo-13,17,21-trien-3-one	*C. mukul*, *C. wightii*	Polypodane	[62,107]
7-oxo-ganodericacidZ	Resina Commiphora	Lanostane	[98]
29-norlanost-8,24-dien-1α,2α,3β-triol	*C. myrrha*, *C. kua*, *C. incisa*	Lanostane	[66]
2α,3β-diacetoxy-29-norlanost-8,24-dien-lα-ol	*C. incisa*	Lanostane	[66]
lα,2α,3β-triacetoxy-29-norlanost-8,24-diene	*C. incisa*	Lanostane	[66]
3β-acetoxy-24-methyl-29-norlanost-8,25-diene	*C. incisa*	Lanostane	[66]
commiphoraneG_3_	Resina Commiphora	Ursane	[61]
28-nor-urs-12-ene-3β,17β-diol	Resina Commiphora	Ursane	[61]
3,22-dioxo-20-taraxastene	Resina Commiphora	Taraxastane	[61]
β-amyrin	*C. confusa*	Oleanane	[65]

**Table 4:** Lignans.

Compounds	Species	Ref
diasesartemin	*C. wightii*	[107,112]
sesamin	*C. wightii*	[107]
5,5′-tetrahydro-1H,3H-furo[3,4-c]furan-1,4-diylbis[7-(methoxy)-1,3-benzodioxole]	*C. wightii*	[62]
epi-mukulin	*C. wightii*	[112]
(+)-epi-magnolin	*C. wightii*	[112]
(+)-diayangambin	*C. wightii*	[112]
erlangerin A	*C. erlangeriana*	[69]
erlangerin B	*C. erlangeriana*	[69]
erlangerin C	*C. erlangeriana*	[69]
erlangerin D	*C. erlangeriana*	[69]

**Table 5:** Steroids.

Compounds	Species	Ref
E-guggulusterone	*C. mukul*, *C. wightii*	[62,103,107,109,112,115]
Z-guggulusterone	*C. mukul*, *C. wightii*	[62,103,107,109,112,115]
pregn-4-ene-3,16-dione	*C. mukul*	[62]
progesterone	*C. mukul*	[103]
16β-acetyloxy-pregn-4,17(20)-trans-dien-3-one	*C. mukul*	[103]
3α-acetyloxy-5α-pregnan-16-one	*C. mukul*	[103]
20R,22R-dihydroxycholest-4-en-3-one	*C. mukul*	[103]
guggulsterol I	*C. mukul*, *C. wightii*	[103,109,112,115]
guggulsterol II	*C. mukul*	[115]
guggulsterol III	*C. mukul*, *C. wightii*	[107,115]
guggulsterol IV	*C. mukul*	[113]
8β-hydroxypregnene-4,6-diene-3,20-dione	*C. wightii*	[107]
20-acetyloxy-4-pregnene-3,16-dione	*C. mukul*, *C. wightii*	[62,103,107]
20α-hydroxy-4-pregnen-3-one	*C. mukul*	[115]
20β-hydroxy-4-pregnen-3-one	*C. mukul*	[115]
16β-hydroxy-4,17(20)Z-pregnadien-3-one	*C. mukul*	[115]
(Z)Δ^1,2^dehydroguggulsterone	*C. wightii*	[112]
Δ^6,7^dehydro-20-hydroxygugglsterone	*C. wightii*	[112]
β-sitostenone	*C. myrrha*	[91]
β-sitosterol	*C. myrrha*	[91,104]
Guggulsterone-M	*C. wightii*	[109]
Dehydroguggulsterone-M	*C. wightii*	[109]
Guggulsterol-Y	*C. wightii*	[109]
β-epimer	*C. myrrha*	[115]
16α-hydroxy-4-pregnen-3-one	*C. mukul*	[115]

**Table 6:** Comparison of pharmacopoeias.

Inspection	ChP2020	EP11.0	BP2024	IP2022	USP2024-NF42	
Name	Myrrha	Myrrha	Myrrh	Guggul Resin	Myrrh	Guggul
*Commiphora* Type	*Commiphora myrrha* Engl. *Commiphora molmol* Engl.	*Commiphora myrrha* (Nees) Engl. (syn.*Commiphora molmol* (Engl.) Engl. ex Tschirch) and/or other species of *Commiphora*.	*Commiphora myrrha* (Nees) Engl. (syn. *Commiphora molmol* (Engl.) Engl. ex Tschirch) and/or other species of *Commiphora*.	*Commiphora wightii* (Arnott) Bhandari (*Commiphora mukul* (Arn.) Bhandari, *balsamodendron mukul* Hook. ex. Stocks) (Fam. Burseraceae)	*Commiphora molmol* Engler and other related species of *Commiphora* other than *Commiphora mukul* (Fam. Burseraceae)	*Commiphora wightii* (Arn.) Bhandari, also known as *Commiphora mukul* (Hook. ex. Stocks) Engl. Or *Balsamodendrum mukul* (Hook.) (Fam. Burseraceae).
Content determination	Volatile oil: natural myrrh not less than 4% (mL/g); colloidal myrrh not less than 2% (mL/g)	Matter insoluble in ethanol: max 75%	Matter insoluble in ethanol: max 75%	Ethyl acetate-soluble extractive: not less than 25.0% Ethanol-soluble extractive: not less than 35.0%	Alcohol-Soluble Extractives: 40–70% Water-Soluble Extractives: not less than 50% Volatile Oil Determination: not less than 6.0%	Alcohol-Soluble Extractives: not less than 33%
Identification	Chemical method: Thin-layer chromatography	Microscopic examination; Thin-layer chromatography (*C. mukul*)	Microscopic examination; Thin-layer chromatography (*C. mukul*)	Chemical method; Thin-layer chromatography	Chemical method: Thin-layer chromatography	Thin-layer chromatography; HPLC Analysis
Foreign matter	Natural myrrh: Max 10.0% Colloidal myrrh: Max 15.0%	Matter insoluble in ethanol: max 75%	Matter insoluble in ethanol: max 75%	-	Organic Matter: Max 2%; Pesticide Residue Analysis: Meets the requirements	Limits of Elemental Impurities: Meets the requirements Pesticide Residue Analysis: Meets the requirements
Loss on drying	-	Max 15%	Max 15%	-	Max 15%	-
Total ash	Max 15.0%	Max 7%	Max 7%	Max 10%	Max 10%	-

## Data Availability

Data will be made available on request.

## Supplementary Materials

The following supporting information can be downloaded at: https://www.mdpi.com/article/10.3390/ph17111524/s1, all images are named using the chemical names of the structural formulas. The structures of all the chemical components listed in this paper will be supplemented in the form of compressed packages.

## Glossary

AChE	Acetylcholinesterase	LLC	Lewis Lung Carcinoma
AD	Anti-Alzheimer’s Disease	LPS	Lipopolysaccharide
AKT	Protein Kinase B	MDA	Malondialdehyde
ARE	Antioxidant Response Elements	MAPK	Mitogen-Activated Protein Kinase
ARP	Atherogenic Risk Predictor	MIA	Monosodium Iodoacetate
Bak	Bcl-2 Homologous Antagonist/Killer	NF-κB	Nuclear Factor Kappa-B
Bax	Bcl-2 Associated X Protein	Nrf2	Nuclear Factor-Erythroid 2-Related Factor 2
Bcl-2	B-cell Lymphoma-2	NSCLC	Non-Small Cell Lung Cancer
CCl4	Carbon Tetrachloride	OA	Osteoarthritis
CE	Critically Endangered	p38	Mitogen-Activated Protein Kinase p38
ChP	Chinese Pharmacopoeia	PB	Phenobarbital
COX-1	Cyclooxygenase-1	PD-1	Programmed Cell Death Protein 1
COX-2	Cyclooxygenase-2	PD-L1	Programmed Death-Ligand 1
CPT1	Carnitine Palmitoyltransferase I	PGs	Prostaglandins
DD	Data Deficient	PGE_2_	Prostaglandin E2
DEN	Diethylnitrosamine	PLA2	Phospholipase A2
DOX	Doxorubicin	PPARα	Peroxisome Proliferator-Activated Receptor Alpha
EP	European Pharmacopoeia	PPARγ	Peroxisome Proliferator-Activated Receptor Gamma
ERK	Extracellular Signal-Regulated Protein Kinase	PTEN	Phosphatase and Tensin Homolog
FXR	Farnesoid X Receptor	ROI	Reactive Oxygen Intermediates
GC-MS	Gas Chromatography-Mass Spectrometry	ROS	Reactive Oxygen Species
GPx	Glutathione Peroxidase	SOD	Superoxide Dismutase
GSH	Glutathione	ST	Smokeless Tobacco
HMEEC	Human Middle Ear Epithelial Cells	STAT	Signal Transducer and Activator of Transcription
HNSCC	Head and Neck Squamous Cell Carcinoma	STZ	Streptozotocin
HO-1	Heme Oxygenase-1	TCF-4	Transcription Factor 4
HPLC	High-performance liquid chromatography	TF	Tissue Factor
IC_50_	Half Maximal Inhibitory Concentration	TGF-β	Transforming Growth Factor Beta
IFN-γ	Interferon-gamma	TLC	Thin Layer Chromatography
IL-1β	Interleukin-1β	TMA	Trimethylamine
IL-6	Interleukin-6	TMAO	Trimethylamine N-Oxide
IL-10	Interleukin-10	TNF-α	Tumor Necrosis Factor Alpha
IL-17	Interleukin-17	USP	United States Pharmacopeia
IL-23	Interleukin-23	VEGF	Vascular Endothelial Growth Factor
iNOS	Inducible Nitric Oxide Synthase	VEGF-R2	VEGF Receptor 2
IP	Indian Pharmacopoeia	VLDL	Very Low-Density Lipoprotein
JNK	c-Jun N-terminal Kinase	α-SMA	Alpha-Smooth Muscle Actin
LDL	Low-Density Lipoprotein	β-Catenin	Beta-Catenin
