# Supplementary figures and images for: The Genus Commiphora: An Overview of Its Traditional Uses, Phytochemistry, Pharmacology, and Quality Control (part 2 of 2)
Source: Pharmaceuticals (Basel). 2024 Nov 12;17(11):1524. doi: 10.3390/ph17111524 (PMC11597752; doi:10.3390/ph17111524)

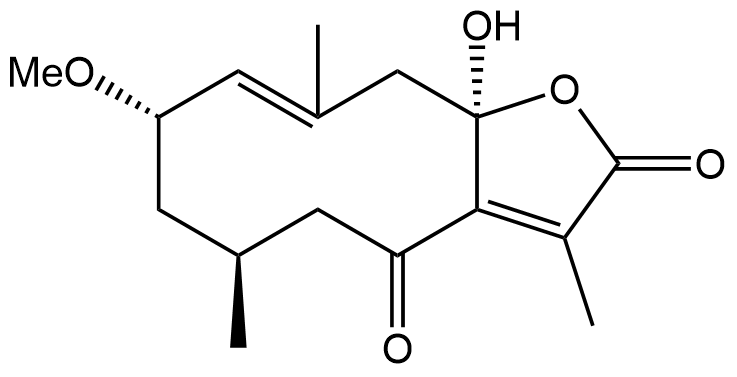

Supplement: Supplementary file 1 [file pharmaceuticals-17-01524-s001.zip › Sesquiterpene chemical structure/2α-methoxy-8α-hydroxy-6-oxogermacra-1(10),7(11)-dien.png]

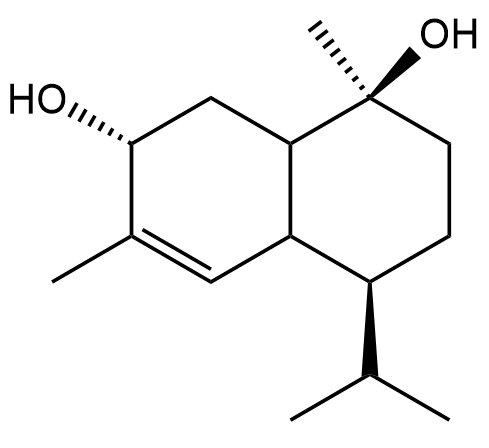

Supplement: Supplementary file 1 [file pharmaceuticals-17-01524-s001.zip › Sesquiterpene chemical structure/3α-hydroxy-τ-cadinol.png]

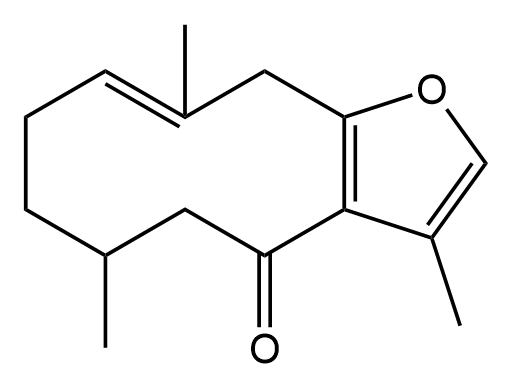

Supplement: Supplementary file 1 [file pharmaceuticals-17-01524-s001.zip › Sesquiterpene chemical structure/4,5-dihydrofuranodiene-6-one.png]

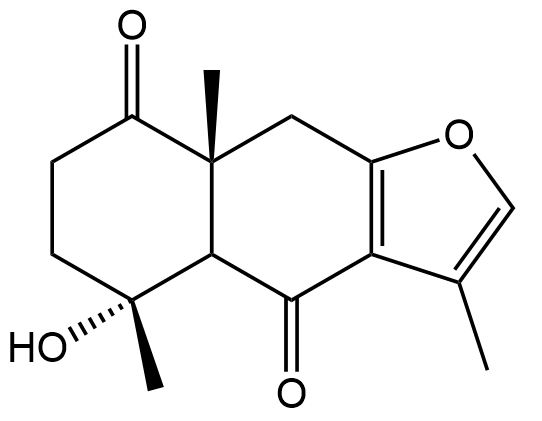

Supplement: Supplementary file 1 [file pharmaceuticals-17-01524-s001.zip › Sesquiterpene chemical structure/4β-hydroxy-8,12-epoxyeudesma-7,11-diene-1,6-dione.png]

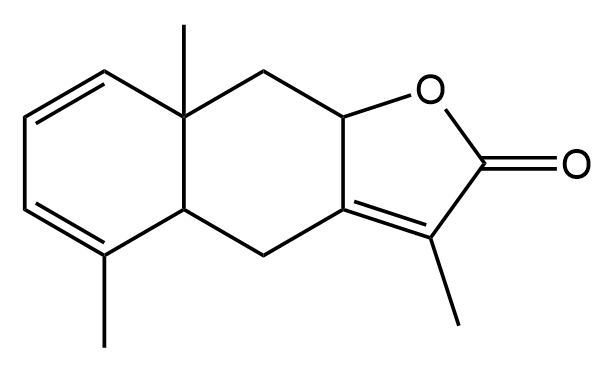

Supplement: Supplementary file 1 [file pharmaceuticals-17-01524-s001.zip › Sesquiterpene chemical structure/5-αH,8-βH-eudesma-1,3,7(11)-trien-8,12-olide.png]

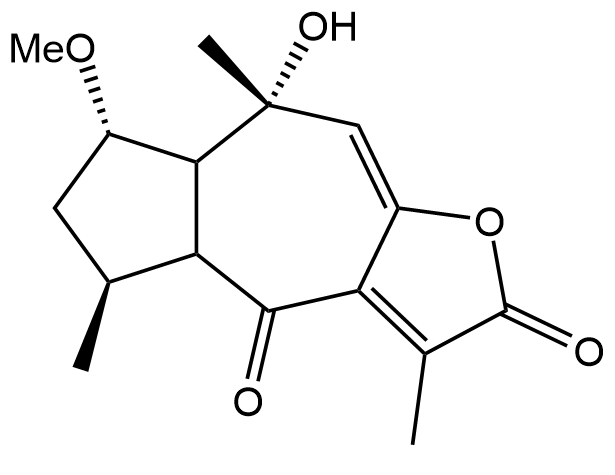

Supplement: Supplementary file 1 [file pharmaceuticals-17-01524-s001.zip › Sesquiterpene chemical structure/5β-10α-hydroxy-2α-methoxy-6-oxoguaia-7(11),8-dien-8,12-olide.png]

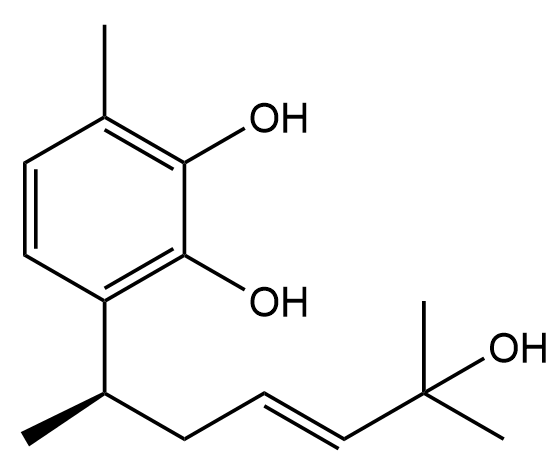

Supplement: Supplementary file 1 [file pharmaceuticals-17-01524-s001.zip › Sesquiterpene chemical structure/6-hydroxy-2-methyl-5-(5′-hydroxy-1′(R),5′-dimethylhex.png]

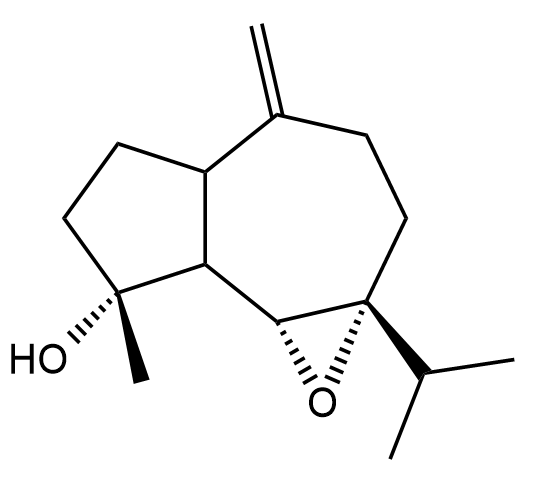

Supplement: Supplementary file 1 [file pharmaceuticals-17-01524-s001.zip › Sesquiterpene chemical structure/6α,7α-epoxy-1β-guai-10(14)-en-4α-ol.png]

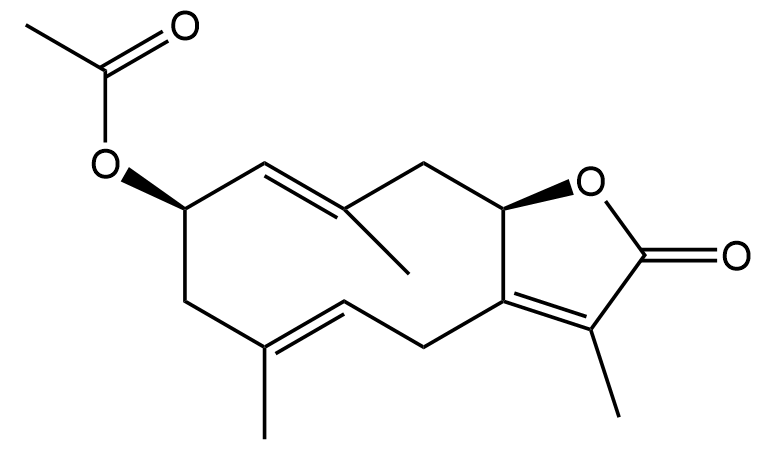

Supplement: Supplementary file 1 [file pharmaceuticals-17-01524-s001.zip › Sesquiterpene chemical structure/8-epi-2-acetyloxyglechomanolide.png]

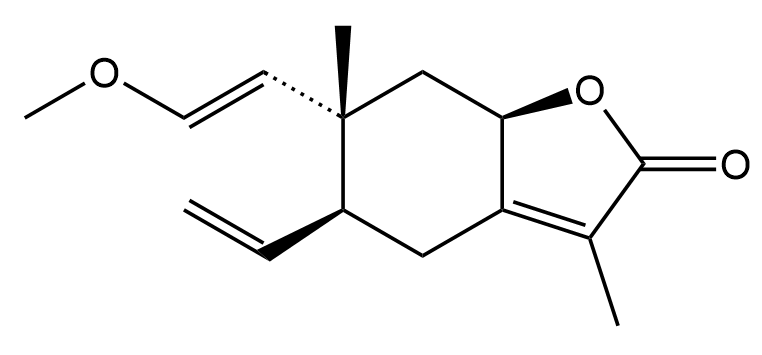

Supplement: Supplementary file 1 [file pharmaceuticals-17-01524-s001.zip › Sesquiterpene chemical structure/8-epi-2-methoxyisogermafurenolide.png]

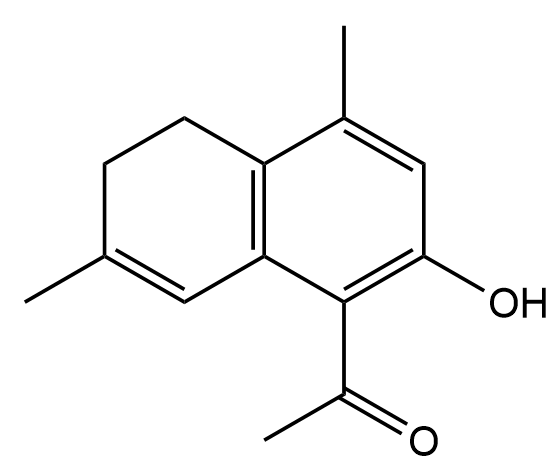

Supplement: Supplementary file 1 [file pharmaceuticals-17-01524-s001.zip › Sesquiterpene chemical structure/8-hydroxy-12-norcardina-4,6,8,10-tetraen-11-one.png]

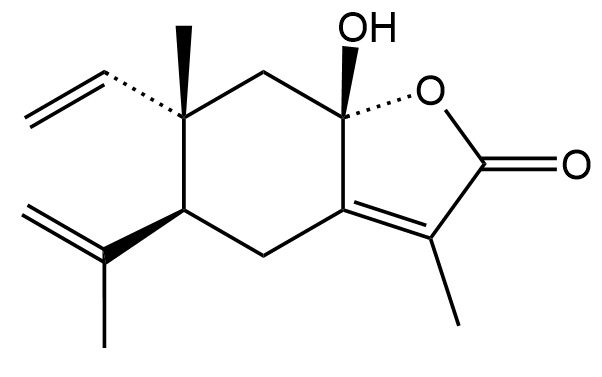

Supplement: Supplementary file 1 [file pharmaceuticals-17-01524-s001.zip › Sesquiterpene chemical structure/8-hydroxyisogermafurenolide.png]

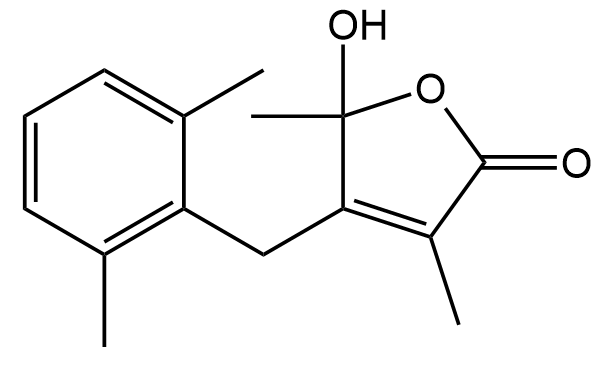

Supplement: Supplementary file 1 [file pharmaceuticals-17-01524-s001.zip › Sesquiterpene chemical structure/9,10-seco-isohydroxylindestrenolide.png]

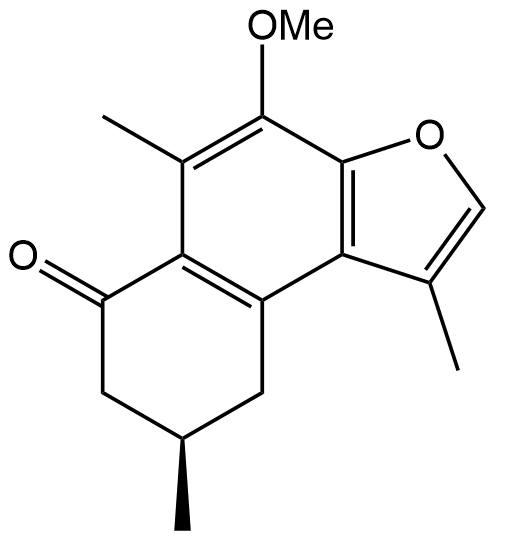

Supplement: Supplementary file 1 [file pharmaceuticals-17-01524-s001.zip › Sesquiterpene chemical structure/9-methoxymyrrhone.png]

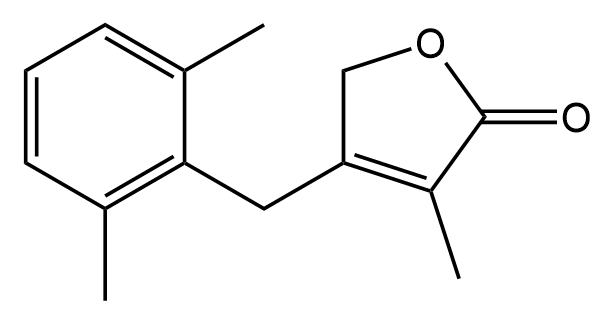

Supplement: Supplementary file 1 [file pharmaceuticals-17-01524-s001.zip › Sesquiterpene chemical structure/9-nor-9,10-seco-isolindestrenolide.png]

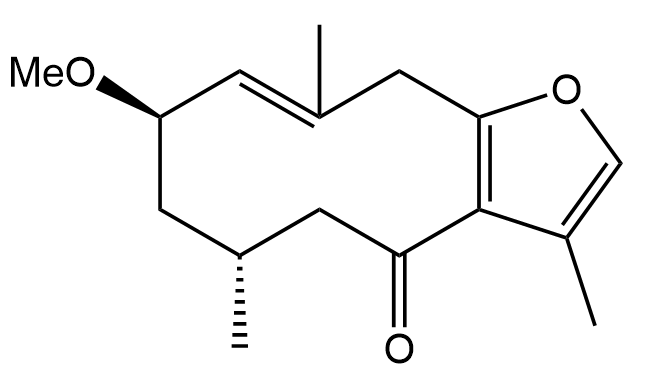

Supplement: Supplementary file 1 [file pharmaceuticals-17-01524-s001.zip › Sesquiterpene chemical structure/A.png]

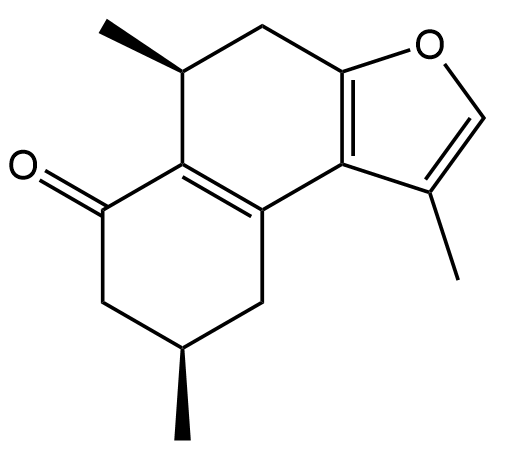

Supplement: Supplementary file 1 [file pharmaceuticals-17-01524-s001.zip › Sesquiterpene chemical structure/agarsenone.png]

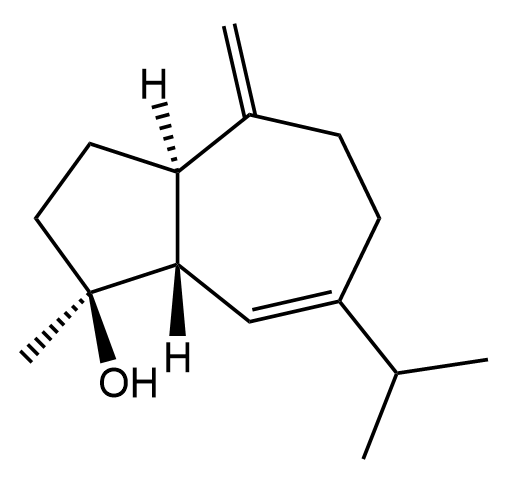

Supplement: Supplementary file 1 [file pharmaceuticals-17-01524-s001.zip › Sesquiterpene chemical structure/alismol.png]

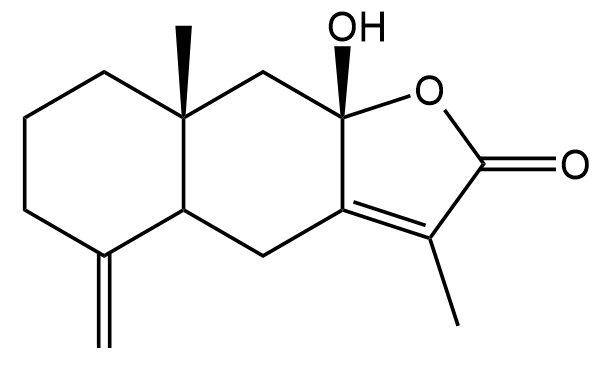

Supplement: Supplementary file 1 [file pharmaceuticals-17-01524-s001.zip › Sesquiterpene chemical structure/atractylenolide.png]

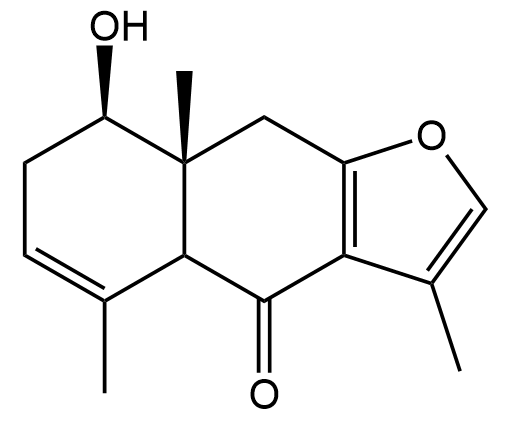

Supplement: Supplementary file 1 [file pharmaceuticals-17-01524-s001.zip › Sesquiterpene chemical structure/chlomultinB.png]

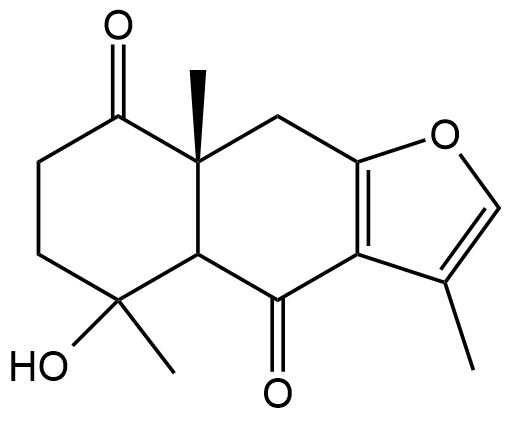

Supplement: Supplementary file 1 [file pharmaceuticals-17-01524-s001.zip › Sesquiterpene chemical structure/chloranteneC.png]

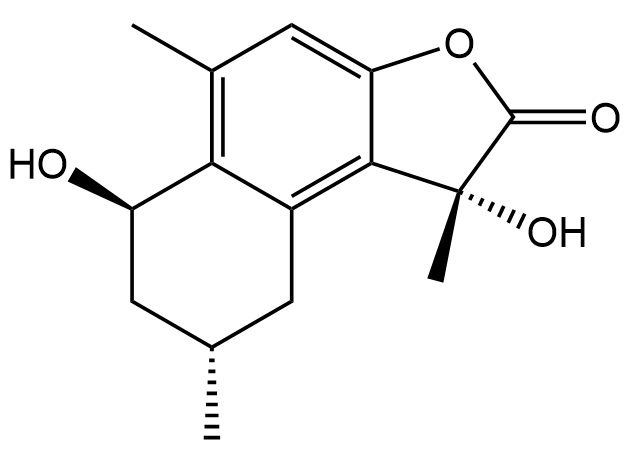

Supplement: Supplementary file 1 [file pharmaceuticals-17-01524-s001.zip › Sesquiterpene chemical structure/commiphoinA.png]

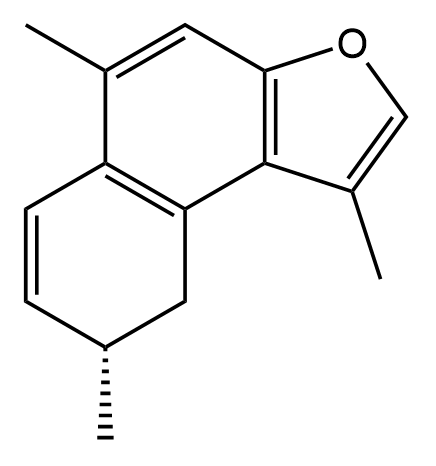

Supplement: Supplementary file 1 [file pharmaceuticals-17-01524-s001.zip › Sesquiterpene chemical structure/commiphoinB.png]

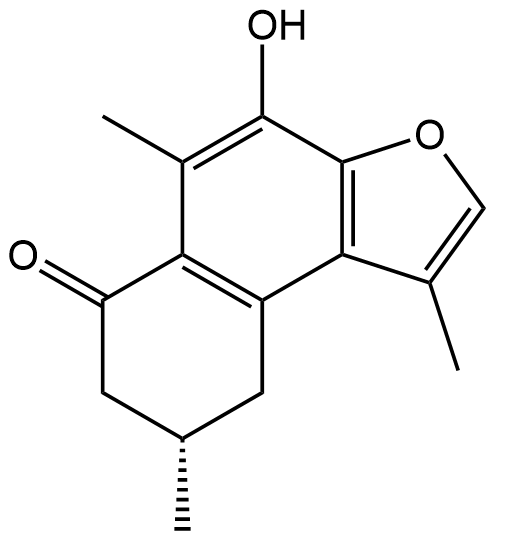

Supplement: Supplementary file 1 [file pharmaceuticals-17-01524-s001.zip › Sesquiterpene chemical structure/commiphoinC.png]

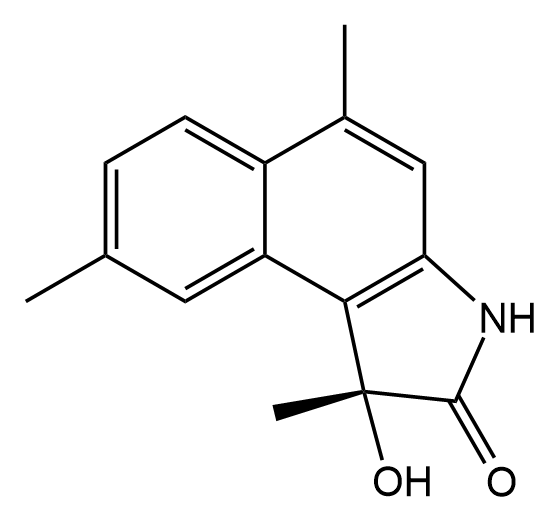

Supplement: Supplementary file 1 [file pharmaceuticals-17-01524-s001.zip › Sesquiterpene chemical structure/commipholactamA.png]

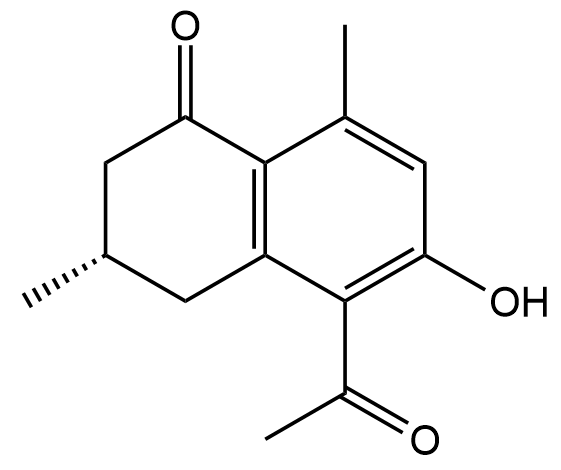

Supplement: Supplementary file 1 [file pharmaceuticals-17-01524-s001.zip › Sesquiterpene chemical structure/commipholinone.png]

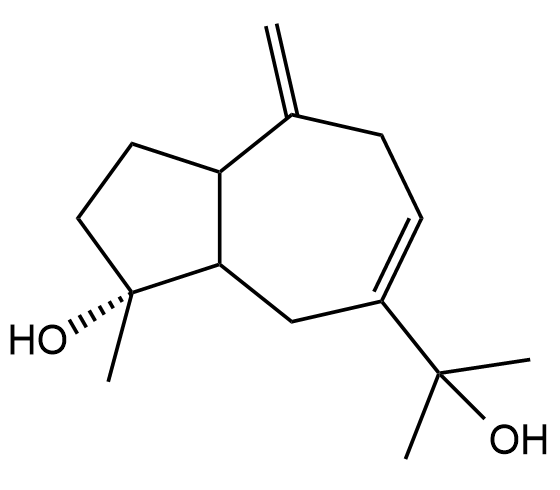

Supplement: Supplementary file 1 [file pharmaceuticals-17-01524-s001.zip › Sesquiterpene chemical structure/commiphorane J.png]

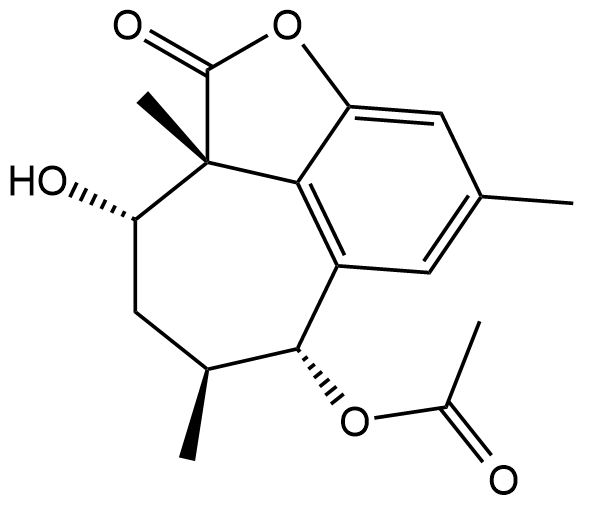

Supplement: Supplementary file 1 [file pharmaceuticals-17-01524-s001.zip › Sesquiterpene chemical structure/commiphoraneC.png]

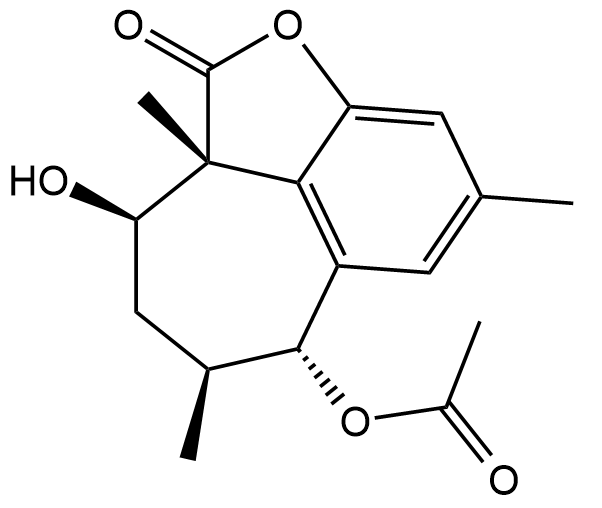

Supplement: Supplementary file 1 [file pharmaceuticals-17-01524-s001.zip › Sesquiterpene chemical structure/commiphoraneD.png]

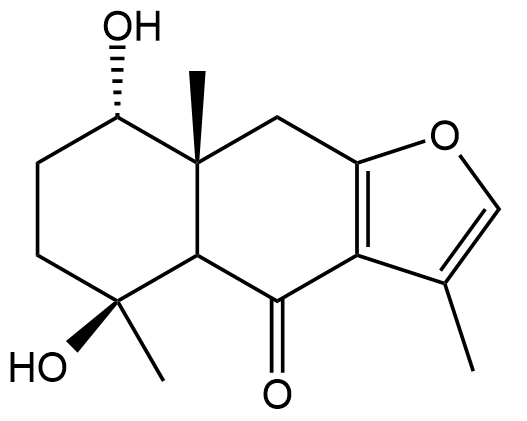

Supplement: Supplementary file 1 [file pharmaceuticals-17-01524-s001.zip › Sesquiterpene chemical structure/commiphoraneE1.png]

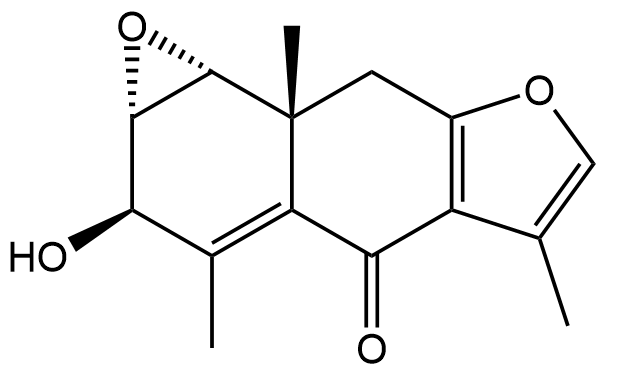

Supplement: Supplementary file 1 [file pharmaceuticals-17-01524-s001.zip › Sesquiterpene chemical structure/commiphoraneE2.png]

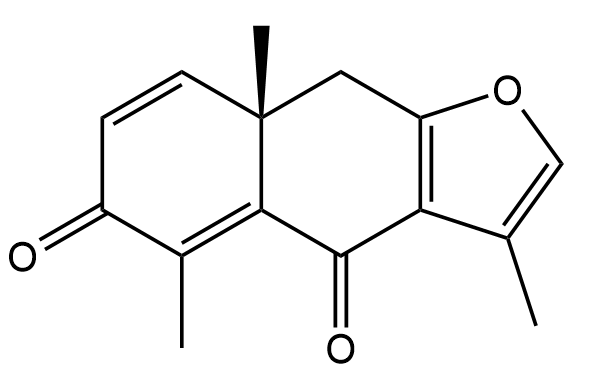

Supplement: Supplementary file 1 [file pharmaceuticals-17-01524-s001.zip › Sesquiterpene chemical structure/commiphoraneE3.png]

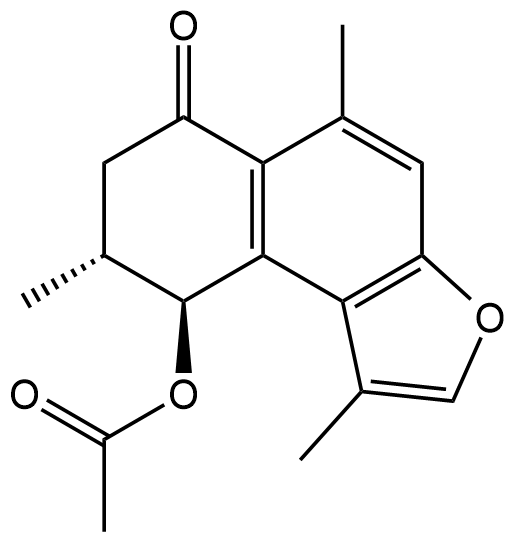

Supplement: Supplementary file 1 [file pharmaceuticals-17-01524-s001.zip › Sesquiterpene chemical structure/commiphoraneH.png]

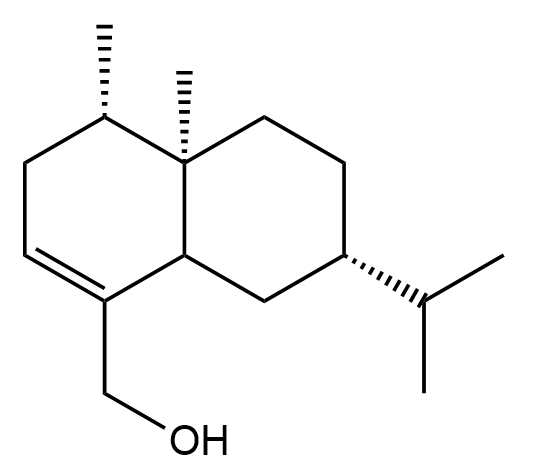

Supplement: Supplementary file 1 [file pharmaceuticals-17-01524-s001.zip › Sesquiterpene chemical structure/commiphoraneI.png]

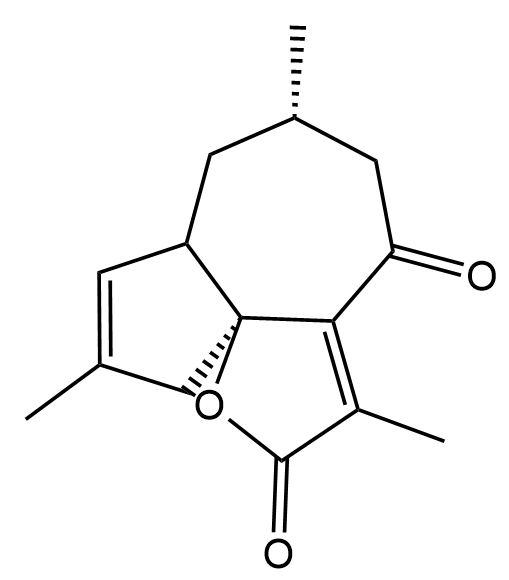

Supplement: Supplementary file 1 [file pharmaceuticals-17-01524-s001.zip › Sesquiterpene chemical structure/commiphoranoidA.png]

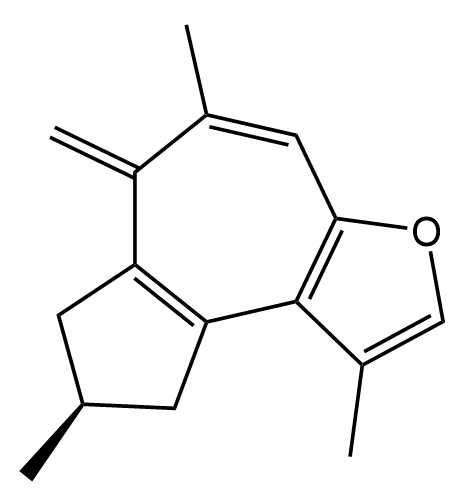

Supplement: Supplementary file 1 [file pharmaceuticals-17-01524-s001.zip › Sesquiterpene chemical structure/commiphoranoidB.png]

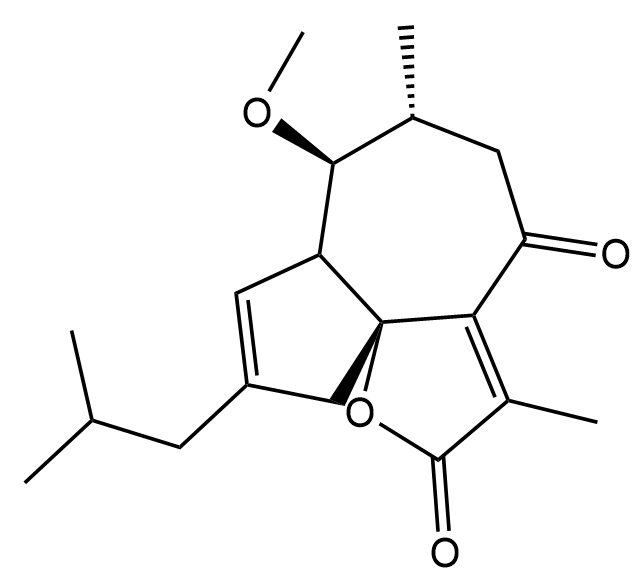

Supplement: Supplementary file 1 [file pharmaceuticals-17-01524-s001.zip › Sesquiterpene chemical structure/commiphoranoidC.png]

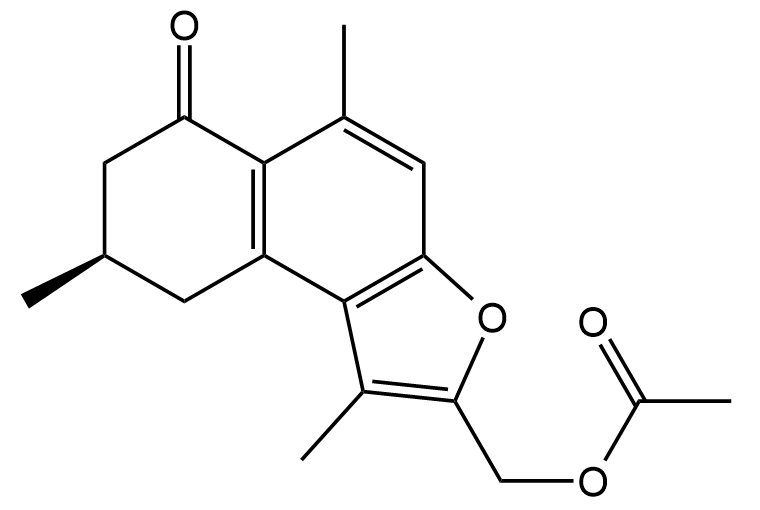

Supplement: Supplementary file 1 [file pharmaceuticals-17-01524-s001.zip › Sesquiterpene chemical structure/commiphoreneA.png]

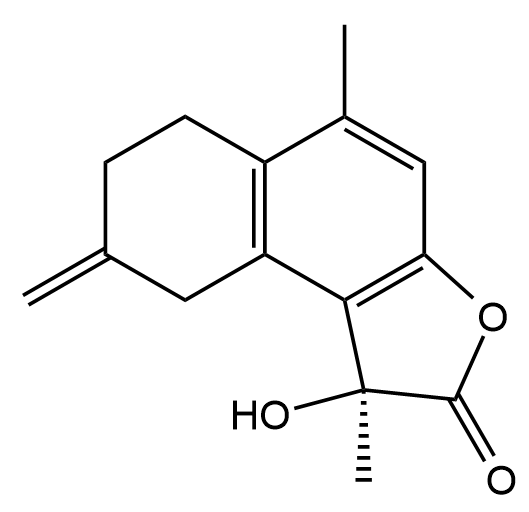

Supplement: Supplementary file 1 [file pharmaceuticals-17-01524-s001.zip › Sesquiterpene chemical structure/commiphoreneB.png]

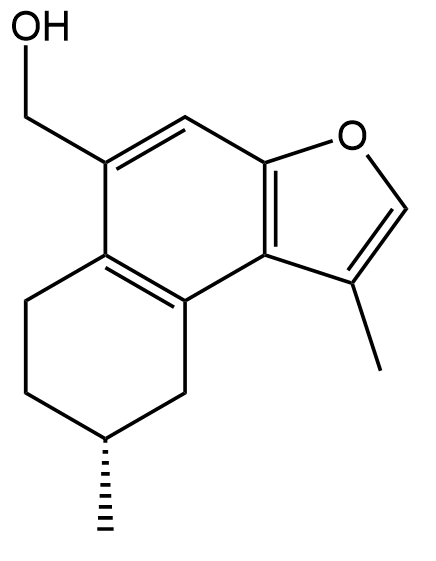

Supplement: Supplementary file 1 [file pharmaceuticals-17-01524-s001.zip › Sesquiterpene chemical structure/commiterpeneA.png]

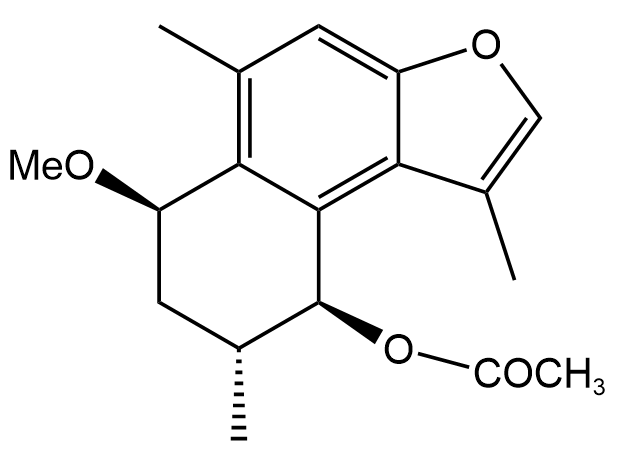

Supplement: Supplementary file 1 [file pharmaceuticals-17-01524-s001.zip › Sesquiterpene chemical structure/commiterpeneB.png]

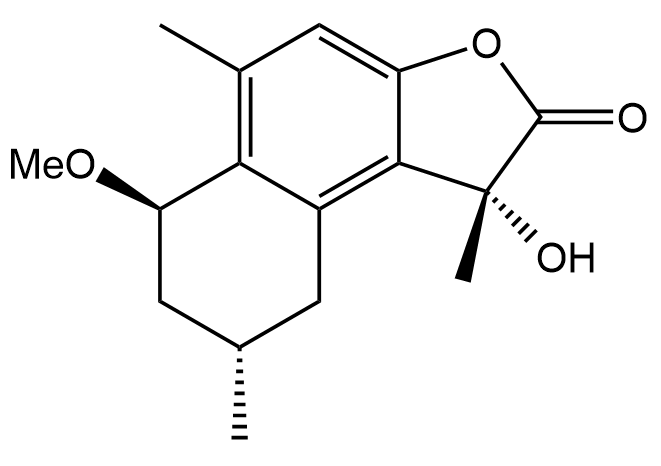

Supplement: Supplementary file 1 [file pharmaceuticals-17-01524-s001.zip › Sesquiterpene chemical structure/commiterpeneC.png]

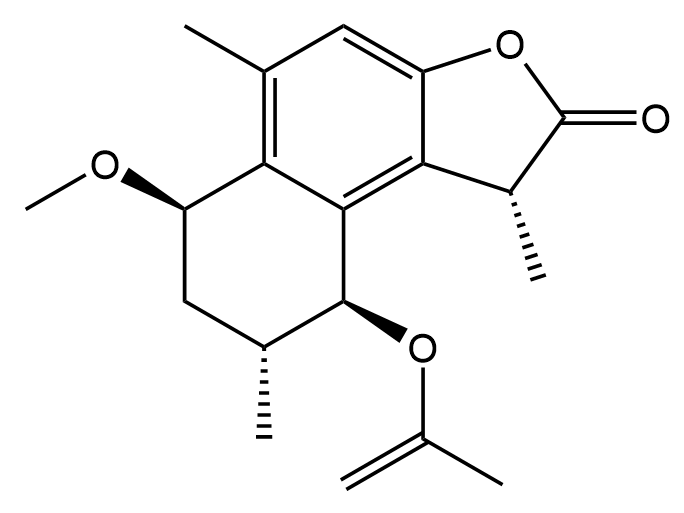

Supplement: Supplementary file 1 [file pharmaceuticals-17-01524-s001.zip › Sesquiterpene chemical structure/commiterpeneD.png]

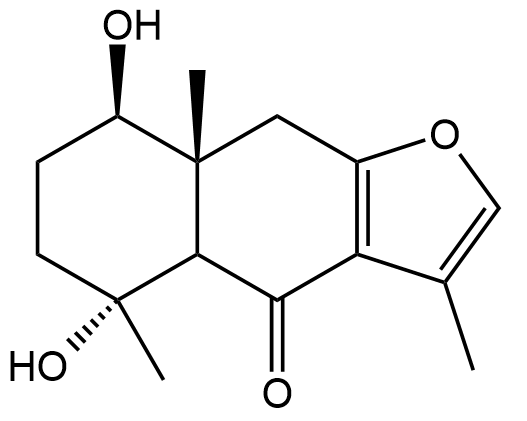

Supplement: Supplementary file 1 [file pharmaceuticals-17-01524-s001.zip › Sesquiterpene chemical structure/curcolonol.png]

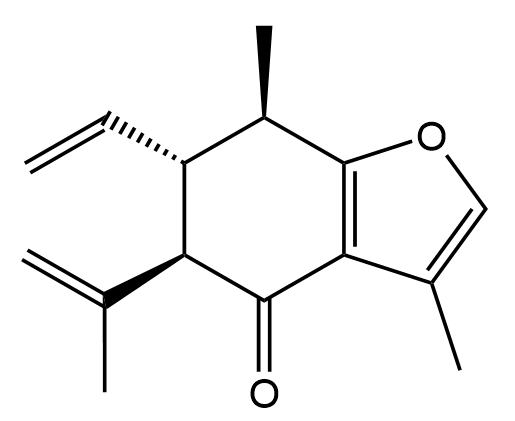

Supplement: Supplementary file 1 [file pharmaceuticals-17-01524-s001.zip › Sesquiterpene chemical structure/curzerenone.png]

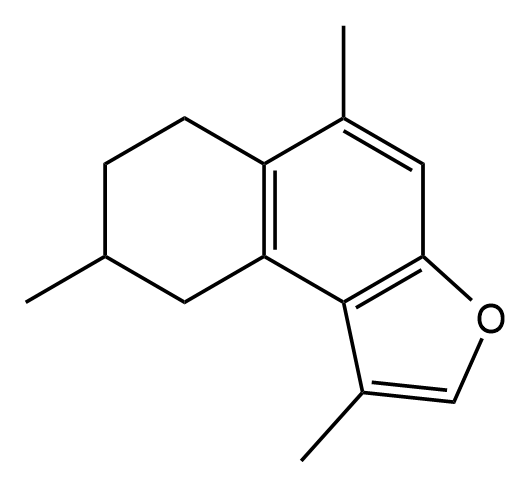

Supplement: Supplementary file 1 [file pharmaceuticals-17-01524-s001.zip › Sesquiterpene chemical structure/dihydropyrocurzerenone.png]

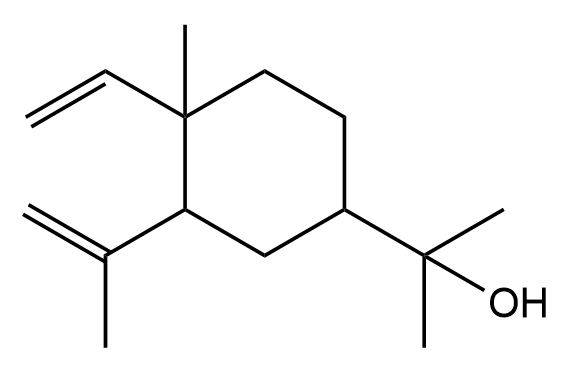

Supplement: Supplementary file 1 [file pharmaceuticals-17-01524-s001.zip › Sesquiterpene chemical structure/elemol.png]

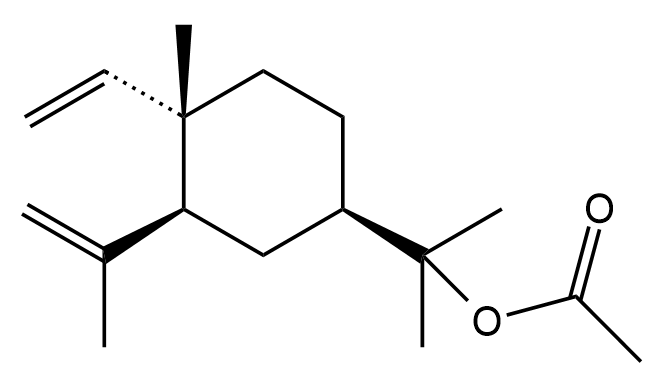

Supplement: Supplementary file 1 [file pharmaceuticals-17-01524-s001.zip › Sesquiterpene chemical structure/elemylacetate.png]

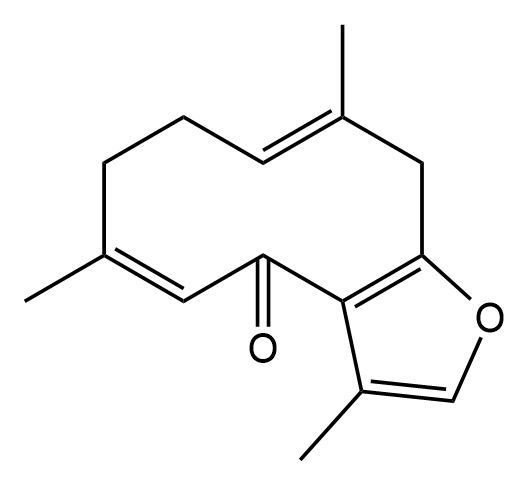

Supplement: Supplementary file 1 [file pharmaceuticals-17-01524-s001.zip › Sesquiterpene chemical structure/epicurzerenone.png]

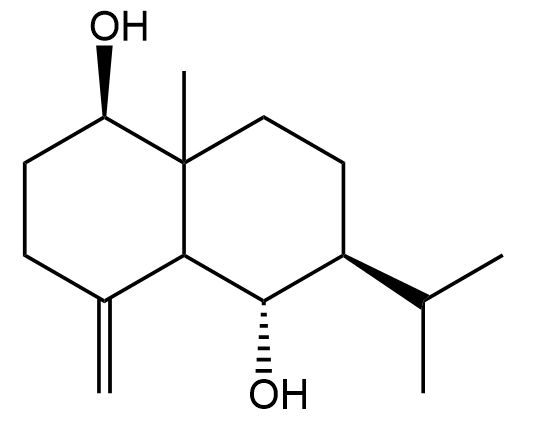

Supplement: Supplementary file 1 [file pharmaceuticals-17-01524-s001.zip › Sesquiterpene chemical structure/eudesm-4(15)-ene-1β,6α-diol.png]

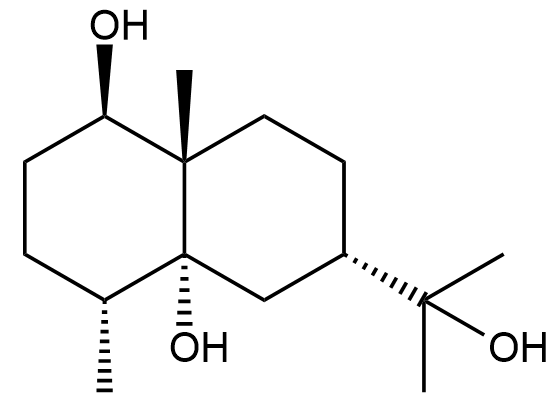

Supplement: Supplementary file 1 [file pharmaceuticals-17-01524-s001.zip › Sesquiterpene chemical structure/eudesmane-1β,5α,11-triol.png]

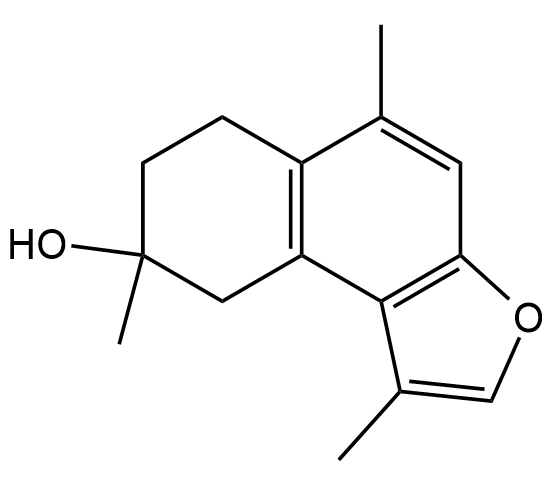

Supplement: Supplementary file 1 [file pharmaceuticals-17-01524-s001.zip › Sesquiterpene chemical structure/furanocadina-1(10),6,8-triene-4-ol.png]

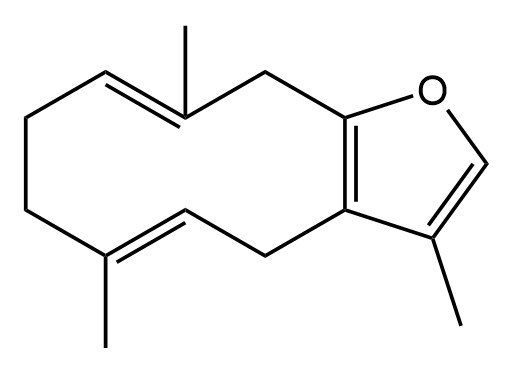

Supplement: Supplementary file 1 [file pharmaceuticals-17-01524-s001.zip › Sesquiterpene chemical structure/furanodiene.png]

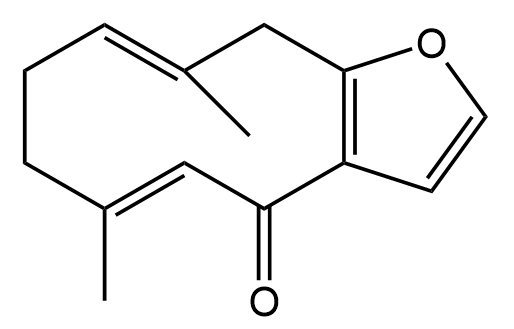

Supplement: Supplementary file 1 [file pharmaceuticals-17-01524-s001.zip › Sesquiterpene chemical structure/furanodieneone.png]

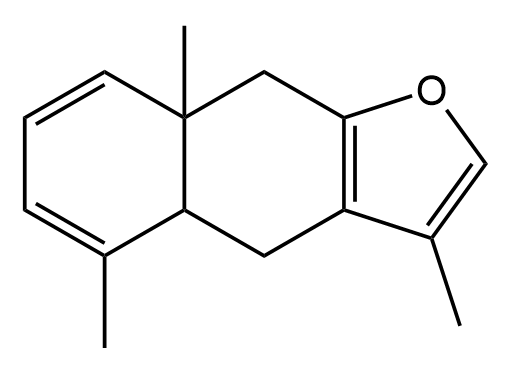

Supplement: Supplementary file 1 [file pharmaceuticals-17-01524-s001.zip › Sesquiterpene chemical structure/furanoeudesma-1,3-diene.png]

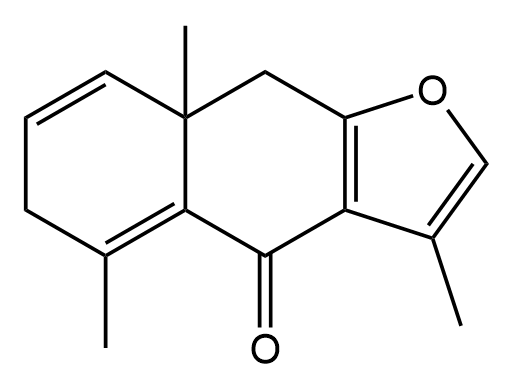

Supplement: Supplementary file 1 [file pharmaceuticals-17-01524-s001.zip › Sesquiterpene chemical structure/furanoeudesma-1,4-diene-6-one.png]

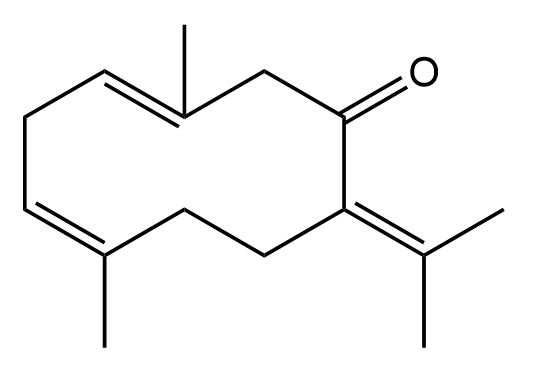

Supplement: Supplementary file 1 [file pharmaceuticals-17-01524-s001.zip › Sesquiterpene chemical structure/germacrone.png]

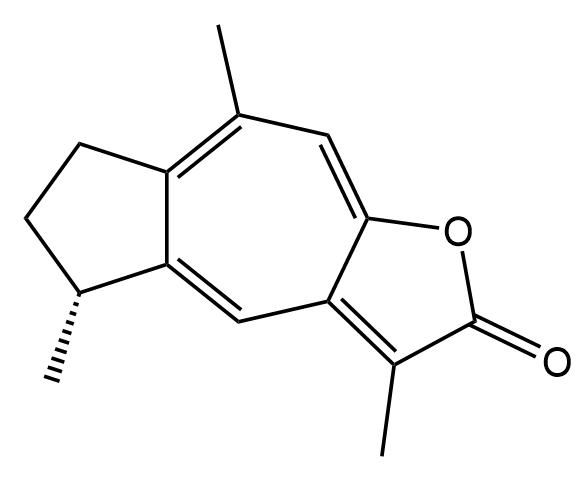

Supplement: Supplementary file 1 [file pharmaceuticals-17-01524-s001.zip › Sesquiterpene chemical structure/guai-1(10),5,7(11),8-tetradien-12,8-olide.png]

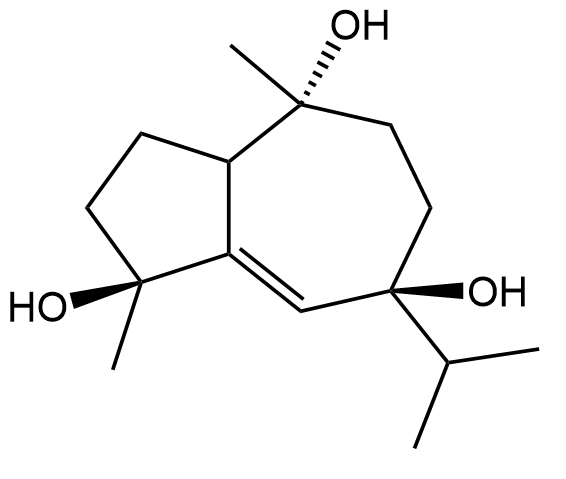

Supplement: Supplementary file 1 [file pharmaceuticals-17-01524-s001.zip › Sesquiterpene chemical structure/guaia-4β,7β,10α-trihydroxy-5-ene.png]

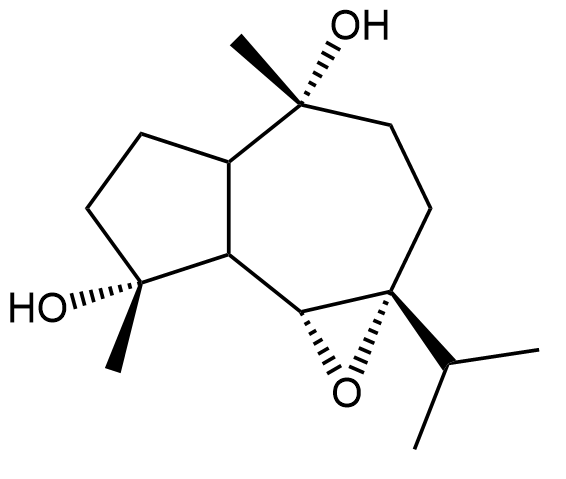

Supplement: Supplementary file 1 [file pharmaceuticals-17-01524-s001.zip › Sesquiterpene chemical structure/guaia-6α,7α-epoxy-4α,10α-diol.png]

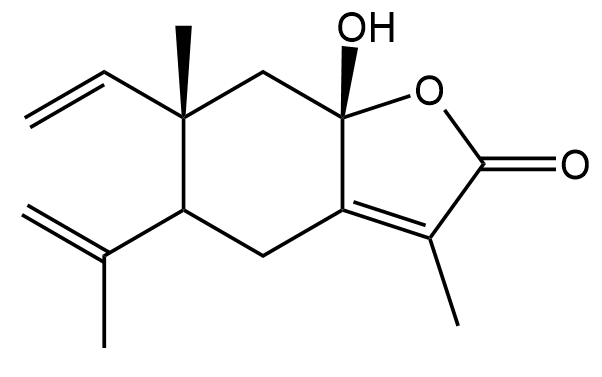

Supplement: Supplementary file 1 [file pharmaceuticals-17-01524-s001.zip › Sesquiterpene chemical structure/hydroxyisogermafurenolide.png]

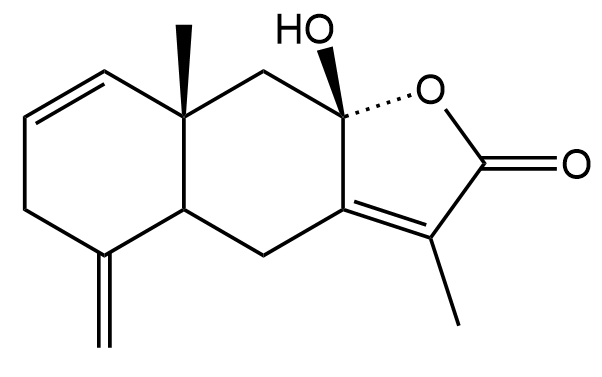

Supplement: Supplementary file 1 [file pharmaceuticals-17-01524-s001.zip › Sesquiterpene chemical structure/hydroxylindestrenolide.png]

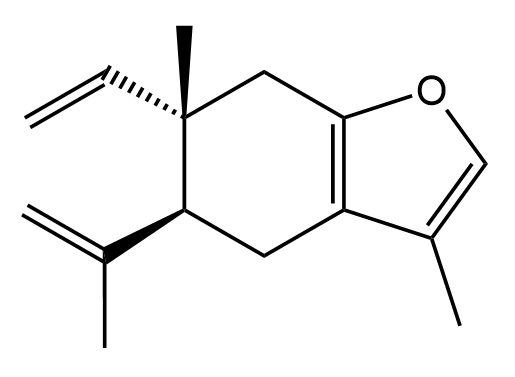

Supplement: Supplementary file 1 [file pharmaceuticals-17-01524-s001.zip › Sesquiterpene chemical structure/isofuranogermacrene.png]

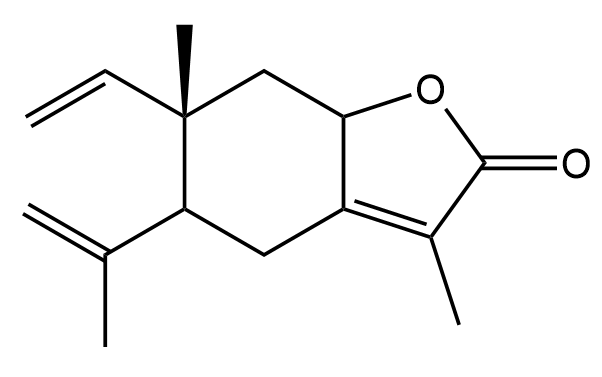

Supplement: Supplementary file 1 [file pharmaceuticals-17-01524-s001.zip › Sesquiterpene chemical structure/isogermafurenolide.png]

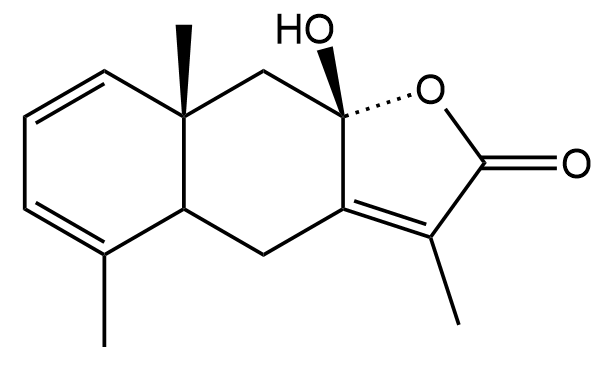

Supplement: Supplementary file 1 [file pharmaceuticals-17-01524-s001.zip › Sesquiterpene chemical structure/isohydroxylindestrenolide.png]

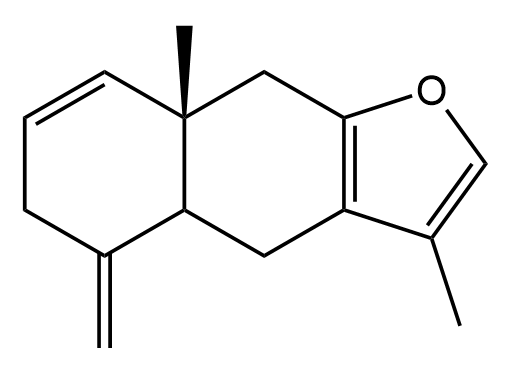

Supplement: Supplementary file 1 [file pharmaceuticals-17-01524-s001.zip › Sesquiterpene chemical structure/lindestrene.png]

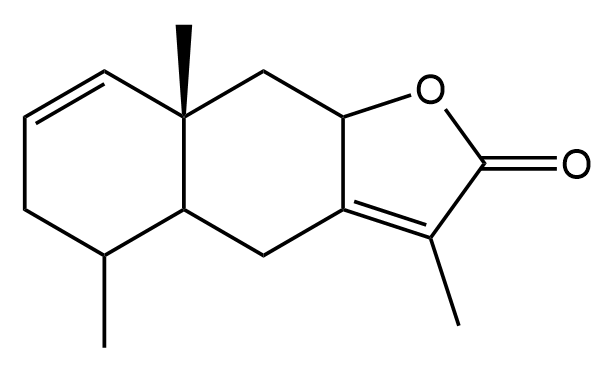

Supplement: Supplementary file 1 [file pharmaceuticals-17-01524-s001.zip › Sesquiterpene chemical structure/lindestrenolide.png]

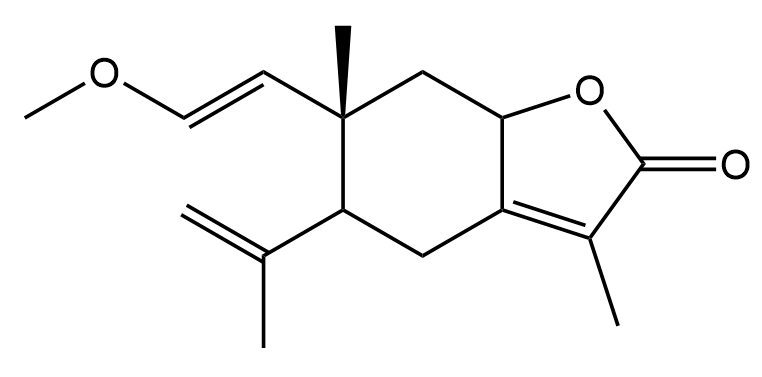

Supplement: Supplementary file 1 [file pharmaceuticals-17-01524-s001.zip › Sesquiterpene chemical structure/methoxyisogermafurenolide.png]

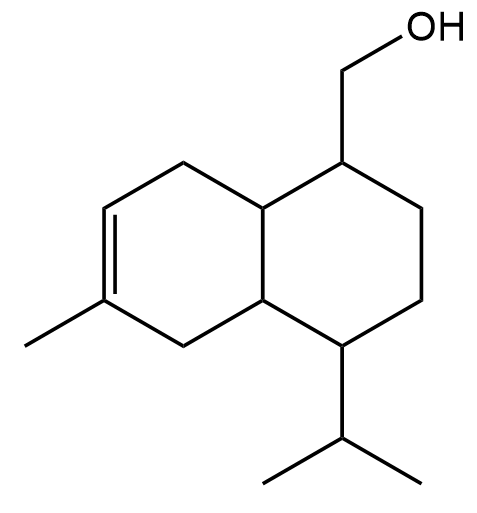

Supplement: Supplementary file 1 [file pharmaceuticals-17-01524-s001.zip › Sesquiterpene chemical structure/myrracadinolA.png]

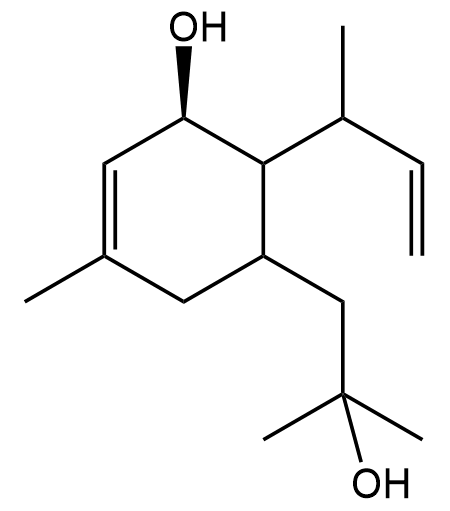

Supplement: Supplementary file 1 [file pharmaceuticals-17-01524-s001.zip › Sesquiterpene chemical structure/myrracadinolB.png]

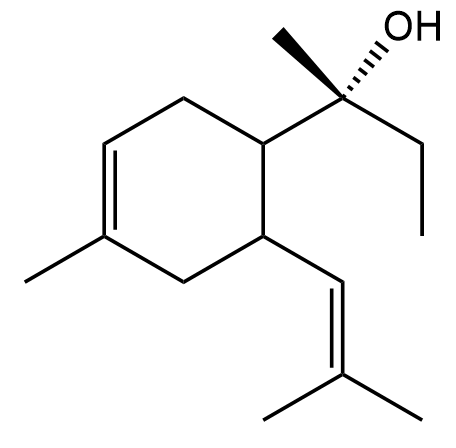

Supplement: Supplementary file 1 [file pharmaceuticals-17-01524-s001.zip › Sesquiterpene chemical structure/myrracadinolC.png]

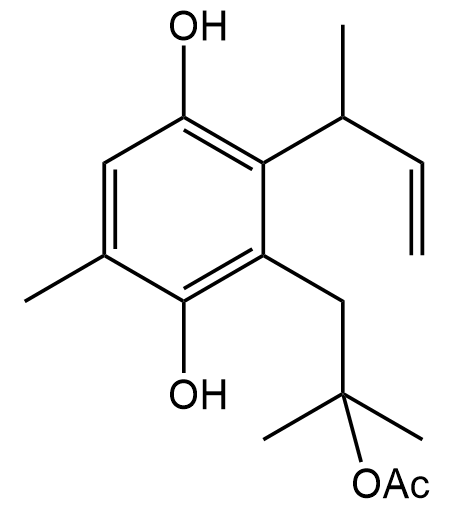

Supplement: Supplementary file 1 [file pharmaceuticals-17-01524-s001.zip › Sesquiterpene chemical structure/myrracalameneA.png]

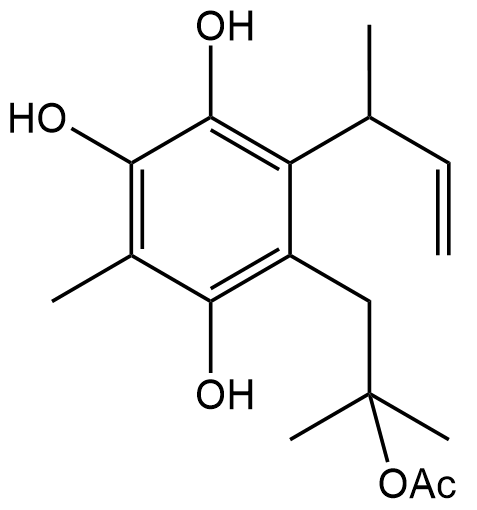

Supplement: Supplementary file 1 [file pharmaceuticals-17-01524-s001.zip › Sesquiterpene chemical structure/myrracalameneB.png]

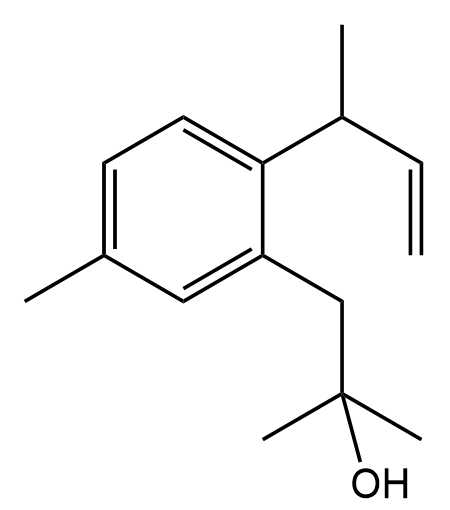

Supplement: Supplementary file 1 [file pharmaceuticals-17-01524-s001.zip › Sesquiterpene chemical structure/myrracalameneC.png]

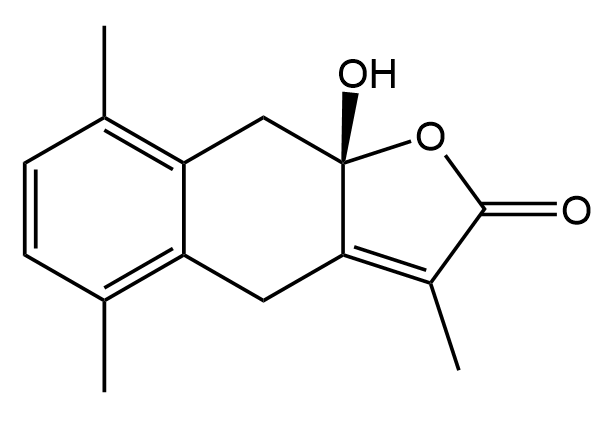

Supplement: Supplementary file 1 [file pharmaceuticals-17-01524-s001.zip › Sesquiterpene chemical structure/myrrhanolideA.png]

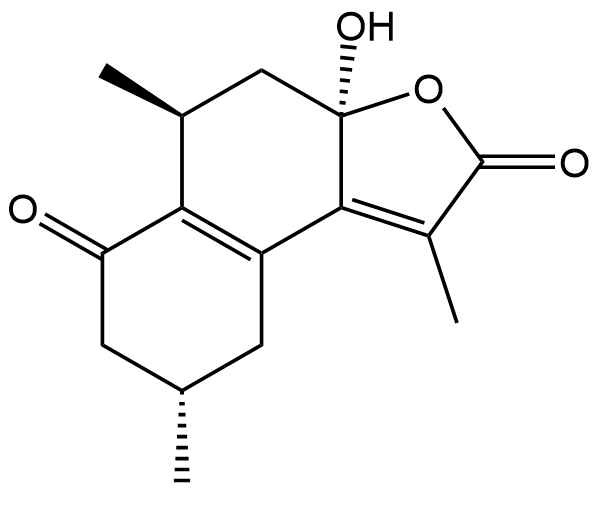

Supplement: Supplementary file 1 [file pharmaceuticals-17-01524-s001.zip › Sesquiterpene chemical structure/myrrhanolideB.png]

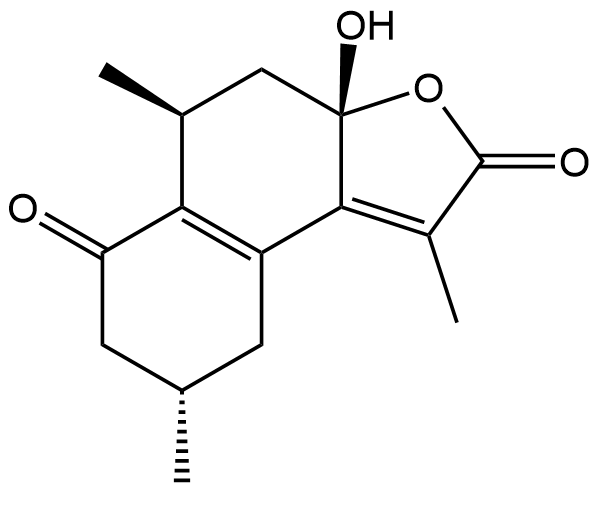

Supplement: Supplementary file 1 [file pharmaceuticals-17-01524-s001.zip › Sesquiterpene chemical structure/myrrhanolideC.png]

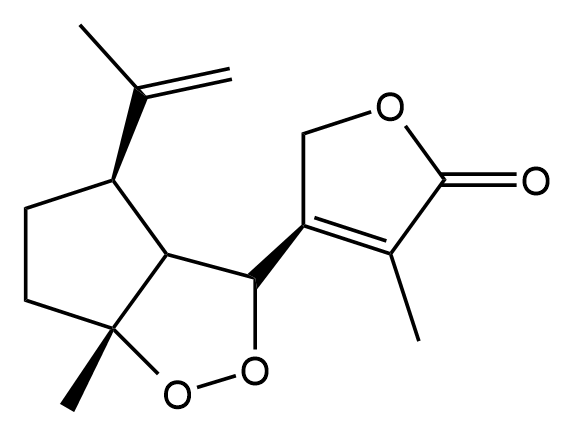

Supplement: Supplementary file 1 [file pharmaceuticals-17-01524-s001.zip › Sesquiterpene chemical structure/myrrhanoperoxide.png]

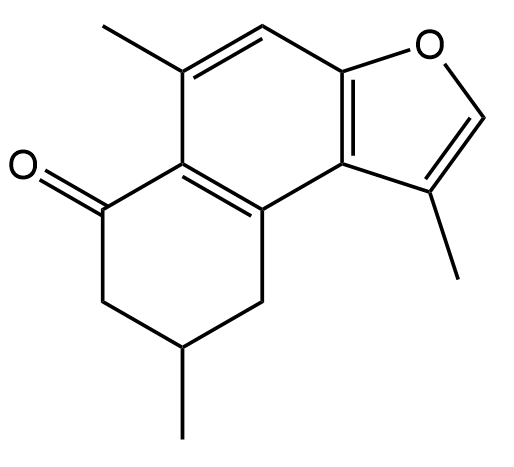

Supplement: Supplementary file 1 [file pharmaceuticals-17-01524-s001.zip › Sesquiterpene chemical structure/myrrhone.png]

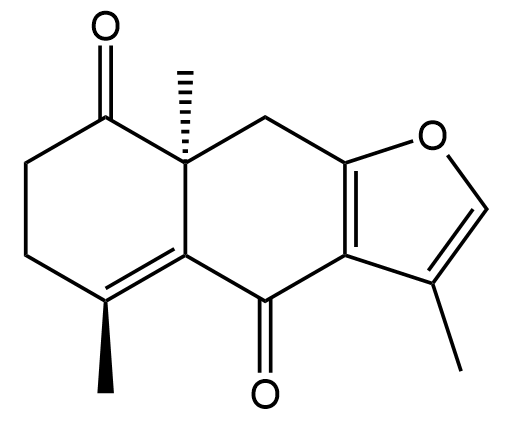

Supplement: Supplementary file 1 [file pharmaceuticals-17-01524-s001.zip › Sesquiterpene chemical structure/myrrhterpenoidA.png]

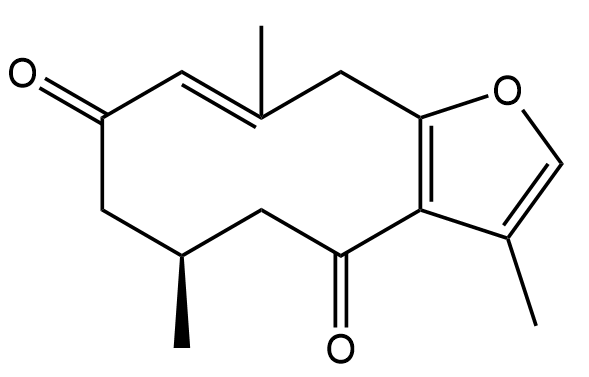

Supplement: Supplementary file 1 [file pharmaceuticals-17-01524-s001.zip › Sesquiterpene chemical structure/myrrhterpenoidB.png]

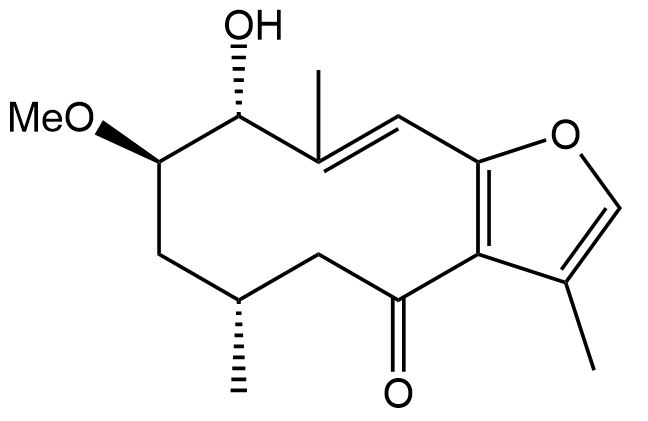

Supplement: Supplementary file 1 [file pharmaceuticals-17-01524-s001.zip › Sesquiterpene chemical structure/myrrhterpenoidC.png]

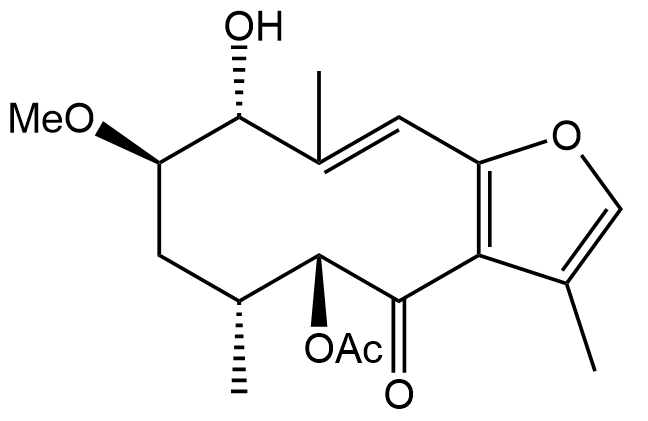

Supplement: Supplementary file 1 [file pharmaceuticals-17-01524-s001.zip › Sesquiterpene chemical structure/myrrhterpenoidD.png]

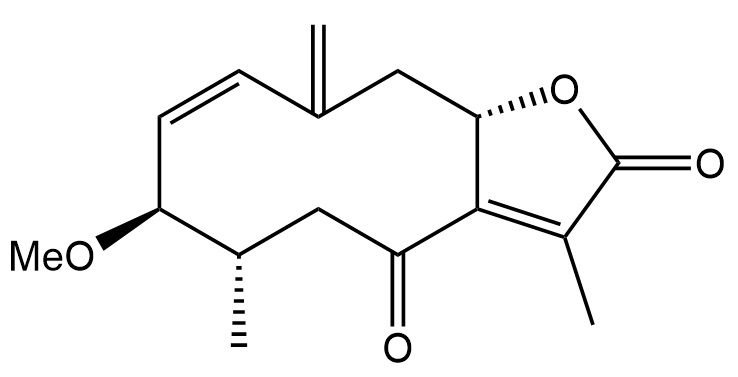

Supplement: Supplementary file 1 [file pharmaceuticals-17-01524-s001.zip › Sesquiterpene chemical structure/myrrhterpenoidE.png]

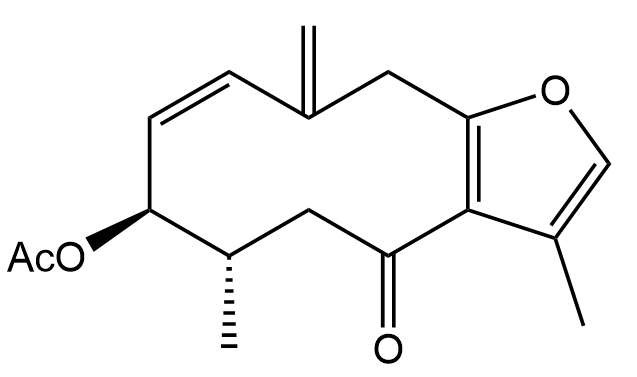

Supplement: Supplementary file 1 [file pharmaceuticals-17-01524-s001.zip › Sesquiterpene chemical structure/myrrhterpenoidF.png]

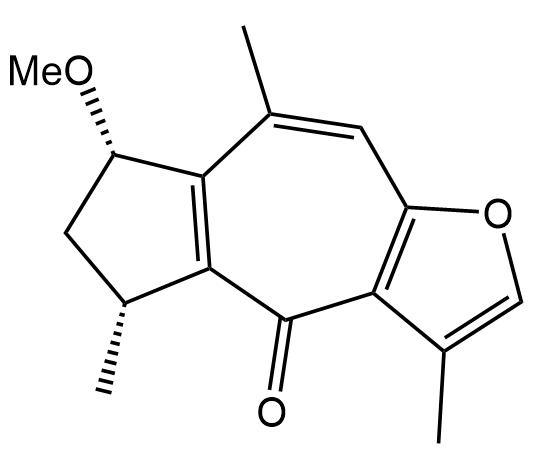

Supplement: Supplementary file 1 [file pharmaceuticals-17-01524-s001.zip › Sesquiterpene chemical structure/myrrhterpenoidG.png]

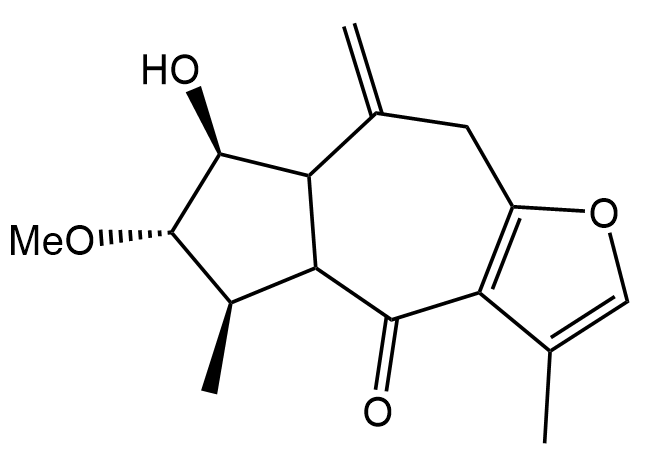

Supplement: Supplementary file 1 [file pharmaceuticals-17-01524-s001.zip › Sesquiterpene chemical structure/myrrhterpenoidH.png]

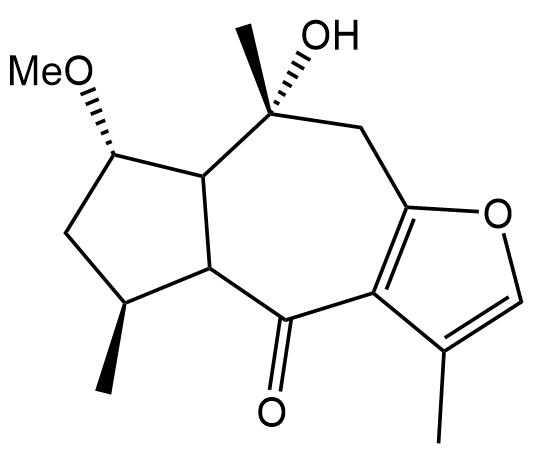

Supplement: Supplementary file 1 [file pharmaceuticals-17-01524-s001.zip › Sesquiterpene chemical structure/myrrhterpenoidI.png]

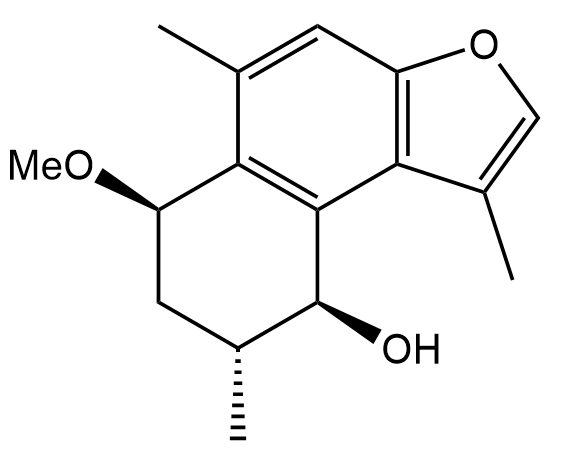

Supplement: Supplementary file 1 [file pharmaceuticals-17-01524-s001.zip › Sesquiterpene chemical structure/myrrhterpenoidJ.png]

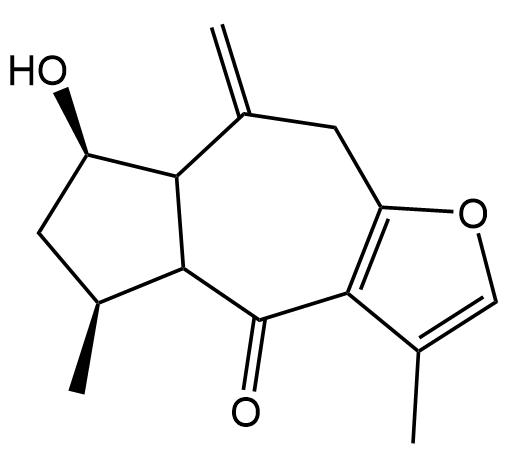

Supplement: Supplementary file 1 [file pharmaceuticals-17-01524-s001.zip › Sesquiterpene chemical structure/myrrhterpenoidK.png]

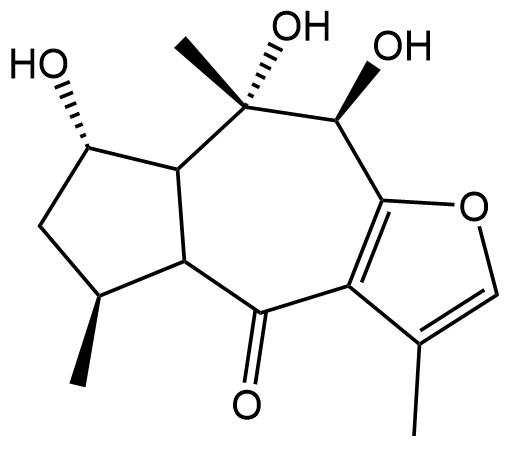

Supplement: Supplementary file 1 [file pharmaceuticals-17-01524-s001.zip › Sesquiterpene chemical structure/myrrhterpenoidL.png]

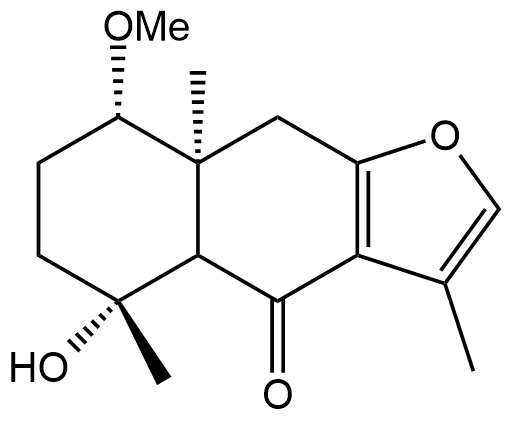

Supplement: Supplementary file 1 [file pharmaceuticals-17-01524-s001.zip › Sesquiterpene chemical structure/myrrhterpenoidM.png]

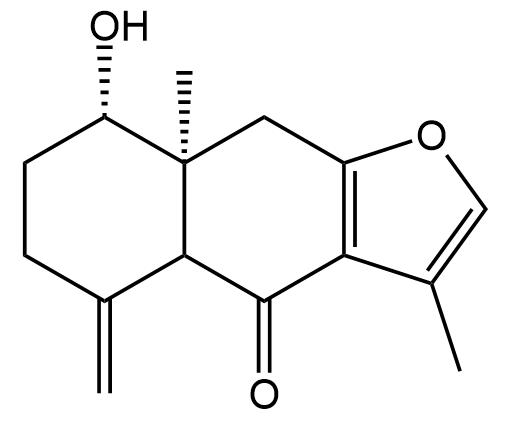

Supplement: Supplementary file 1 [file pharmaceuticals-17-01524-s001.zip › Sesquiterpene chemical structure/myrrhterpenoidN.png]

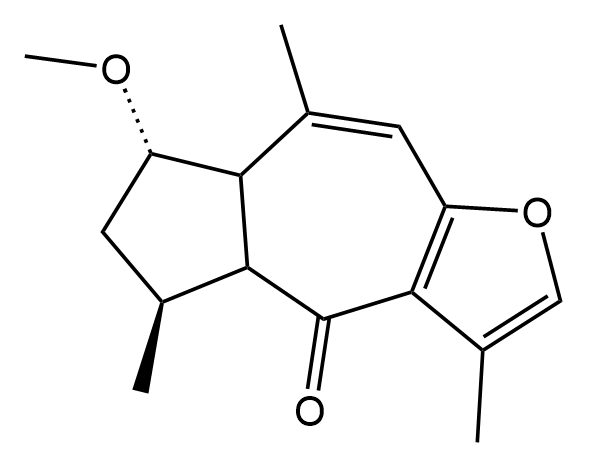

Supplement: Supplementary file 1 [file pharmaceuticals-17-01524-s001.zip › Sesquiterpene chemical structure/myrrhterpenoidO.png]

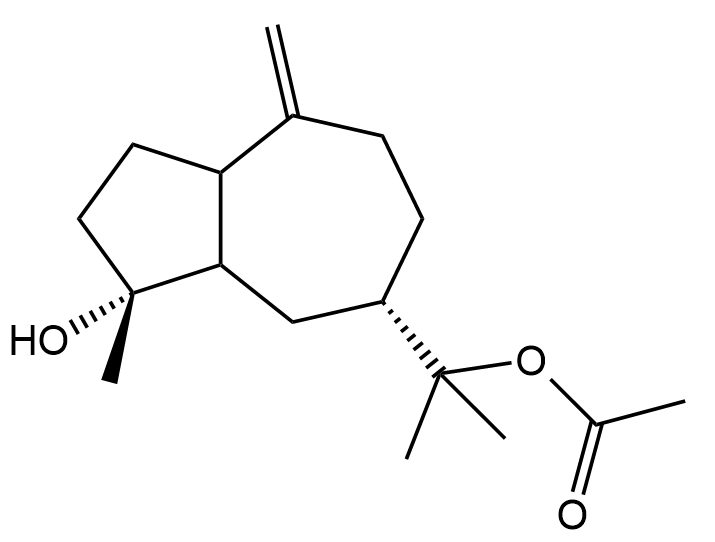

Supplement: Supplementary file 1 [file pharmaceuticals-17-01524-s001.zip › Sesquiterpene chemical structure/rel-(+)-(1S,4R,7S)-11-acetyl-guai-10(14)-en-4,11-ol.png]

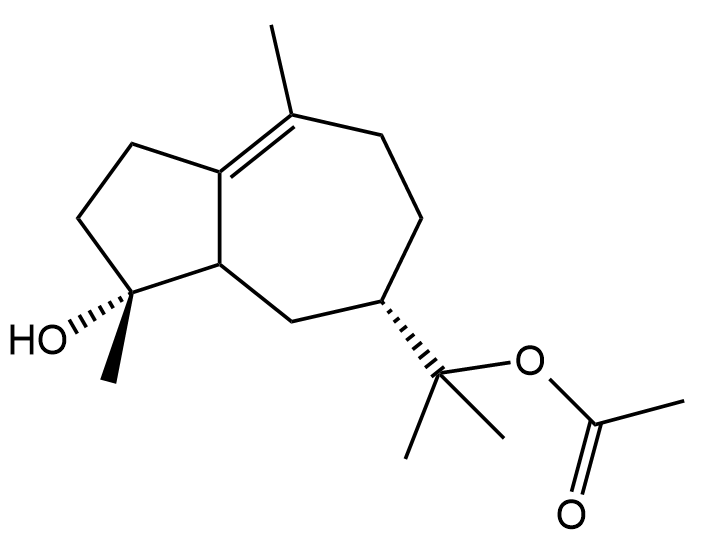

Supplement: Supplementary file 1 [file pharmaceuticals-17-01524-s001.zip › Sesquiterpene chemical structure/rel-(+)-(4R,5R,7S)-11-acetyl-guai-1(10)-en-4,11-ol.png]

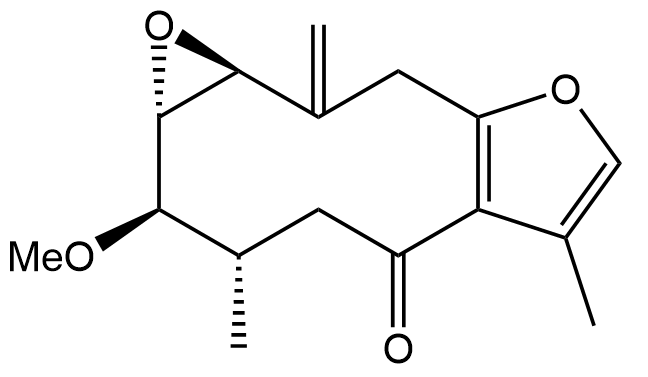

Supplement: Supplementary file 1 [file pharmaceuticals-17-01524-s001.zip › Sesquiterpene chemical structure/rel-(1S,2S,3R,4S)-1,2-epoxy-3-methoxyfuranogermacr-10.png]

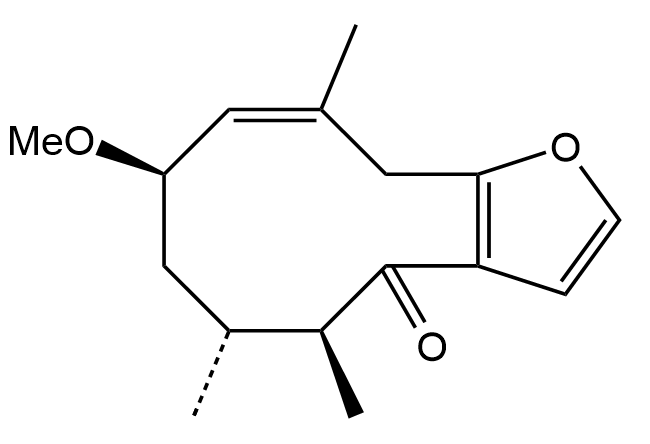

Supplement: Supplementary file 1 [file pharmaceuticals-17-01524-s001.zip › Sesquiterpene chemical structure/rel-2R-methyl-5S-acetoxy-4R-furanogermacr-1(10)Z-en-6 one.png]

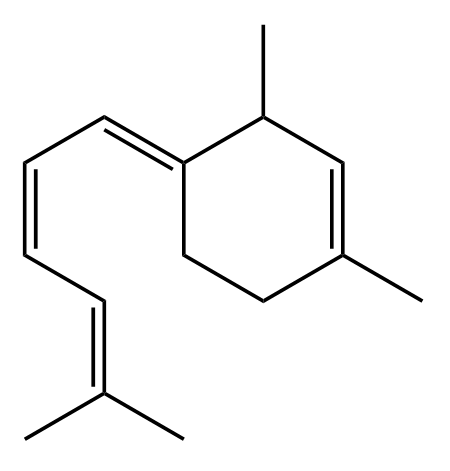

Supplement: Supplementary file 1 [file pharmaceuticals-17-01524-s001.zip › Sesquiterpene chemical structure/α-bisabolene.png]

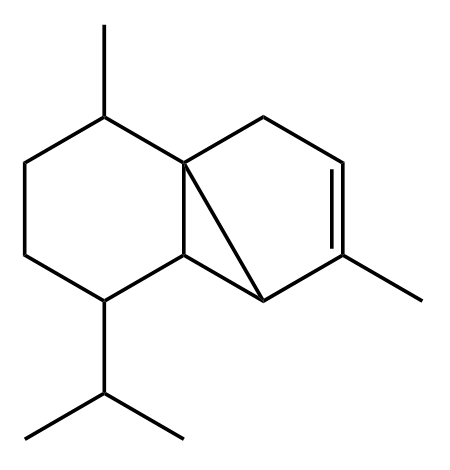

Supplement: Supplementary file 1 [file pharmaceuticals-17-01524-s001.zip › Sesquiterpene chemical structure/α-cubebene.png]
