# Supplementary figures and images for: The Genus Commiphora: An Overview of Its Traditional Uses, Phytochemistry, Pharmacology, and Quality Control (part 1 of 2)
Source: Pharmaceuticals (Basel). 2024 Nov 12;17(11):1524. doi: 10.3390/ph17111524 (PMC11597752; doi:10.3390/ph17111524)

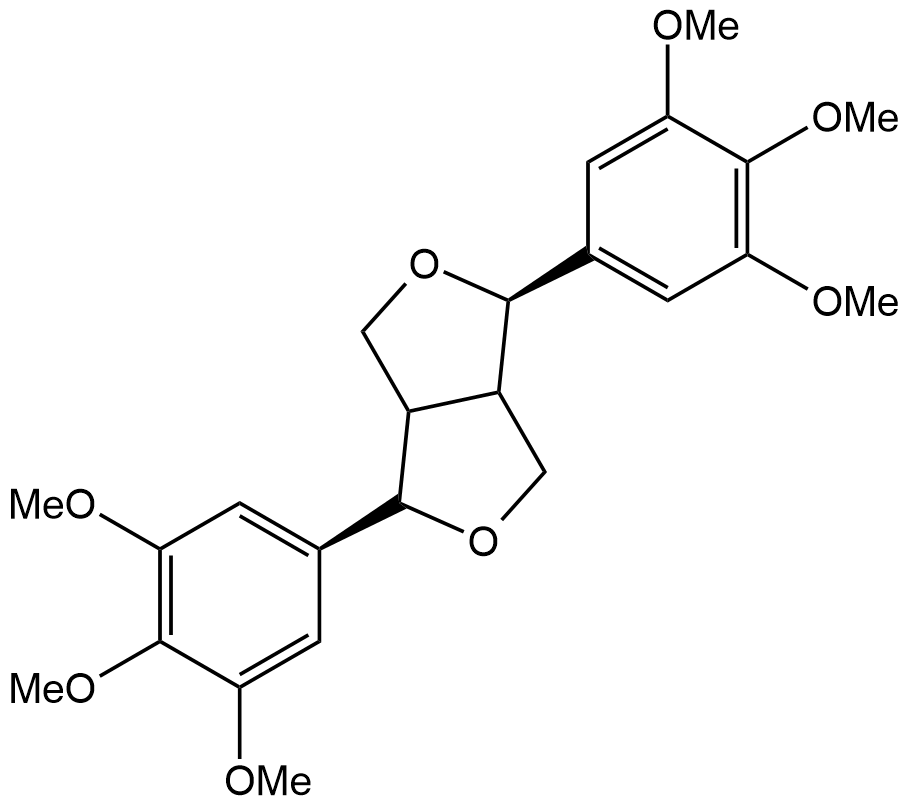

Supplement: Supplementary file 1 [file pharmaceuticals-17-01524-s001.zip › Chemical structure of lignans/(+)-diayangambin.png]

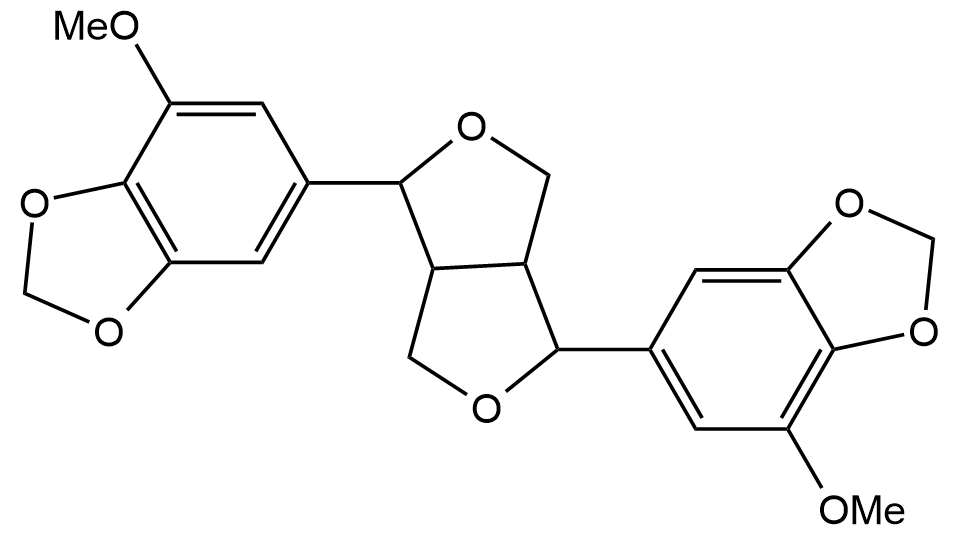

Supplement: Supplementary file 1 [file pharmaceuticals-17-01524-s001.zip › Chemical structure of lignans/5,5'-tetrahydro-1H,3H-furo[3,4-c]furan-1,4-diylbis[7-(methoxy)-1,3-benzodioxole].png]

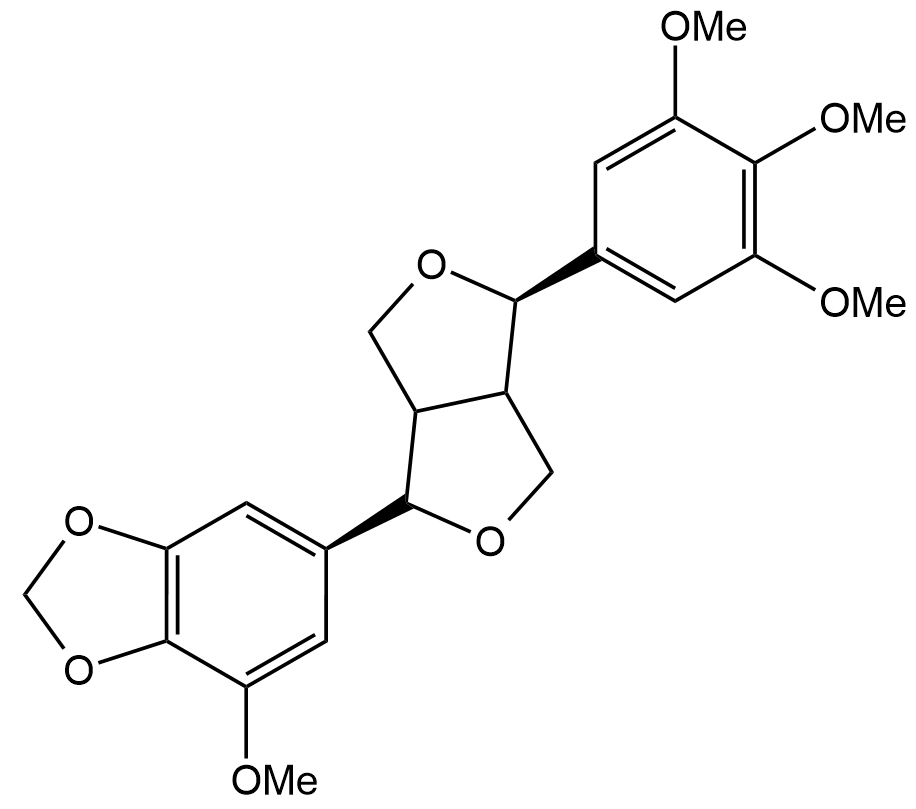

Supplement: Supplementary file 1 [file pharmaceuticals-17-01524-s001.zip › Chemical structure of lignans/diasesartemin.png]

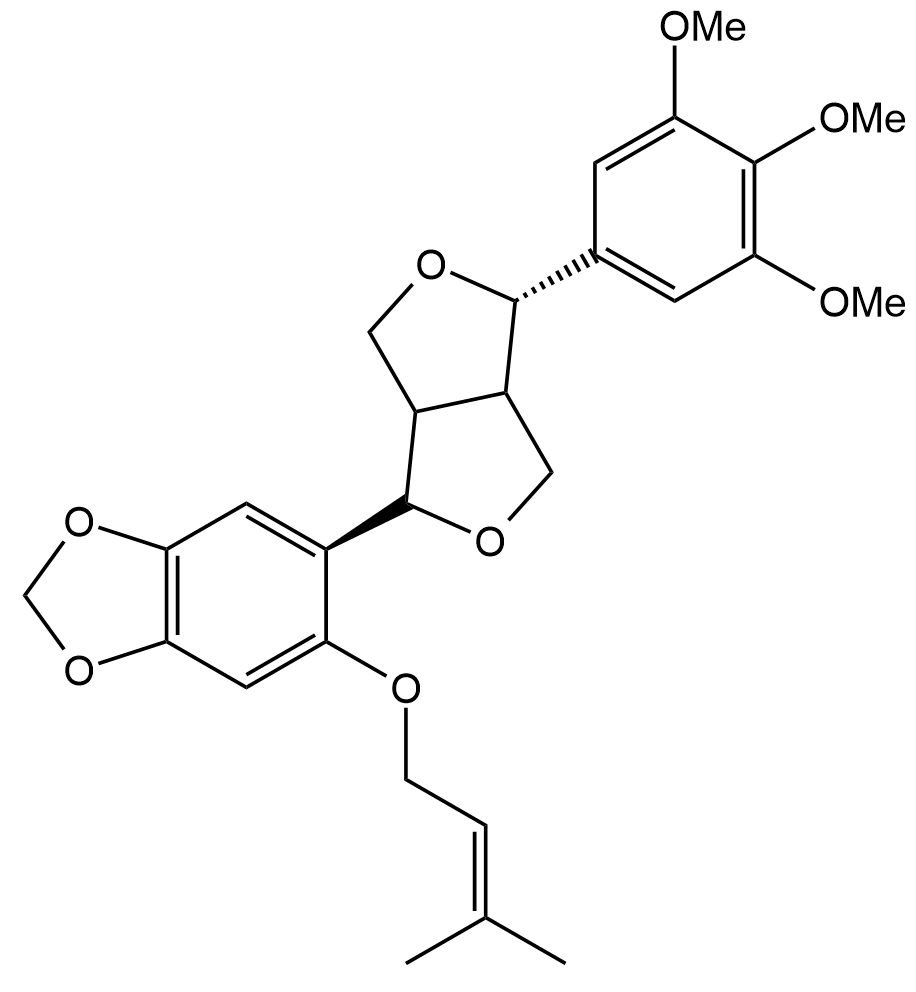

Supplement: Supplementary file 1 [file pharmaceuticals-17-01524-s001.zip › Chemical structure of lignans/epi-mukulin.png]

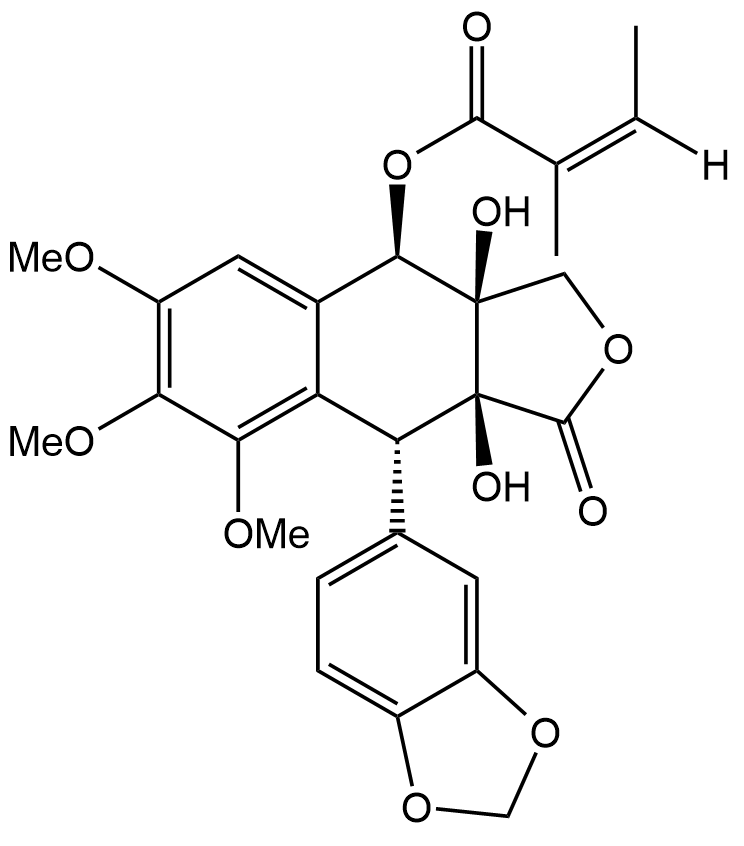

Supplement: Supplementary file 1 [file pharmaceuticals-17-01524-s001.zip › Chemical structure of lignans/erlangerinA.png]

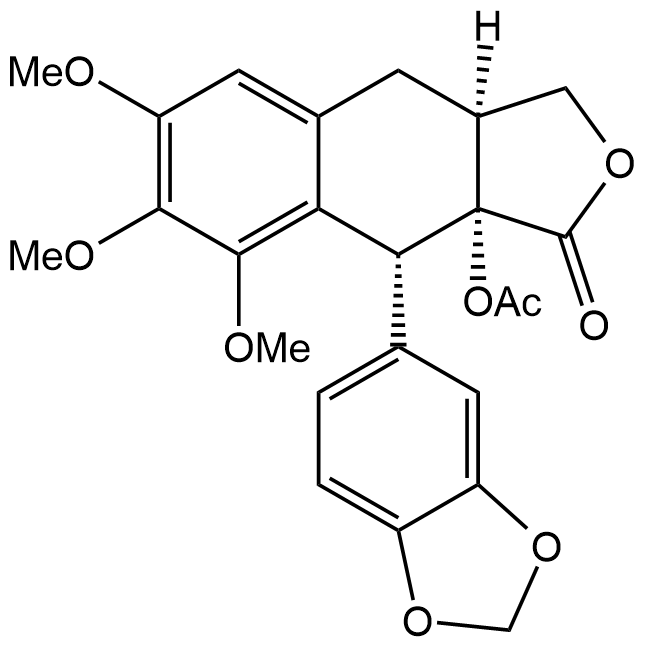

Supplement: Supplementary file 1 [file pharmaceuticals-17-01524-s001.zip › Chemical structure of lignans/erlangerinB.png]

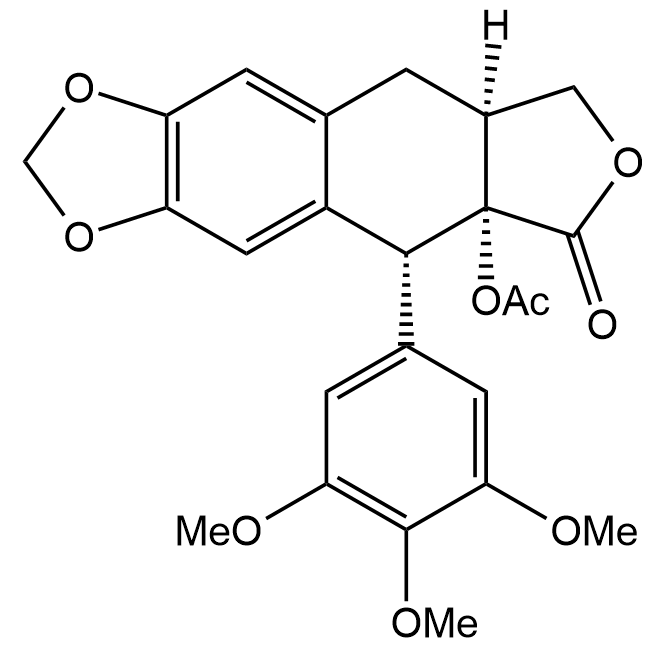

Supplement: Supplementary file 1 [file pharmaceuticals-17-01524-s001.zip › Chemical structure of lignans/erlangerinC.png]

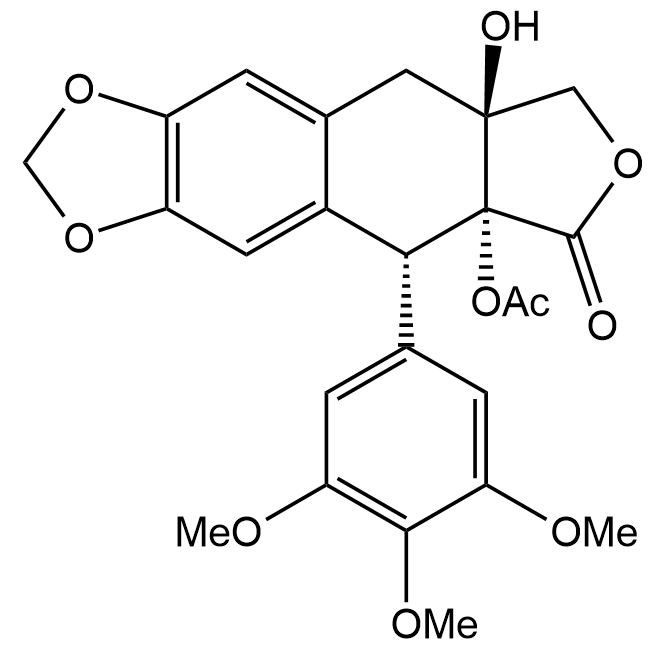

Supplement: Supplementary file 1 [file pharmaceuticals-17-01524-s001.zip › Chemical structure of lignans/erlangerinD.png]

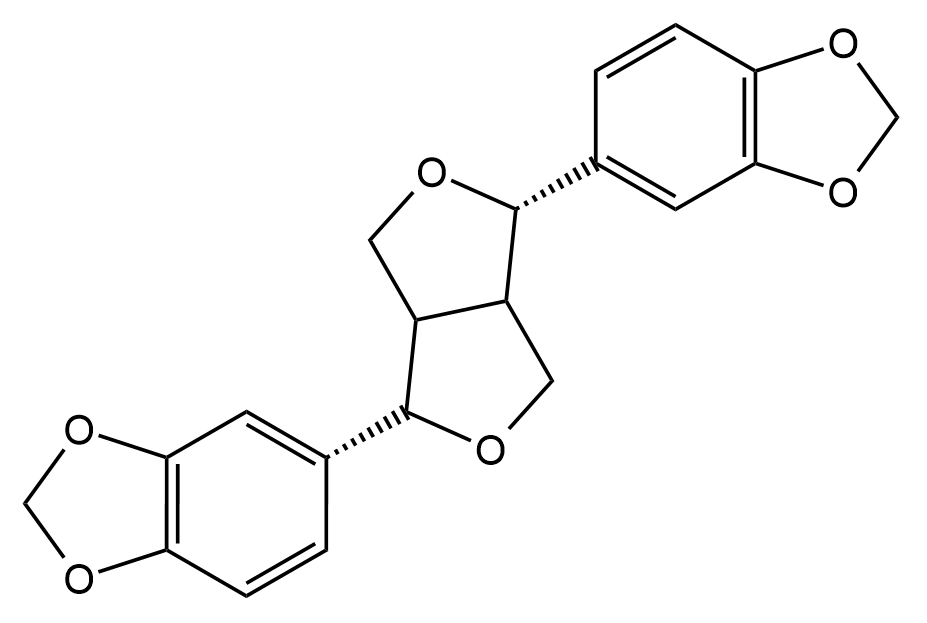

Supplement: Supplementary file 1 [file pharmaceuticals-17-01524-s001.zip › Chemical structure of lignans/sesamin.png]

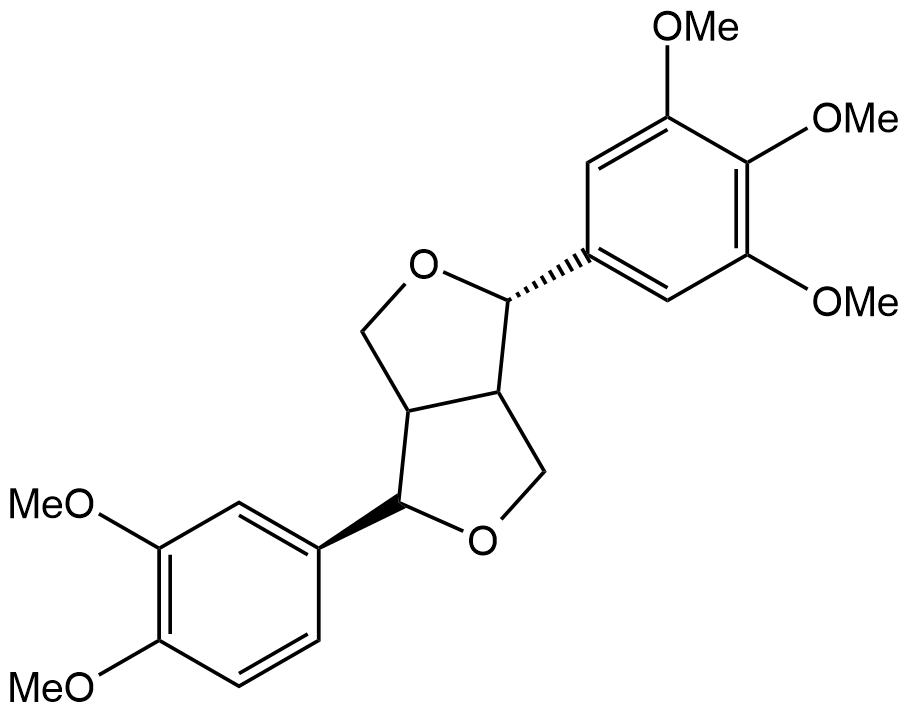

Supplement: Supplementary file 1 [file pharmaceuticals-17-01524-s001.zip › Chemical structure of lignans/(+)-epi-magnolin.png]

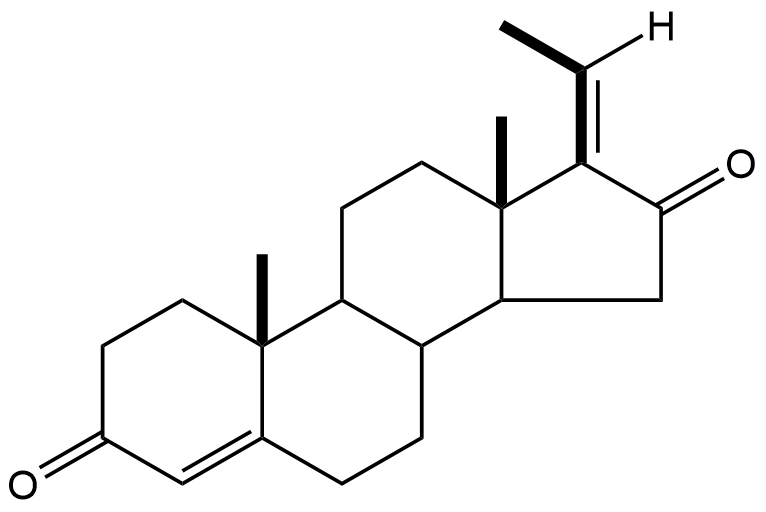

Supplement: Supplementary file 1 [file pharmaceuticals-17-01524-s001.zip › Chemical structure of steroids/(E)-guggulusterone.png]

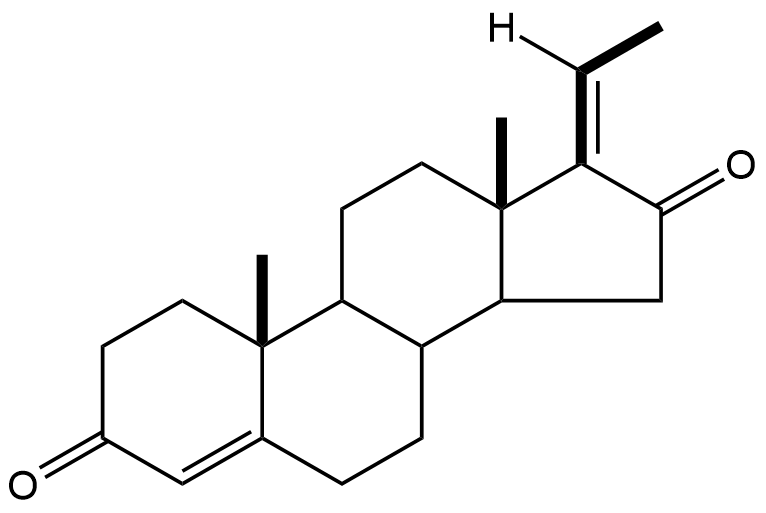

Supplement: Supplementary file 1 [file pharmaceuticals-17-01524-s001.zip › Chemical structure of steroids/(Z)-guggulusterone.png]

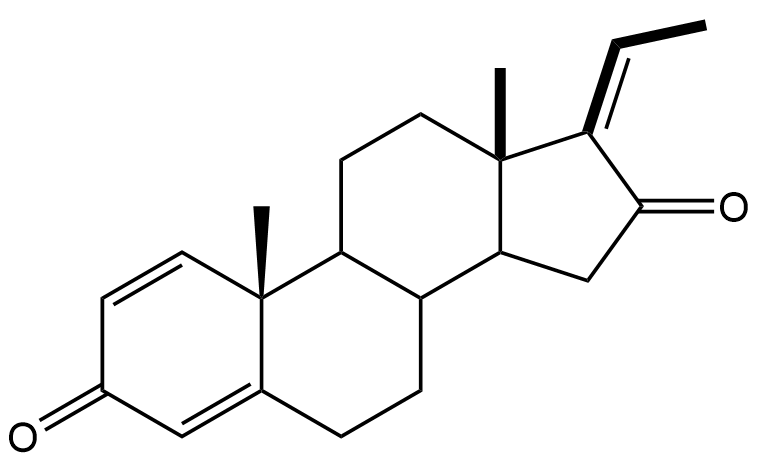

Supplement: Supplementary file 1 [file pharmaceuticals-17-01524-s001.zip › Chemical structure of steroids/(Z)Δ1,2dehydroguggulsterone.png]

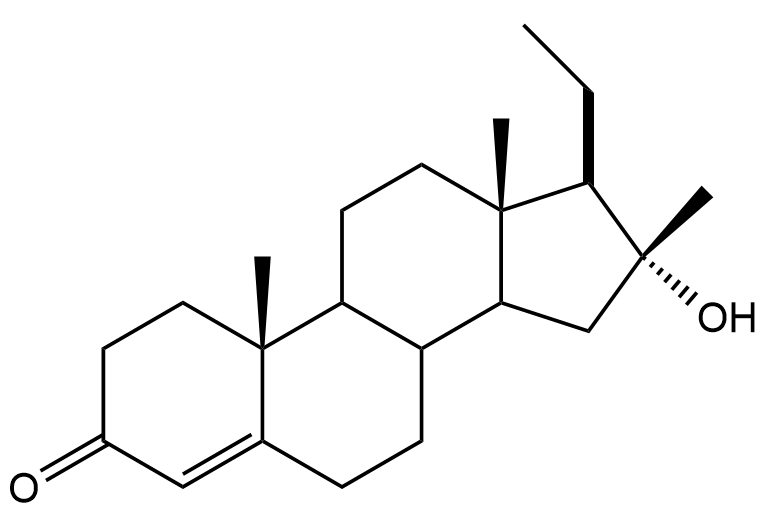

Supplement: Supplementary file 1 [file pharmaceuticals-17-01524-s001.zip › Chemical structure of steroids/16α-hydroxy-4-pregnen-3-one.png]

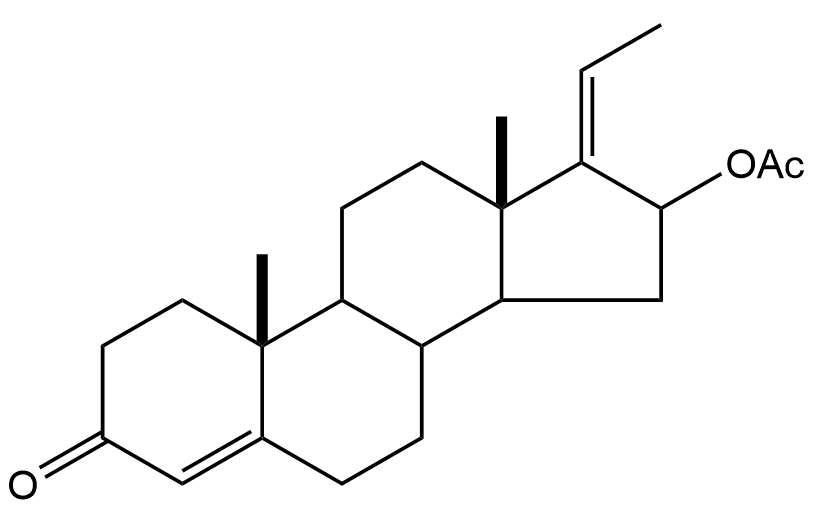

Supplement: Supplementary file 1 [file pharmaceuticals-17-01524-s001.zip › Chemical structure of steroids/16β-acetyloxy-pregn-4,17(20)-trans-dien-3-one.png]

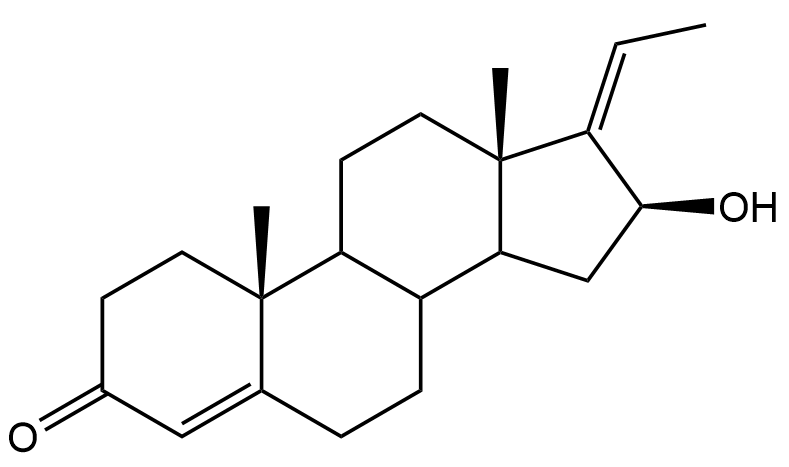

Supplement: Supplementary file 1 [file pharmaceuticals-17-01524-s001.zip › Chemical structure of steroids/16β-hydroxy-4,17(20)Z-pregnadien-3-one.png]

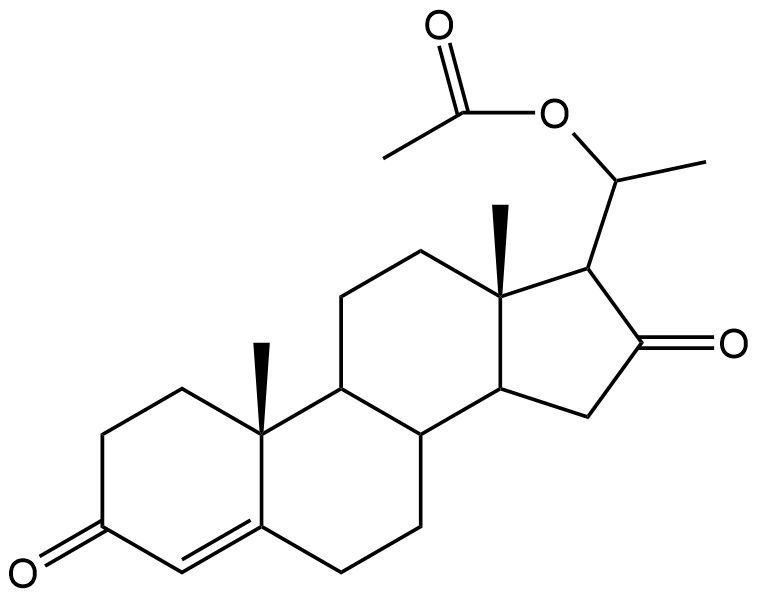

Supplement: Supplementary file 1 [file pharmaceuticals-17-01524-s001.zip › Chemical structure of steroids/20-acetyloxy-4-pregnene-3,16-dione.png]

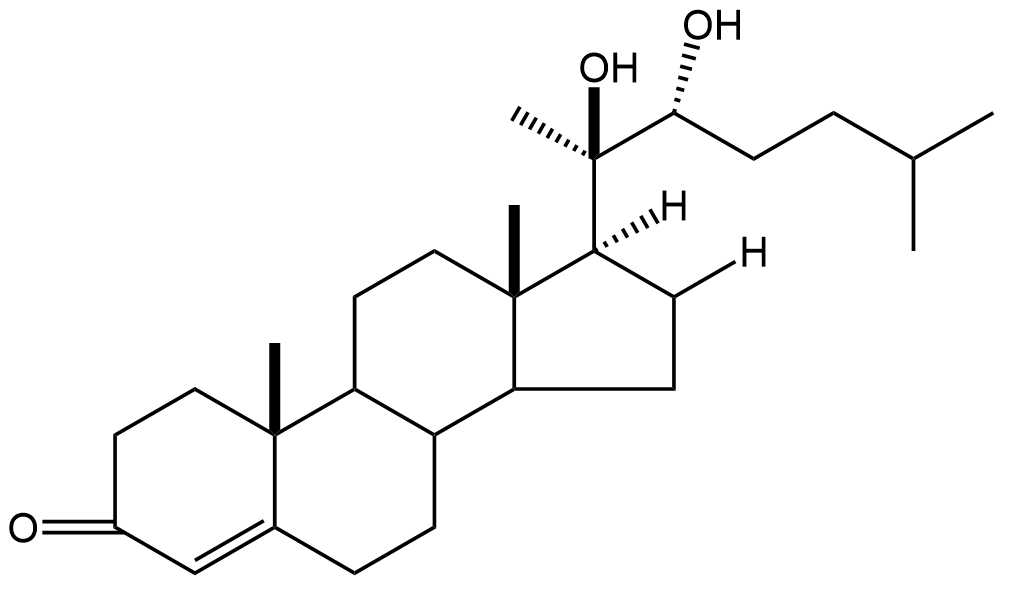

Supplement: Supplementary file 1 [file pharmaceuticals-17-01524-s001.zip › Chemical structure of steroids/20R,22R-dihydroxycholest-4-en-3-one.png]

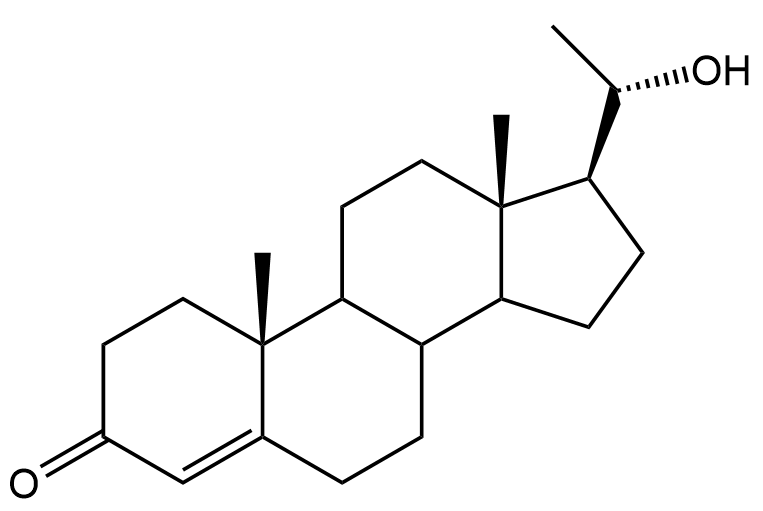

Supplement: Supplementary file 1 [file pharmaceuticals-17-01524-s001.zip › Chemical structure of steroids/20α-hydroxy-4-pregnen-3-one.png]

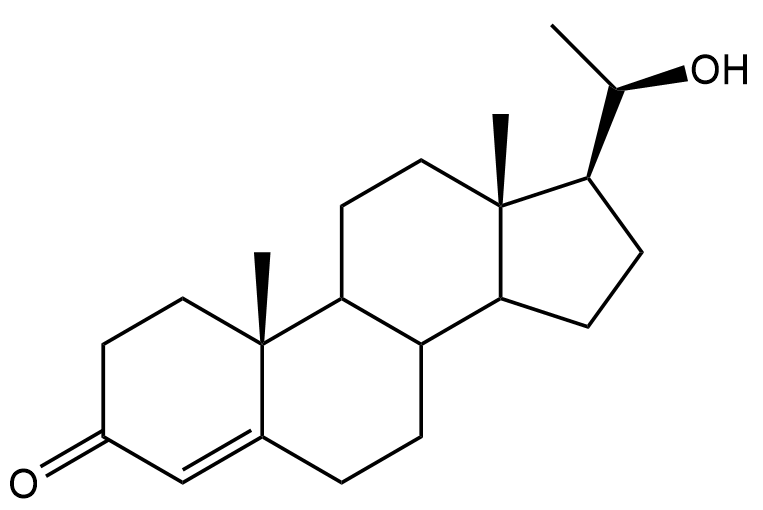

Supplement: Supplementary file 1 [file pharmaceuticals-17-01524-s001.zip › Chemical structure of steroids/20β-hydroxy-4-pregnen-3-one.png]

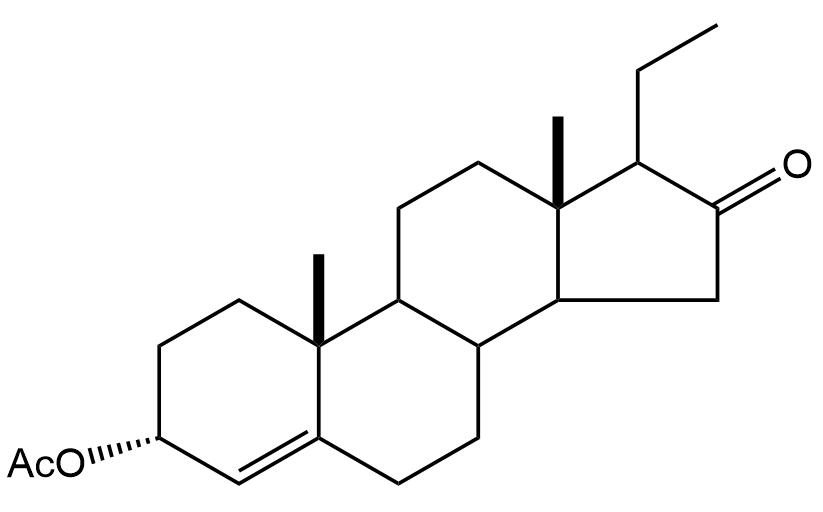

Supplement: Supplementary file 1 [file pharmaceuticals-17-01524-s001.zip › Chemical structure of steroids/3α-acetyloxy-5α-pregnan-16-one.png]

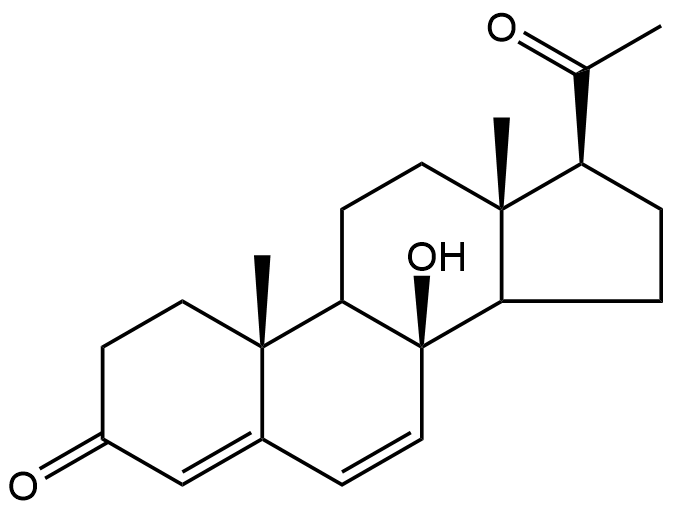

Supplement: Supplementary file 1 [file pharmaceuticals-17-01524-s001.zip › Chemical structure of steroids/8β-hydroxypregnene-4,6-diene-3,20-dione.png]

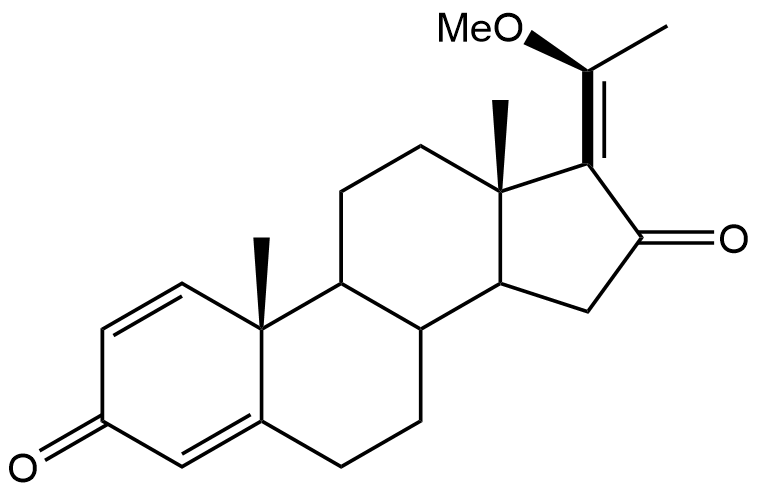

Supplement: Supplementary file 1 [file pharmaceuticals-17-01524-s001.zip › Chemical structure of steroids/dehydroguggulsteroneM.png]

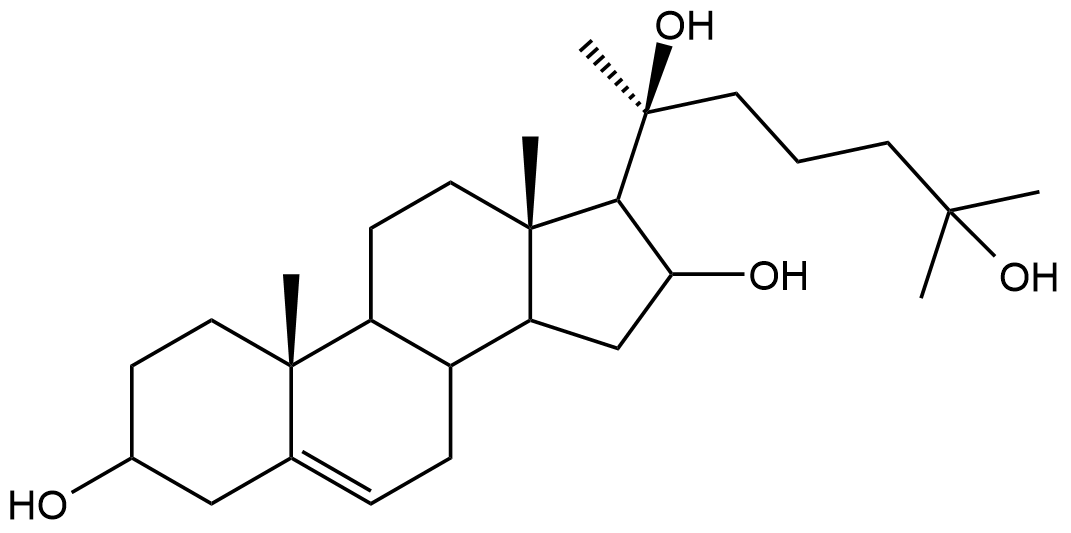

Supplement: Supplementary file 1 [file pharmaceuticals-17-01524-s001.zip › Chemical structure of steroids/guggulsterolY.png]

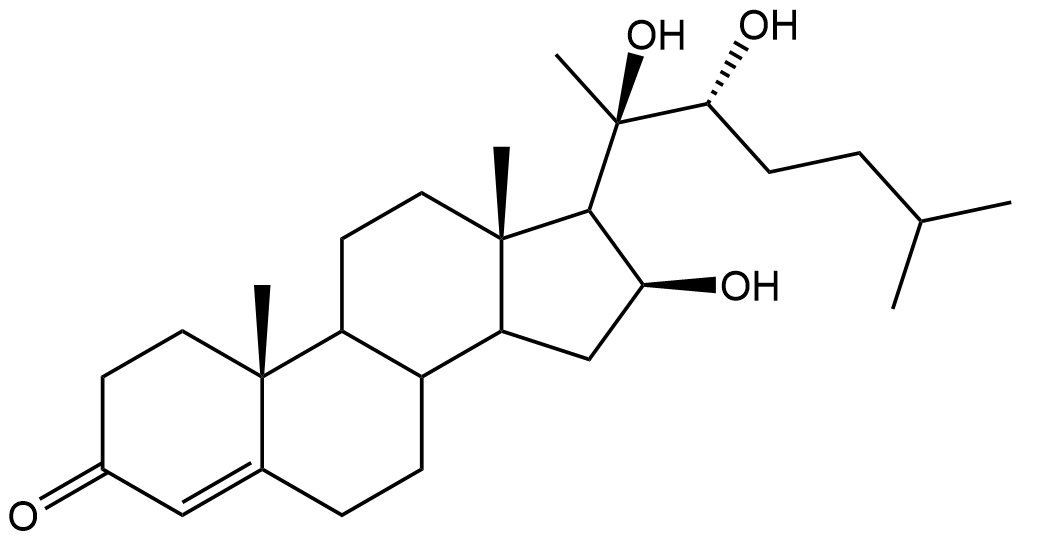

Supplement: Supplementary file 1 [file pharmaceuticals-17-01524-s001.zip › Chemical structure of steroids/guggulsterolI.png]

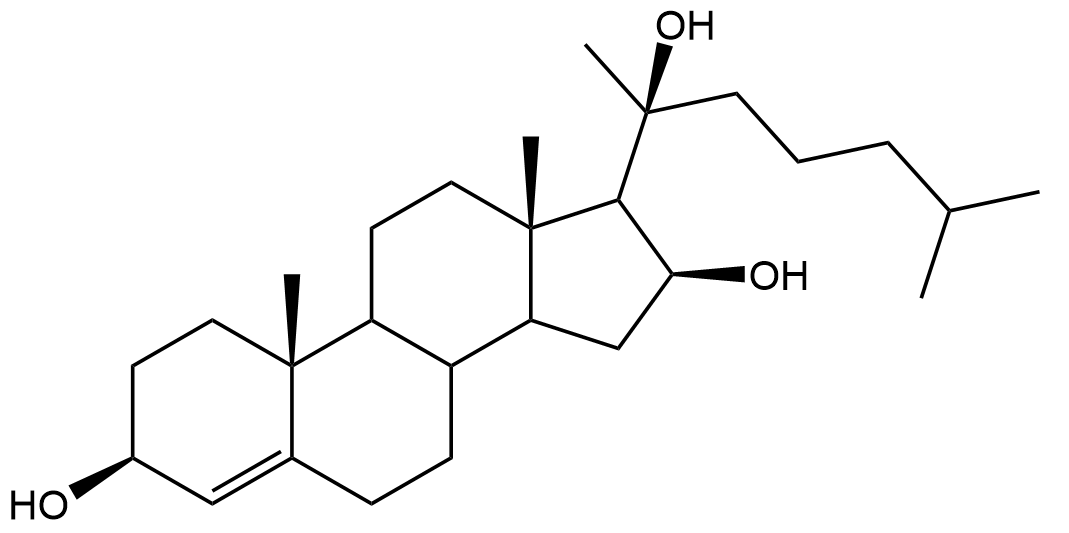

Supplement: Supplementary file 1 [file pharmaceuticals-17-01524-s001.zip › Chemical structure of steroids/guggulsterolII.png]

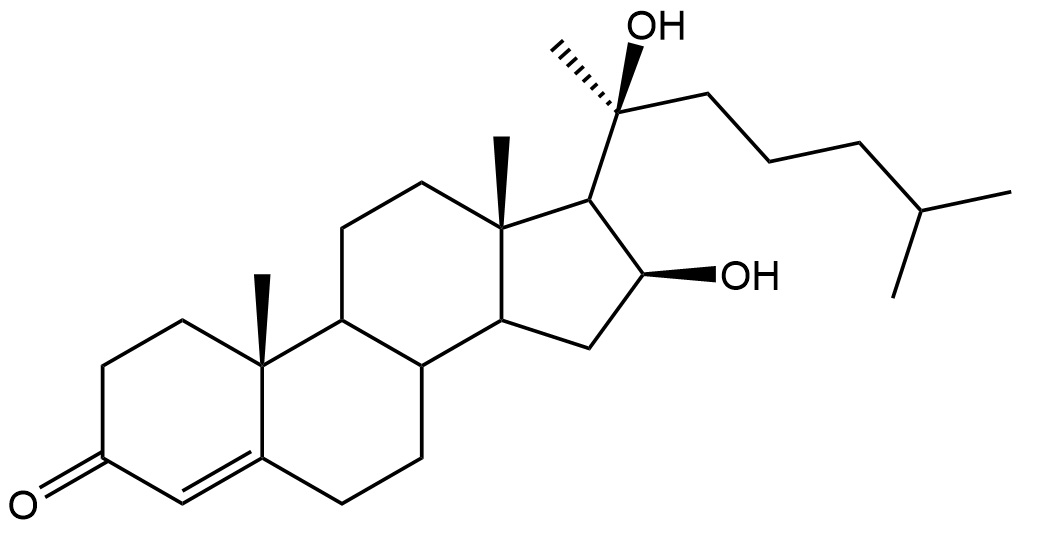

Supplement: Supplementary file 1 [file pharmaceuticals-17-01524-s001.zip › Chemical structure of steroids/guggulsterolIII.png]

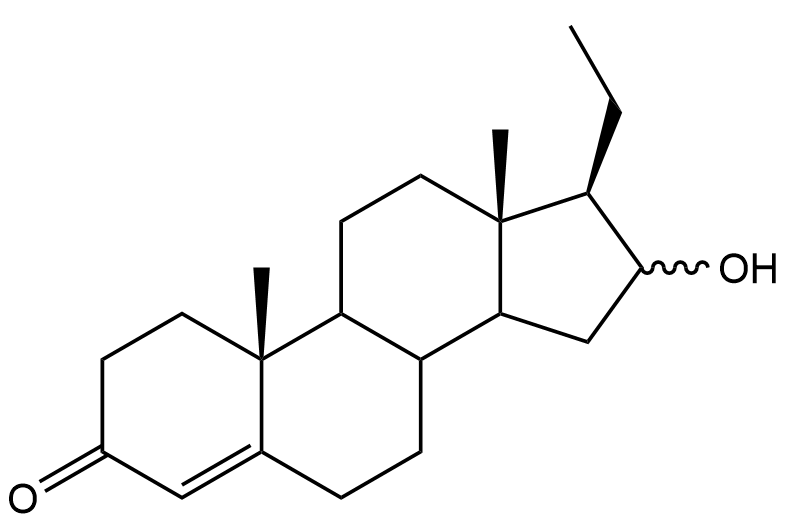

Supplement: Supplementary file 1 [file pharmaceuticals-17-01524-s001.zip › Chemical structure of steroids/guggulsterolIV.png]

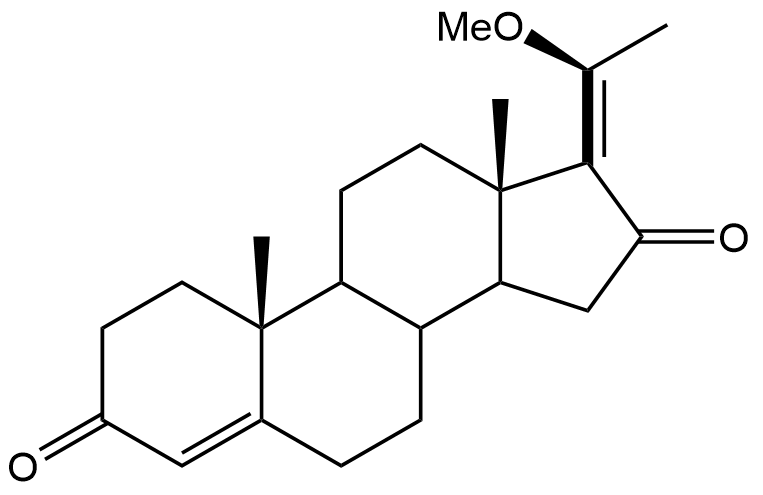

Supplement: Supplementary file 1 [file pharmaceuticals-17-01524-s001.zip › Chemical structure of steroids/guggulsteroneM.png]

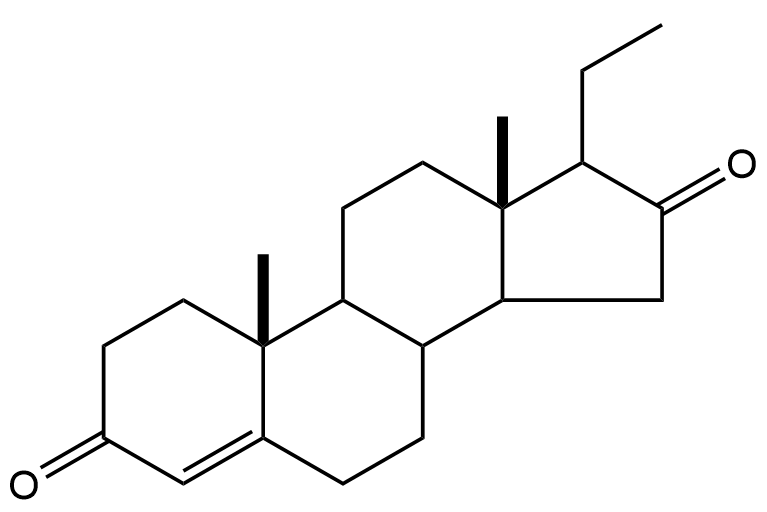

Supplement: Supplementary file 1 [file pharmaceuticals-17-01524-s001.zip › Chemical structure of steroids/pregn-4-ene-3,16-dione.png]

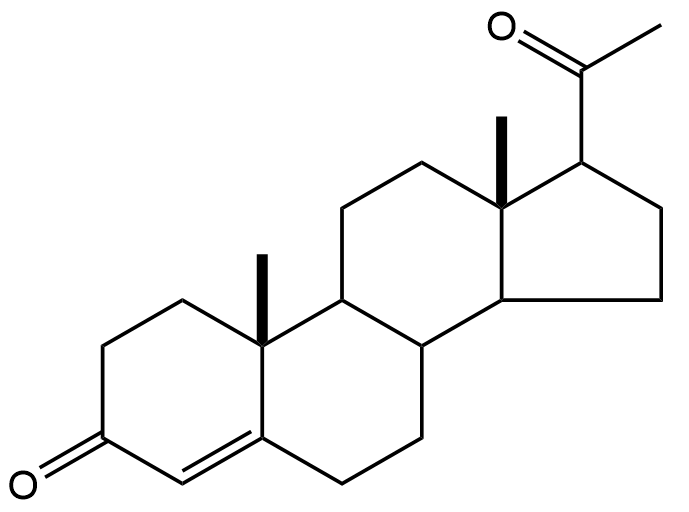

Supplement: Supplementary file 1 [file pharmaceuticals-17-01524-s001.zip › Chemical structure of steroids/progesterone.png]

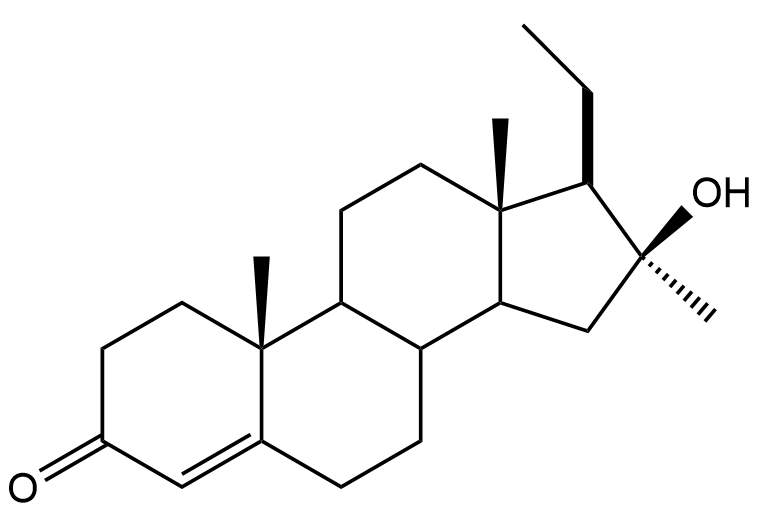

Supplement: Supplementary file 1 [file pharmaceuticals-17-01524-s001.zip › Chemical structure of steroids/β-epimer.png]

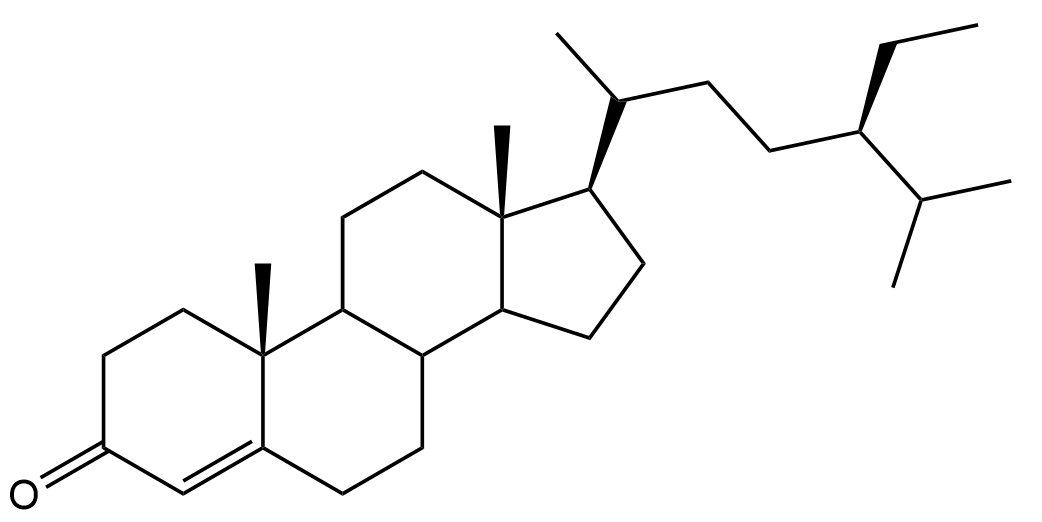

Supplement: Supplementary file 1 [file pharmaceuticals-17-01524-s001.zip › Chemical structure of steroids/β-sitostenone.png]

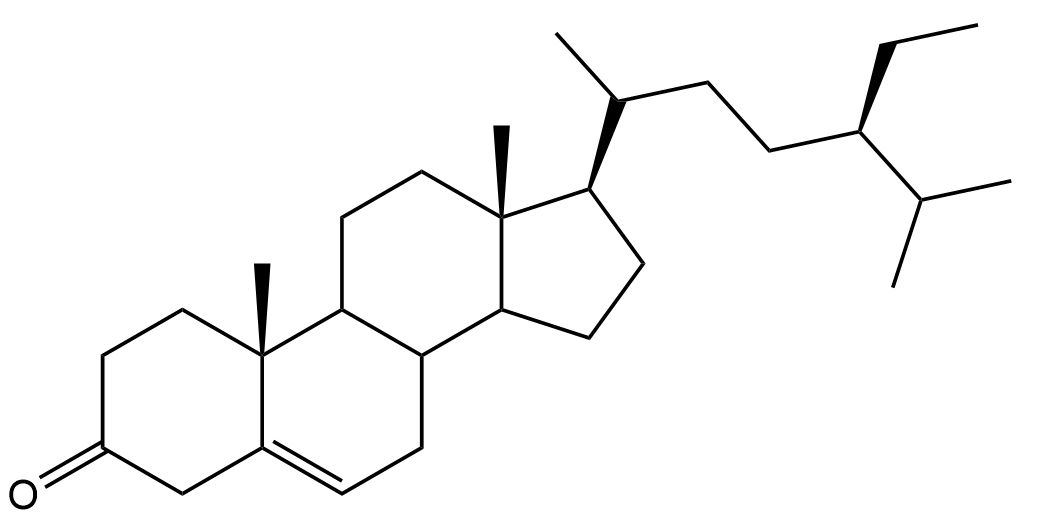

Supplement: Supplementary file 1 [file pharmaceuticals-17-01524-s001.zip › Chemical structure of steroids/β-sitosterol.png]

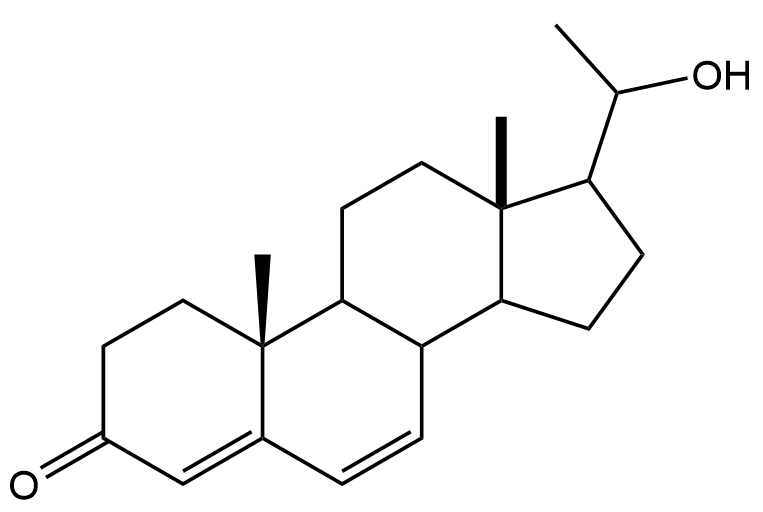

Supplement: Supplementary file 1 [file pharmaceuticals-17-01524-s001.zip › Chemical structure of steroids/Δ6,7dehydro-20-hydroxygugglsterone.png]

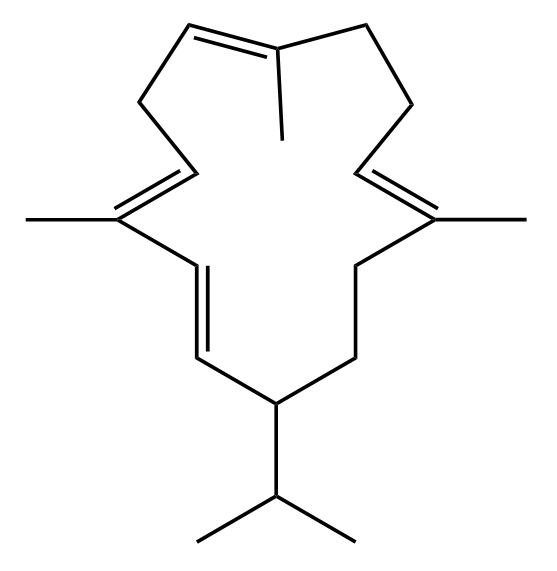

Supplement: Supplementary file 1 [file pharmaceuticals-17-01524-s001.zip › Diterpene chemical structure/(1E,3E,6E,10E)-3,7,11-trimethyl-14-(1-methylethyl)cyclotetradeca-1,3,6,10-tetraene.png]

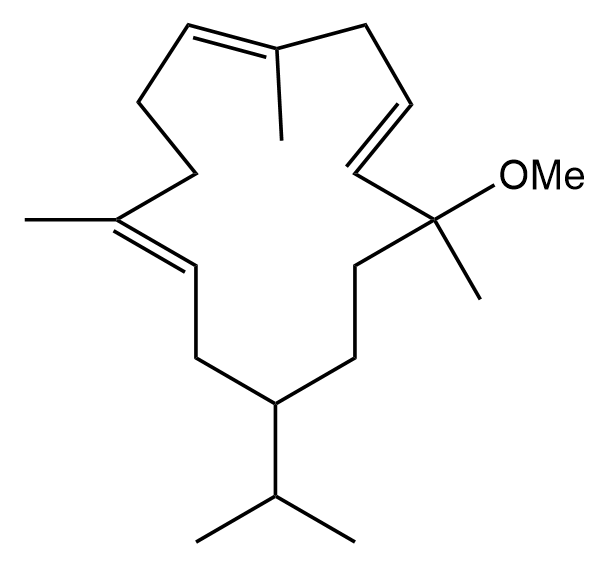

Supplement: Supplementary file 1 [file pharmaceuticals-17-01524-s001.zip › Diterpene chemical structure/(1E,4E,8E)-4,8,14-trimethyl-11-(1-methylethyl)-14-methoxycyclotetradeca-1,4,8-triene.png]

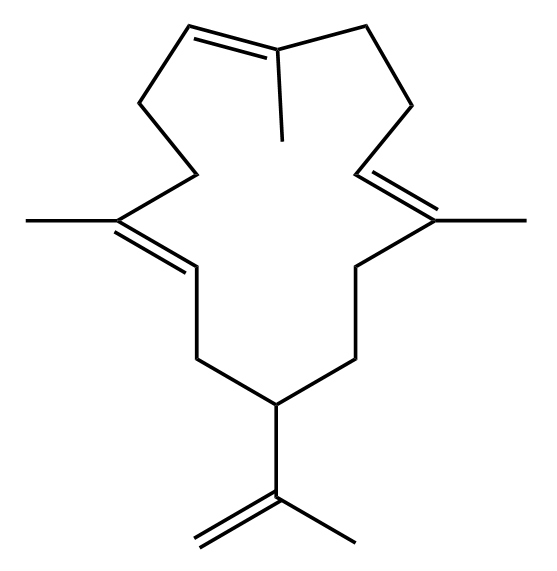

Supplement: Supplementary file 1 [file pharmaceuticals-17-01524-s001.zip › Diterpene chemical structure/(1E,5E,9E)-1,5,9-trimethyl-12-(1-methylethenyl)cyclotetradeca-1,5,9-triene.png]

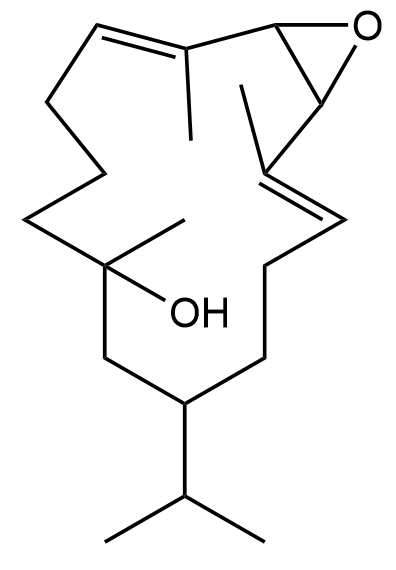

Supplement: Supplementary file 1 [file pharmaceuticals-17-01524-s001.zip › Diterpene chemical structure/(2E,12E)-2,7,13-trimethyl-9-(1-methylethyl)-15-oxabicyclo[12.1.0]pentadeca-2,12-dien-7-ol.png]

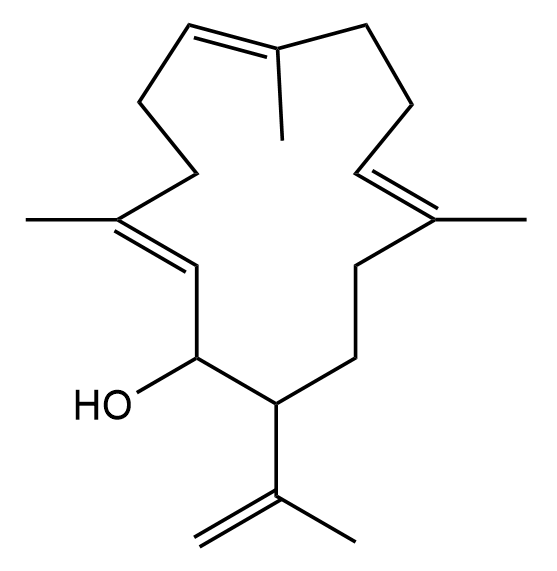

Supplement: Supplementary file 1 [file pharmaceuticals-17-01524-s001.zip › Diterpene chemical structure/(2E,6E,10E)-3,7,11-trimethyl-14-(1-methylethenyl)cyclotetradeca-2,6,10-trien-1-ol.png]

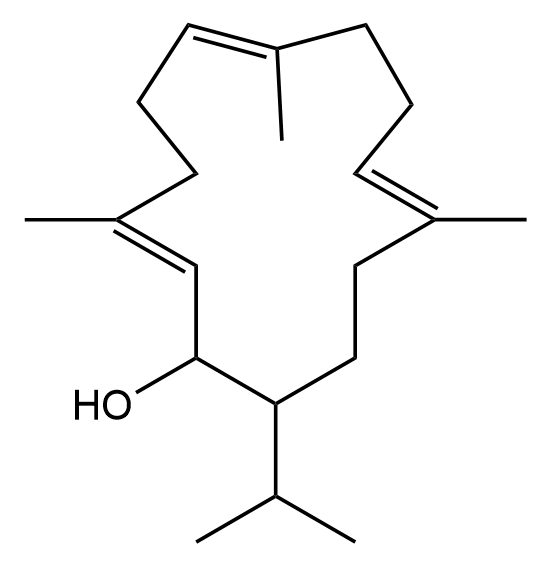

Supplement: Supplementary file 1 [file pharmaceuticals-17-01524-s001.zip › Diterpene chemical structure/(2E,6E,10E)-3,7,11-trimethyl-14-(1-methylethyl)cyclotetradeca-2,6,10-trien-1-ol.png]

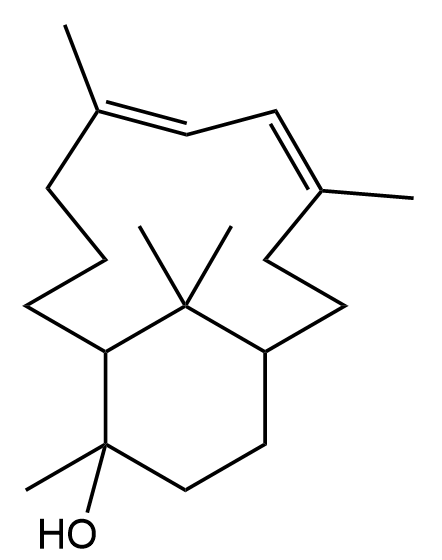

Supplement: Supplementary file 1 [file pharmaceuticals-17-01524-s001.zip › Diterpene chemical structure/(4Z,6E)-4,7,12,15,15-pentamethylbicyclo[9.3.1]pentadeca-4,6-dien-12-ol..png]

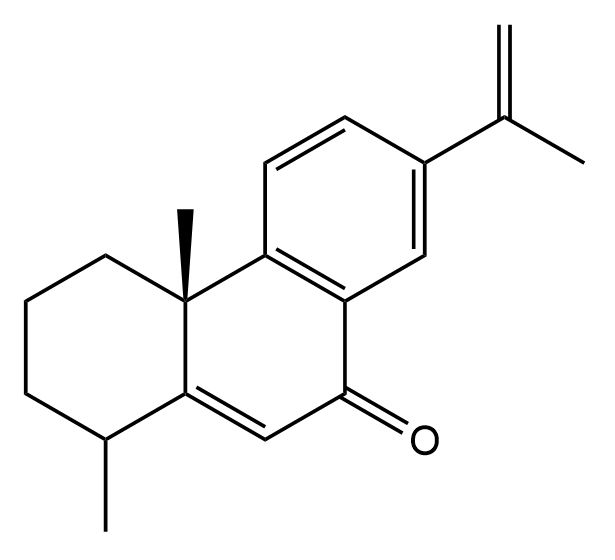

Supplement: Supplementary file 1 [file pharmaceuticals-17-01524-s001.zip › Diterpene chemical structure/19-norabieta-5,8,11,13-tetraen-7-one.png]

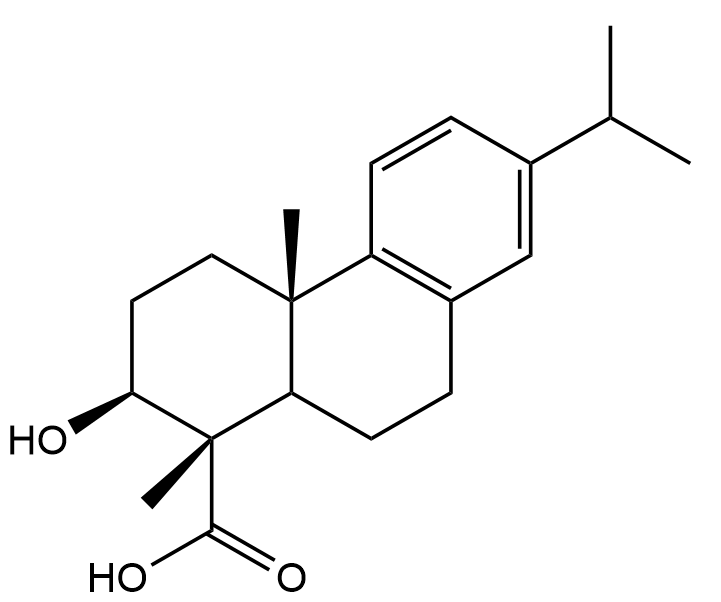

Supplement: Supplementary file 1 [file pharmaceuticals-17-01524-s001.zip › Diterpene chemical structure/3β-hydroxy-dehydroabieticacid.png]

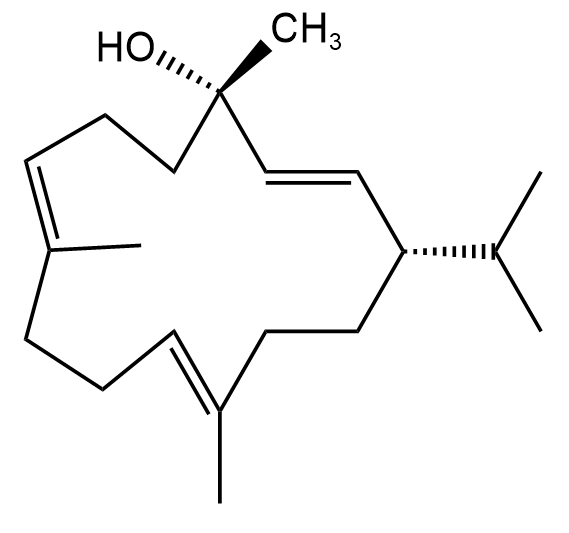

Supplement: Supplementary file 1 [file pharmaceuticals-17-01524-s001.zip › Diterpene chemical structure/4-epiisocembrol.png]

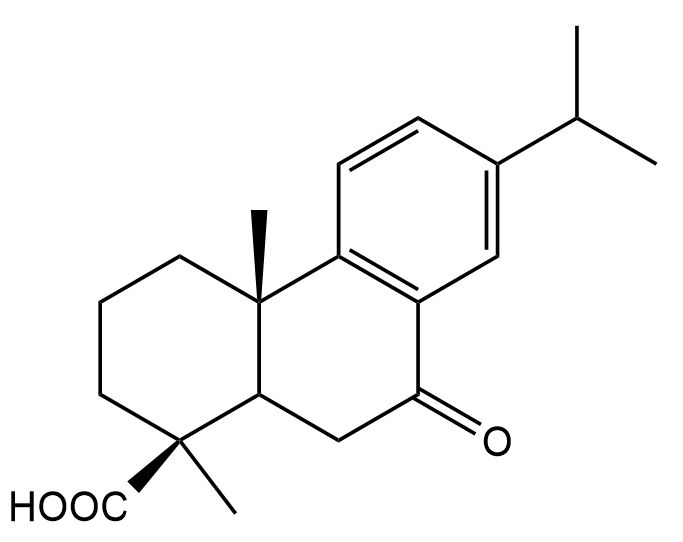

Supplement: Supplementary file 1 [file pharmaceuticals-17-01524-s001.zip › Diterpene chemical structure/7-oxocallitrisicacid.png]

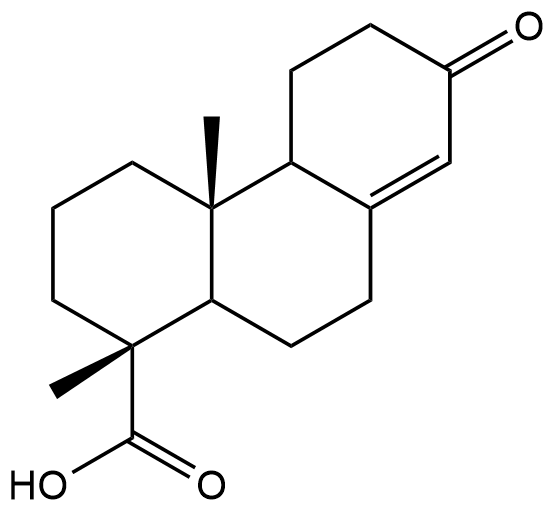

Supplement: Supplementary file 1 [file pharmaceuticals-17-01524-s001.zip › Diterpene chemical structure/8(14)-podocarpen-13-on-18-oicacid.png]

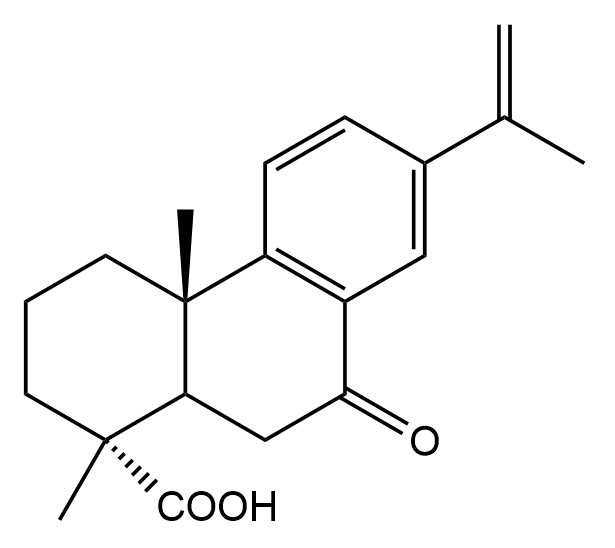

Supplement: Supplementary file 1 [file pharmaceuticals-17-01524-s001.zip › Diterpene chemical structure/abieta-8,11,13,15-tetraen-18-oicacid.png]

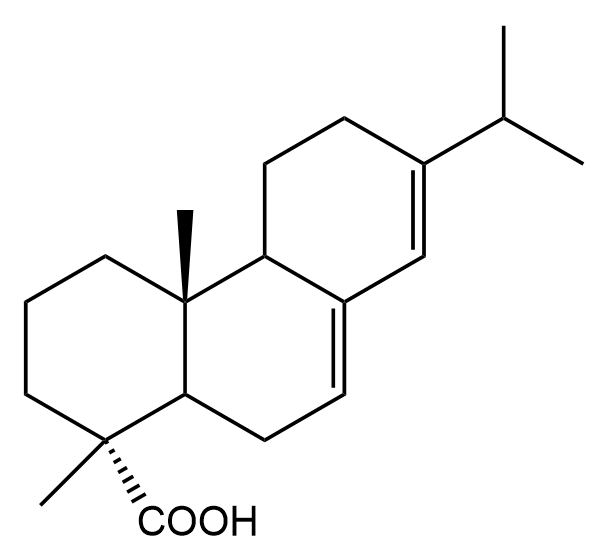

Supplement: Supplementary file 1 [file pharmaceuticals-17-01524-s001.zip › Diterpene chemical structure/abietic acid.png]

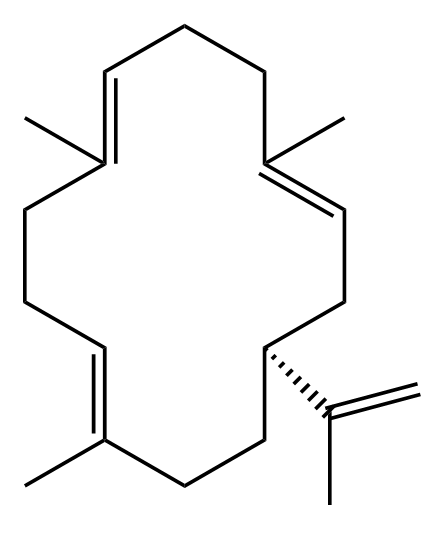

Supplement: Supplementary file 1 [file pharmaceuticals-17-01524-s001.zip › Diterpene chemical structure/cembrene.png]

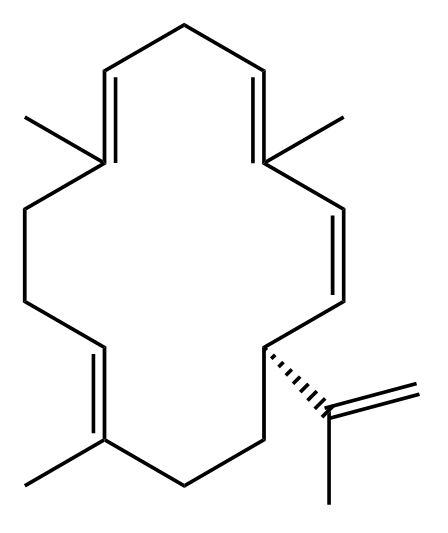

Supplement: Supplementary file 1 [file pharmaceuticals-17-01524-s001.zip › Diterpene chemical structure/cembreneA.png]

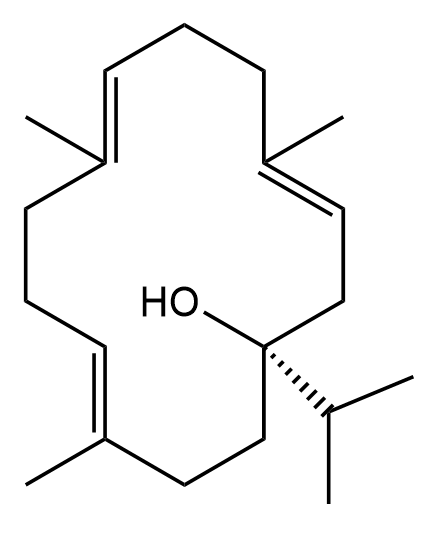

Supplement: Supplementary file 1 [file pharmaceuticals-17-01524-s001.zip › Diterpene chemical structure/cembrenol.png]

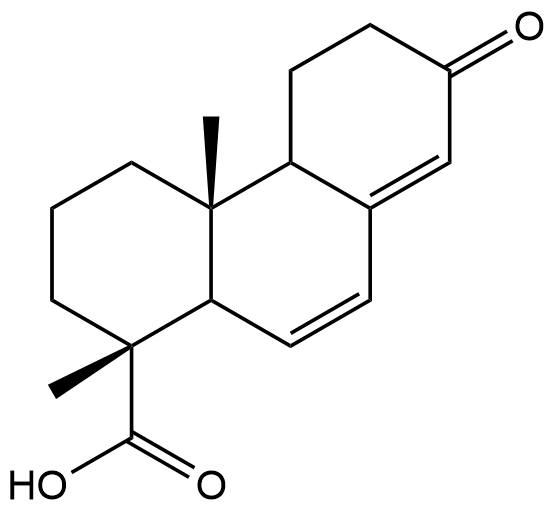

Supplement: Supplementary file 1 [file pharmaceuticals-17-01524-s001.zip › Diterpene chemical structure/commiphorane F.png]

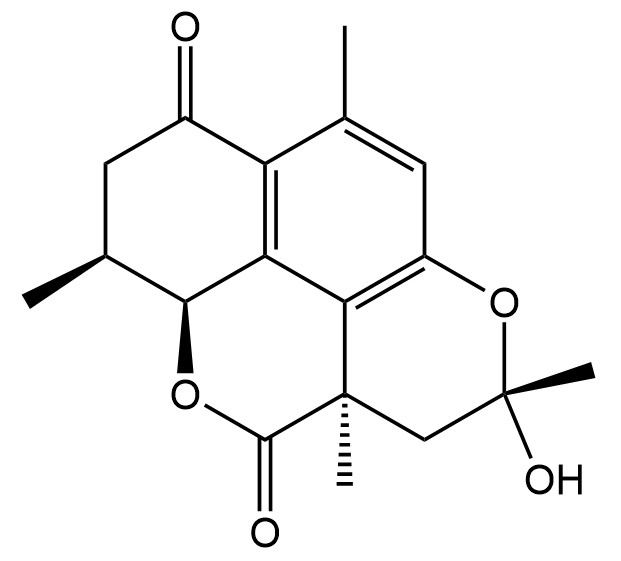

Supplement: Supplementary file 1 [file pharmaceuticals-17-01524-s001.zip › Diterpene chemical structure/commiphoraneA.png]

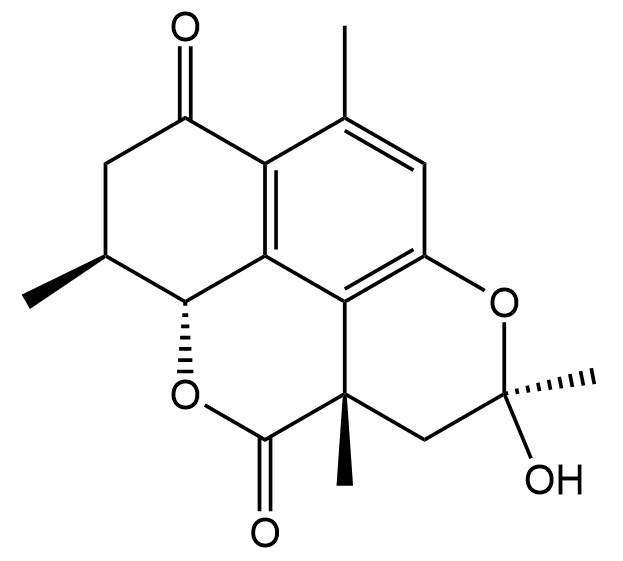

Supplement: Supplementary file 1 [file pharmaceuticals-17-01524-s001.zip › Diterpene chemical structure/commiphoraneB.png]

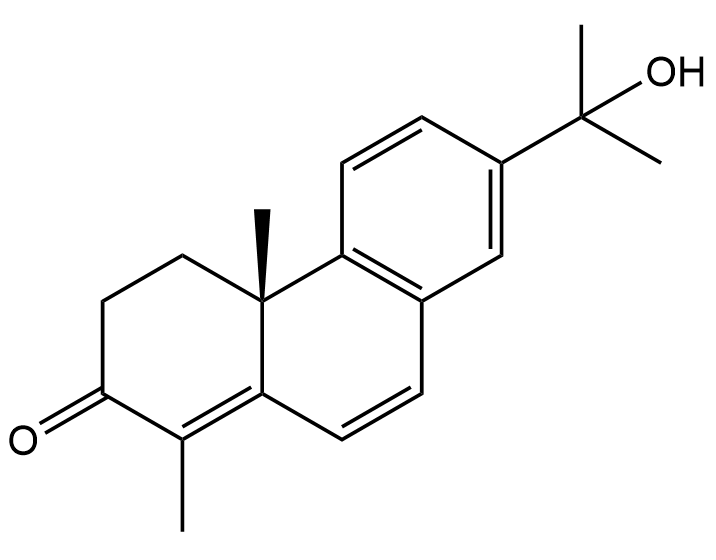

Supplement: Supplementary file 1 [file pharmaceuticals-17-01524-s001.zip › Diterpene chemical structure/commiphoranesK1.png]

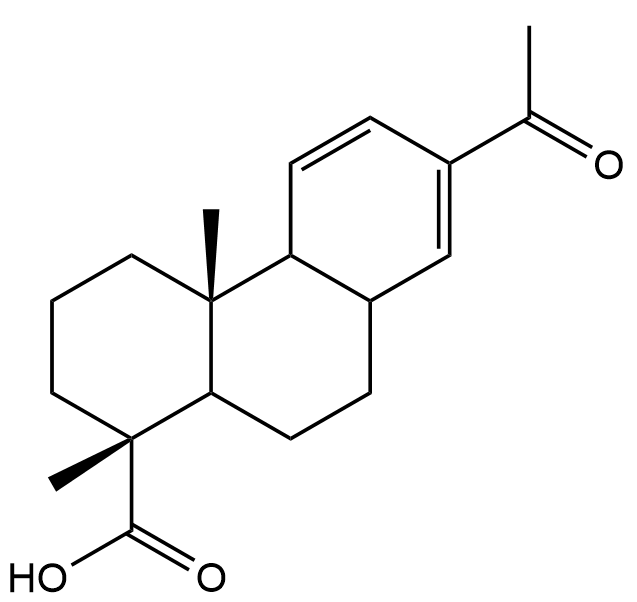

Supplement: Supplementary file 1 [file pharmaceuticals-17-01524-s001.zip › Diterpene chemical structure/commiphoranesK2.png]

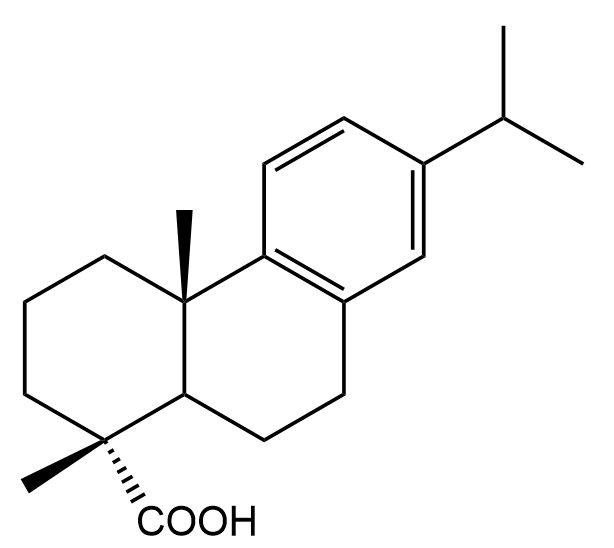

Supplement: Supplementary file 1 [file pharmaceuticals-17-01524-s001.zip › Diterpene chemical structure/dehydroabieticacid.png]

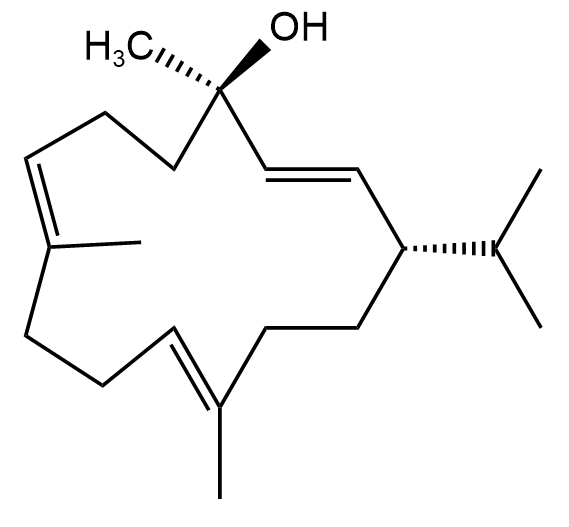

Supplement: Supplementary file 1 [file pharmaceuticals-17-01524-s001.zip › Diterpene chemical structure/isocembrol.png]

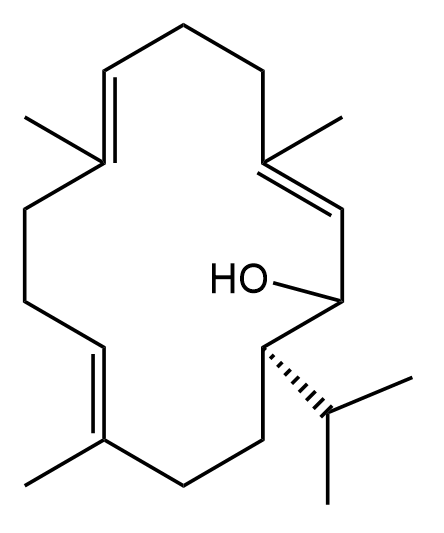

Supplement: Supplementary file 1 [file pharmaceuticals-17-01524-s001.zip › Diterpene chemical structure/mukulol.png]

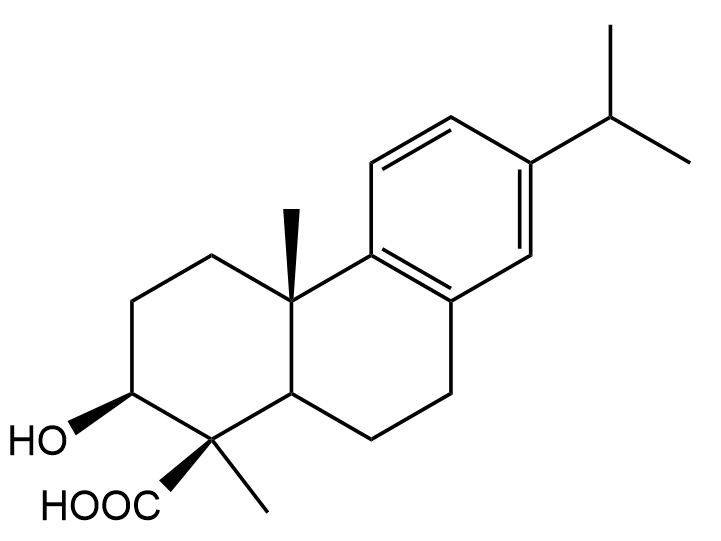

Supplement: Supplementary file 1 [file pharmaceuticals-17-01524-s001.zip › Diterpene chemical structure/nepetaefolinF.png]

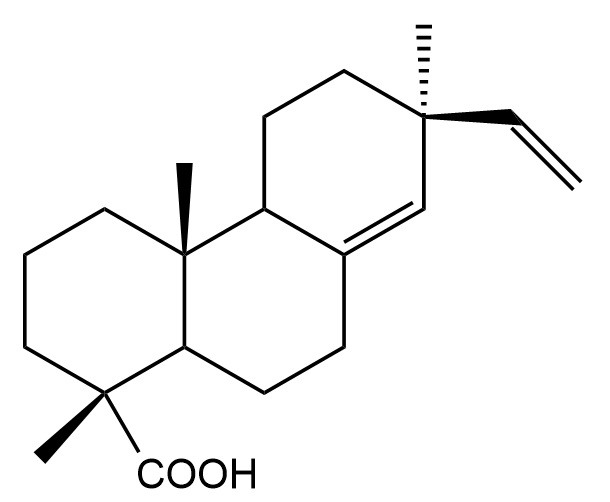

Supplement: Supplementary file 1 [file pharmaceuticals-17-01524-s001.zip › Diterpene chemical structure/pimaricacid.png]

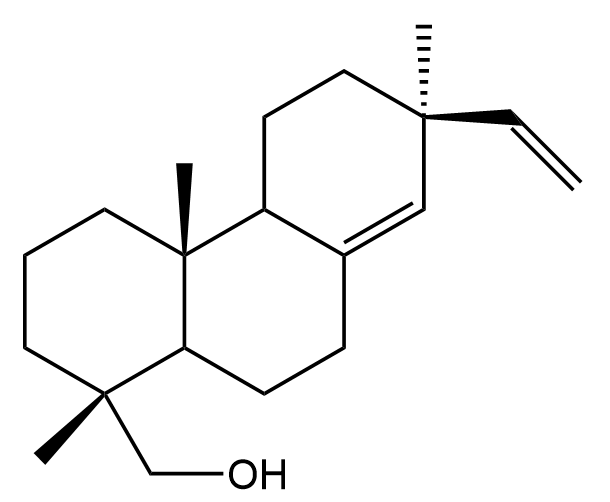

Supplement: Supplementary file 1 [file pharmaceuticals-17-01524-s001.zip › Diterpene chemical structure/pimarol.png]

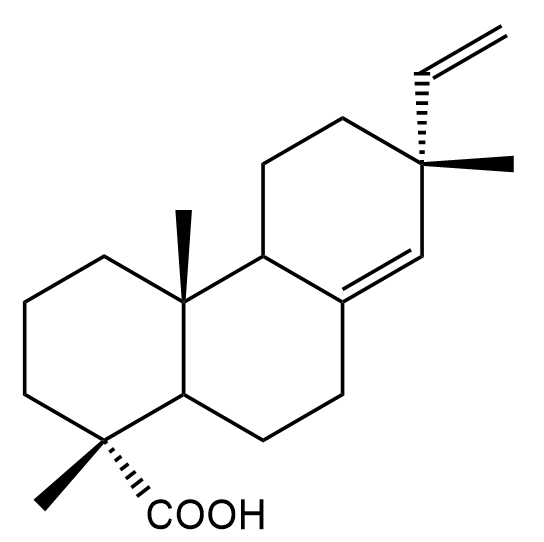

Supplement: Supplementary file 1 [file pharmaceuticals-17-01524-s001.zip › Diterpene chemical structure/sandaracopimaric acid.png]

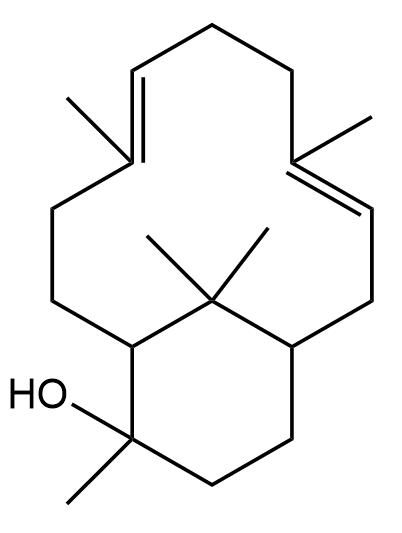

Supplement: Supplementary file 1 [file pharmaceuticals-17-01524-s001.zip › Diterpene chemical structure/verticillol.png]

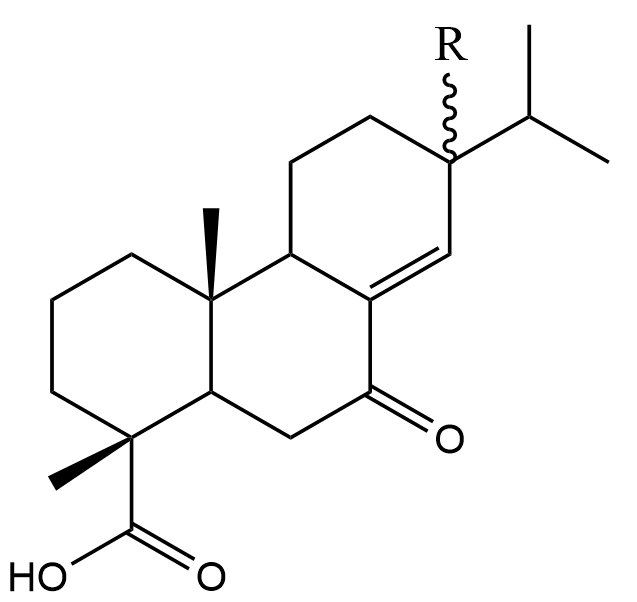

Supplement: Supplementary file 1 [file pharmaceuticals-17-01524-s001.zip › Diterpene chemical structure/前四个.png]

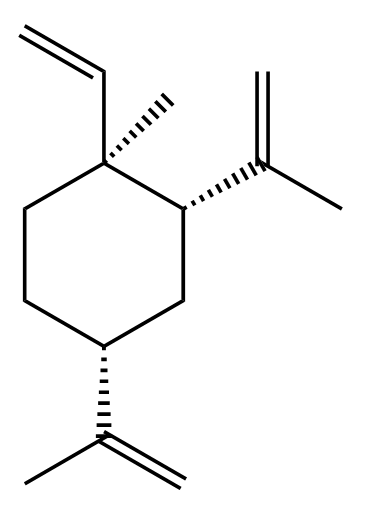

Supplement: Supplementary file 1 [file pharmaceuticals-17-01524-s001.zip › Monoterpene chemical structure/(-)-β-elemene.png]

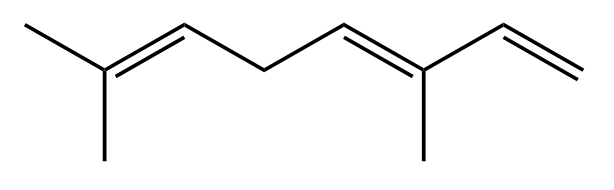

Supplement: Supplementary file 1 [file pharmaceuticals-17-01524-s001.zip › Monoterpene chemical structure/3,7-dimethylocta-1,3,7-triene.png]

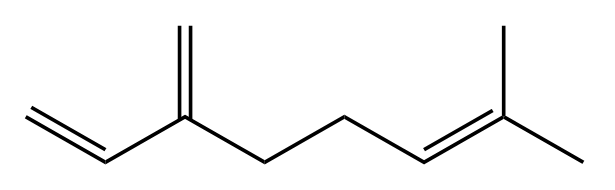

Supplement: Supplementary file 1 [file pharmaceuticals-17-01524-s001.zip › Monoterpene chemical structure/7-Methyl-3-methylene-1,6-octadiene.png]

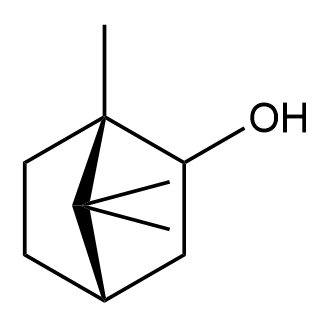

Supplement: Supplementary file 1 [file pharmaceuticals-17-01524-s001.zip › Monoterpene chemical structure/Borneol.png]

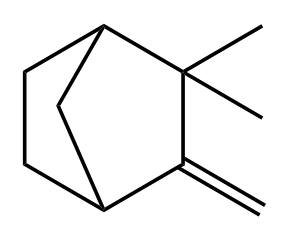

Supplement: Supplementary file 1 [file pharmaceuticals-17-01524-s001.zip › Monoterpene chemical structure/Camphene.png]

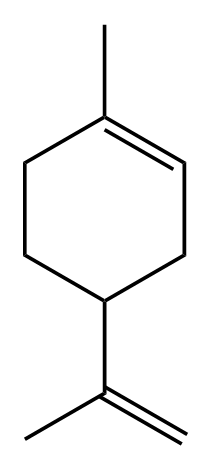

Supplement: Supplementary file 1 [file pharmaceuticals-17-01524-s001.zip › Monoterpene chemical structure/Cinene.png]

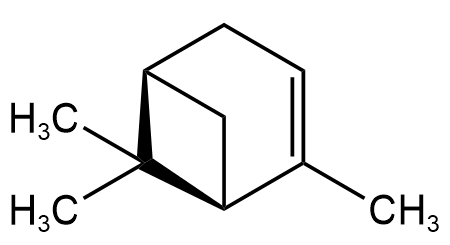

Supplement: Supplementary file 1 [file pharmaceuticals-17-01524-s001.zip › Monoterpene chemical structure/α-Pinene.png]

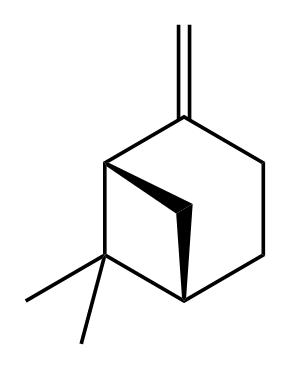

Supplement: Supplementary file 1 [file pharmaceuticals-17-01524-s001.zip › Monoterpene chemical structure/β-Pinene.png]

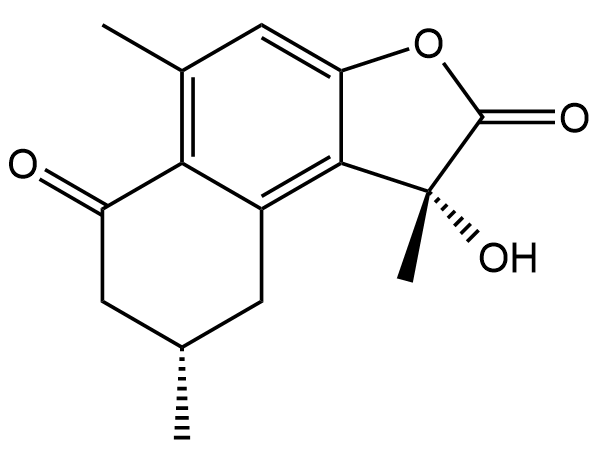

Supplement: Supplementary file 1 [file pharmaceuticals-17-01524-s001.zip › Sesquiterpene chemical structure/(+)-myrrhalactoneA.png]

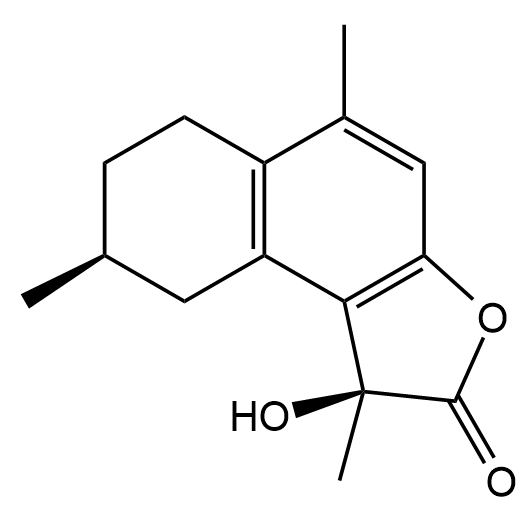

Supplement: Supplementary file 1 [file pharmaceuticals-17-01524-s001.zip › Sesquiterpene chemical structure/(11β)-8,11-dihydroxy-cadina-6,8,10-trien-12-oicacid-γ.png]

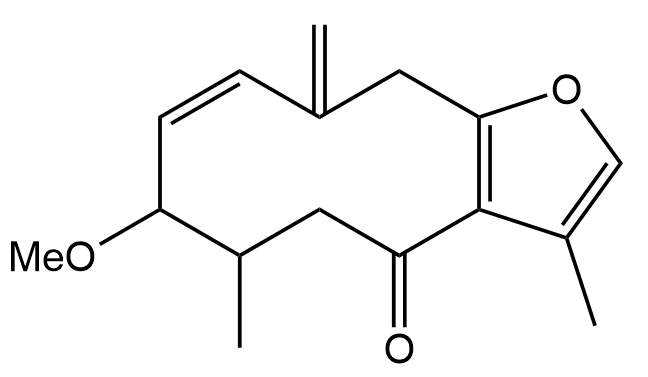

Supplement: Supplementary file 1 [file pharmaceuticals-17-01524-s001.zip › Sesquiterpene chemical structure/(1E)-3-methoxy-8,12-epoxygermacra-1,7,10,11-tetraen-6.png]

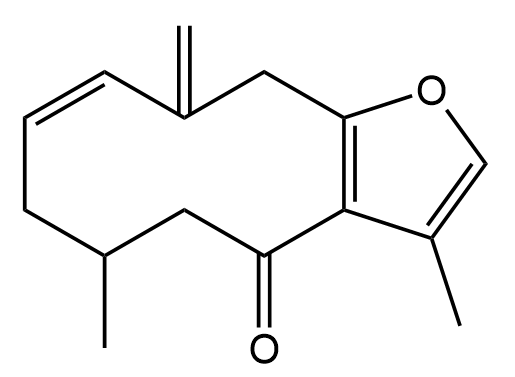

Supplement: Supplementary file 1 [file pharmaceuticals-17-01524-s001.zip › Sesquiterpene chemical structure/(1E)-8,12epoxygermacra-1,7,10,11-tetraen-6-one furanogermacra-1E,10(15)-dien-6-one.png]

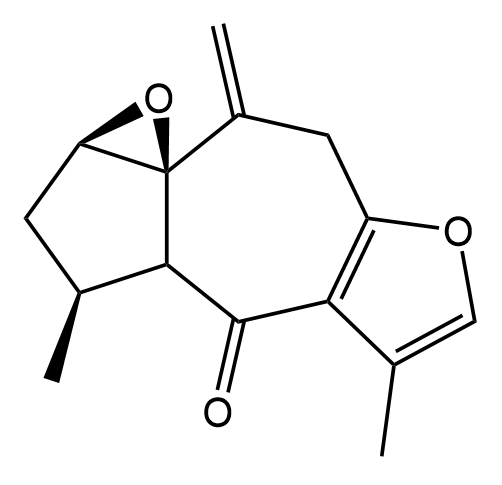

Supplement: Supplementary file 1 [file pharmaceuticals-17-01524-s001.zip › Sesquiterpene chemical structure/(1R,2R,4S)-1,2-epoxyfuranogermacr-10(15)-en-6-one.png]

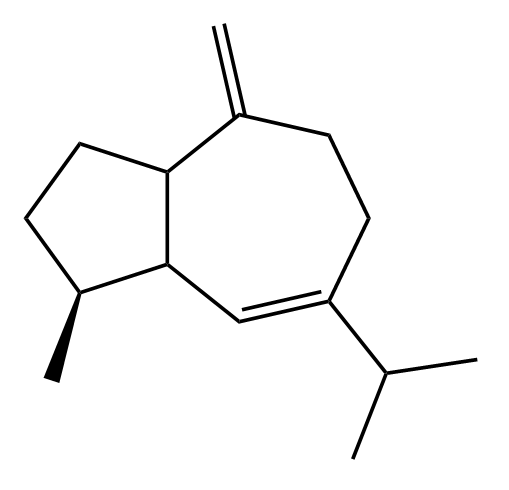

Supplement: Supplementary file 1 [file pharmaceuticals-17-01524-s001.zip › Sesquiterpene chemical structure/(1R,4S,5R)-guaia-6,10(14)-diene.png]

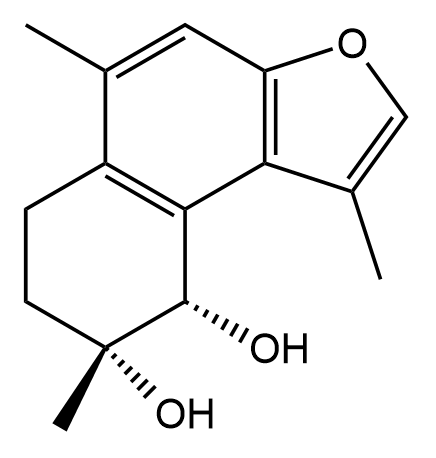

Supplement: Supplementary file 1 [file pharmaceuticals-17-01524-s001.zip › Sesquiterpene chemical structure/(±)-commyrrinA.png]

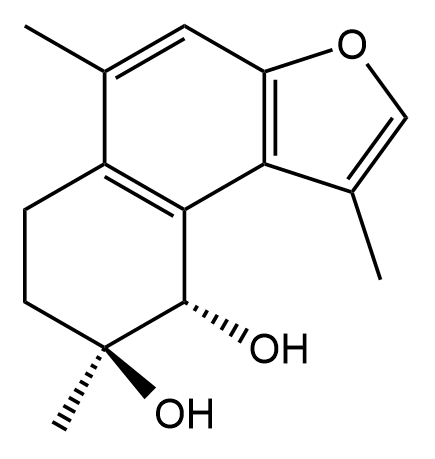

Supplement: Supplementary file 1 [file pharmaceuticals-17-01524-s001.zip › Sesquiterpene chemical structure/(±)-commyrrinB.png]

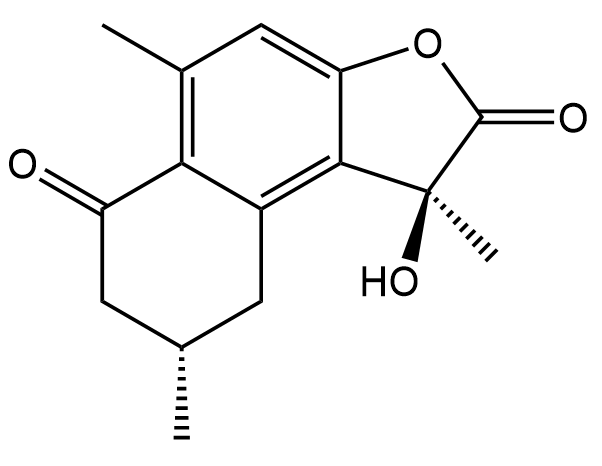

Supplement: Supplementary file 1 [file pharmaceuticals-17-01524-s001.zip › Sesquiterpene chemical structure/(–)-myrrhalactoneA.png]

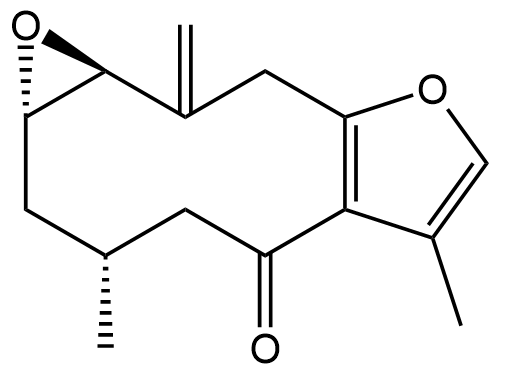

Supplement: Supplementary file 1 [file pharmaceuticals-17-01524-s001.zip › Sesquiterpene chemical structure/1,2-epoxyfurano-l0(15)-germamen-6-one.png]

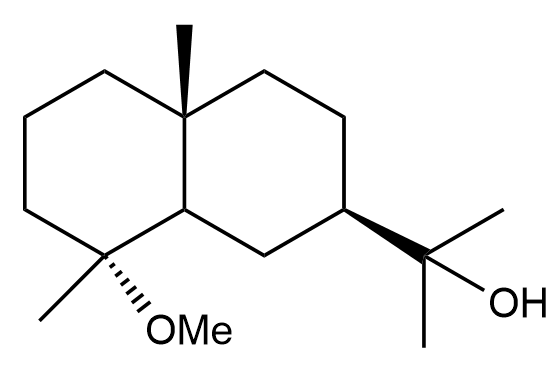

Supplement: Supplementary file 1 [file pharmaceuticals-17-01524-s001.zip › Sesquiterpene chemical structure/11-hydroxy-4α-methoxy-selinane.png]

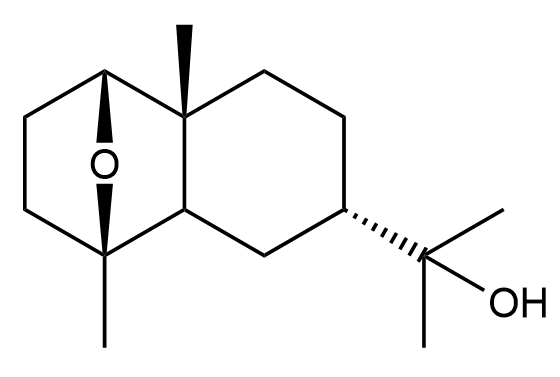

Supplement: Supplementary file 1 [file pharmaceuticals-17-01524-s001.zip › Sesquiterpene chemical structure/1β,4β-epoxy-eudesmane-11-ol.png]

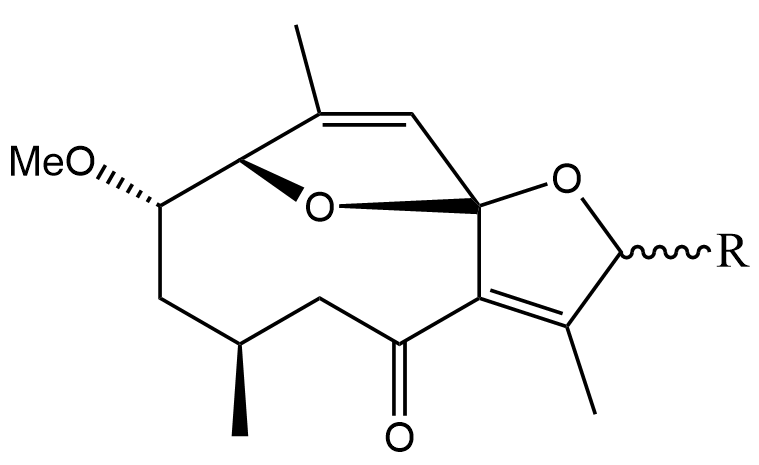

Supplement: Supplementary file 1 [file pharmaceuticals-17-01524-s001.zip › Sesquiterpene chemical structure/1β,8β-epoxy-2α-methoxy-12α-hydroxy-6-oxogermacra-9α及β标明.png]

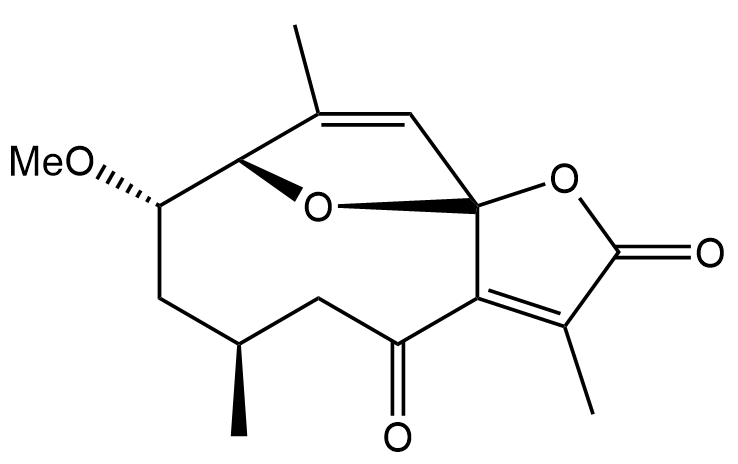

Supplement: Supplementary file 1 [file pharmaceuticals-17-01524-s001.zip › Sesquiterpene chemical structure/1β,8β-epoxy-2α-methoxy-6-oxogermacra-9(10),7(11)-dien.png]

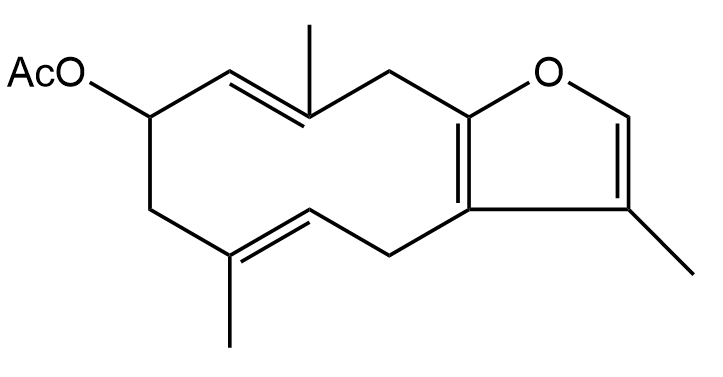

Supplement: Supplementary file 1 [file pharmaceuticals-17-01524-s001.zip › Sesquiterpene chemical structure/2-acetoxyfuranodiene.png]

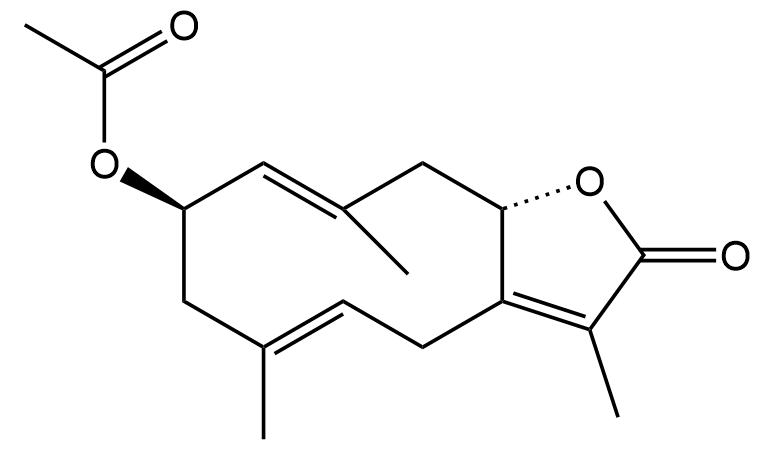

Supplement: Supplementary file 1 [file pharmaceuticals-17-01524-s001.zip › Sesquiterpene chemical structure/2-acetyloxyglechomanolide.png]

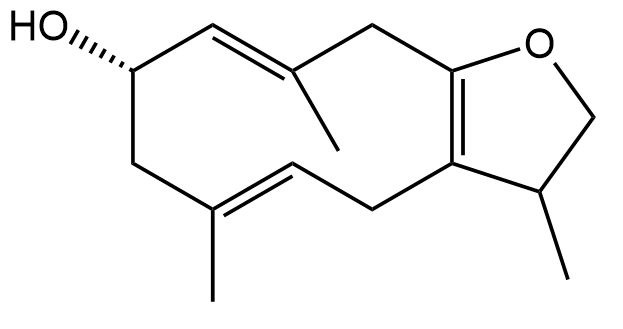

Supplement: Supplementary file 1 [file pharmaceuticals-17-01524-s001.zip › Sesquiterpene chemical structure/2-hydroxy-11,12-dihydrofuranodiene.png]

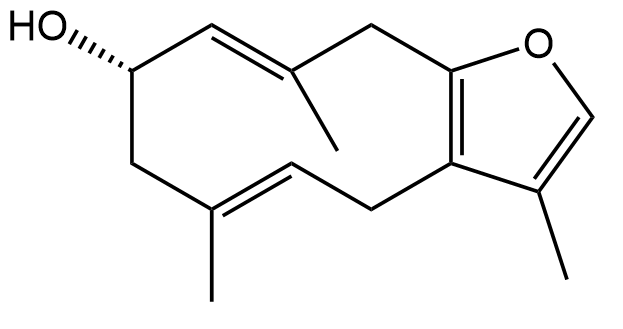

Supplement: Supplementary file 1 [file pharmaceuticals-17-01524-s001.zip › Sesquiterpene chemical structure/2-hydroxy-furanodiene.png]

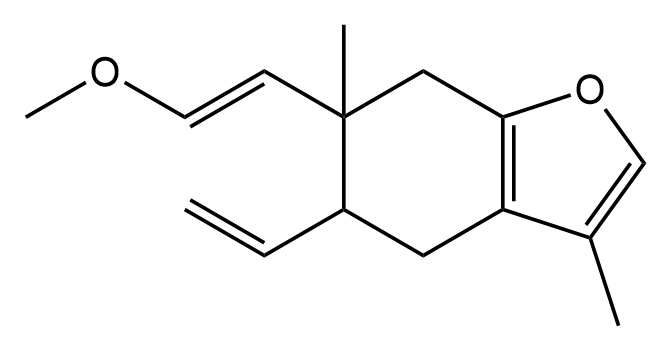

Supplement: Supplementary file 1 [file pharmaceuticals-17-01524-s001.zip › Sesquiterpene chemical structure/2-methoxy isofuranogermacrene.png]

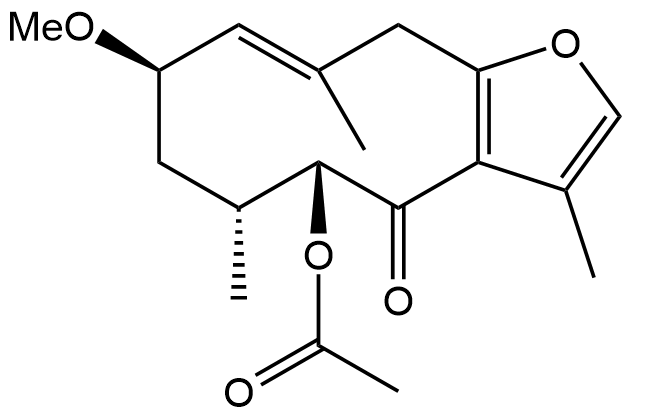

Supplement: Supplementary file 1 [file pharmaceuticals-17-01524-s001.zip › Sesquiterpene chemical structure/2-methoxy-5-acetoxy-fruranogermacr-1(10)-en-6-one.png]

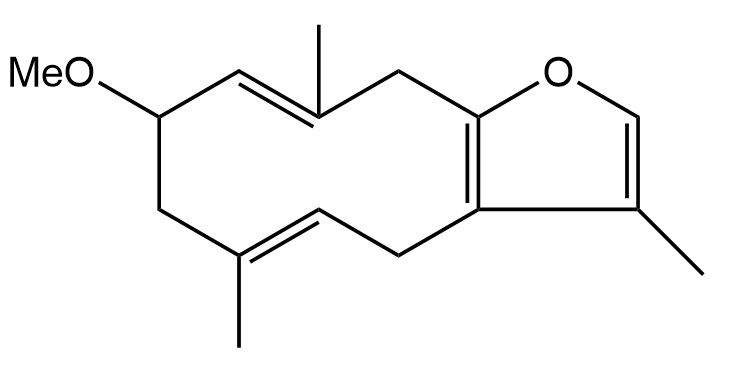

Supplement: Supplementary file 1 [file pharmaceuticals-17-01524-s001.zip › Sesquiterpene chemical structure/2-methoxyfuranodiene.png]

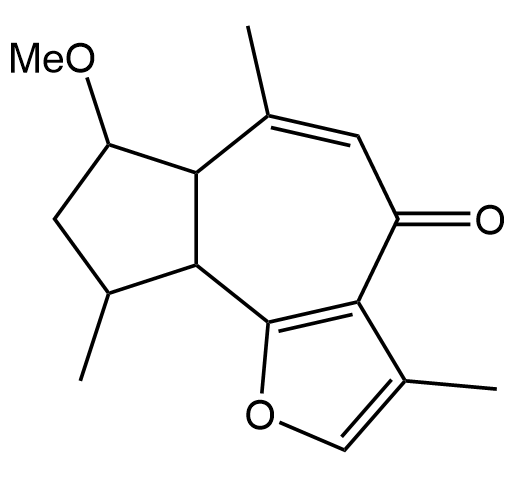

Supplement: Supplementary file 1 [file pharmaceuticals-17-01524-s001.zip › Sesquiterpene chemical structure/2-methoxyfuranoguaia-9-ene-8-one.png]

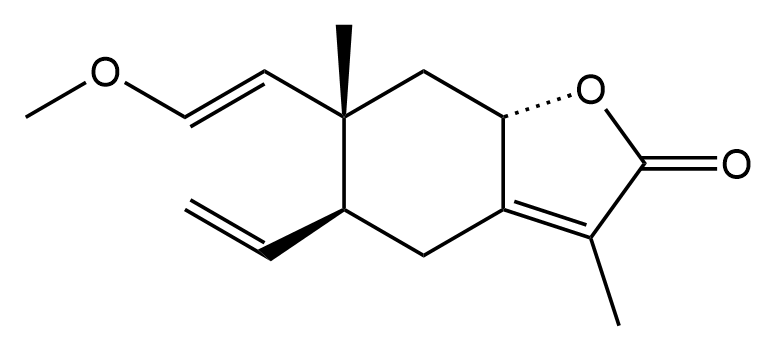

Supplement: Supplementary file 1 [file pharmaceuticals-17-01524-s001.zip › Sesquiterpene chemical structure/2-methoxyisogermafurenolide.png]

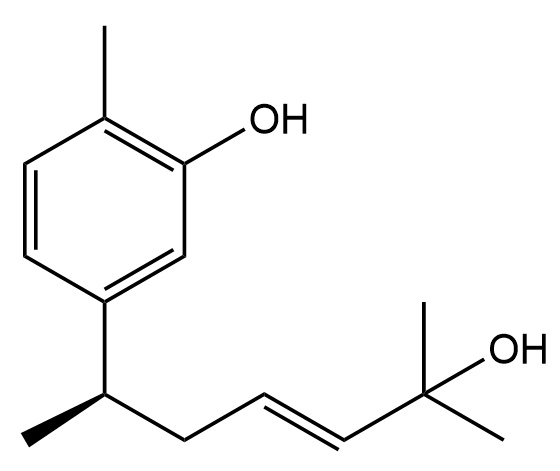

Supplement: Supplementary file 1 [file pharmaceuticals-17-01524-s001.zip › Sesquiterpene chemical structure/2-methyl-5-(5′-hydroxy-1′,5′-dimethyl-3′-hexenyl)phenol.png]

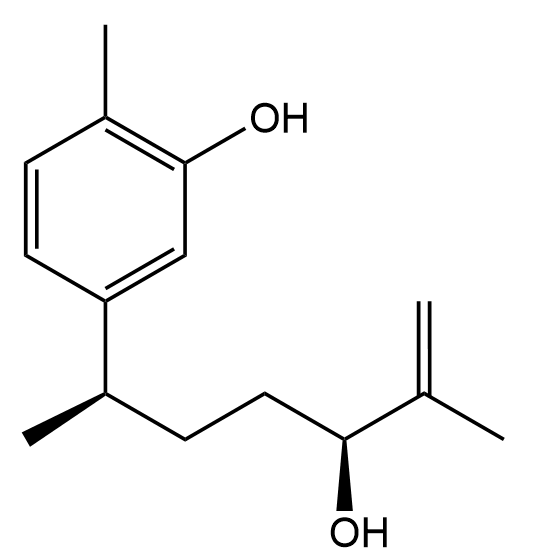

Supplement: Supplementary file 1 [file pharmaceuticals-17-01524-s001.zip › Sesquiterpene chemical structure/2-methyl-5-[4′(S)-hydroxy-1′(R),5′-dimethylhex-5′.png]

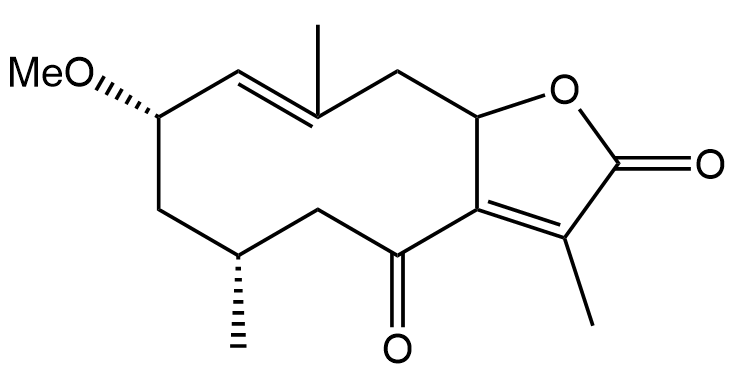

Supplement: Supplementary file 1 [file pharmaceuticals-17-01524-s001.zip › Sesquiterpene chemical structure/2α-methoxy-6-oxogermacra-1(10),7(11)-dien-8,12-olide.png]
